# A synthetic DNA template for fast manufacturing of versatile single epitope mRNA

**DOI:** 10.1016/j.omtn.2022.08.021

**Published:** 2022-08-17

**Authors:** Wout de Mey, Phaedra De Schrijver, Dorien Autaers, Lena Pfitzer, Bruno Fant, Hanne Locy, Arthur Esprit, Lien Lybaert, Cedric Bogaert, Magali Verdonck, Kris Thielemans, Karine Breckpot, Lorenzo Franceschini

**Affiliations:** 1 Laboratory for Molecular and Cellular Therapy, Department of Biomedical Sciences, Vrije Universiteit Brussel, Laarbeeklaan 103/E, 1090 Brussels, Belgium; 2 myNEO, Ottergemsesteenweg-Zuid 808, 9000 Ghent, Belgium

**Keywords:** MT: Oligonucleotides: Therapies and Applications, synthetic DNA template, mRNA, neoantigen, cancer, T cell

## Abstract

A flexible, affordable, and rapid vaccine platform is necessary to unlock the potential of personalized cancer vaccines in order to achieve full clinical efficiency. mRNA cancer vaccine manufacture relies on the rigid sequence design of multiepitope constructs produced by laborious bacterial cloning and time-consuming plasmid preparation. Here, we introduce a synthetic DNA template (SDT) assembly process, which allows cost- and time-efficient manufacturing of single (neo)epitope mRNA. We benchmarked SDT-derived mRNA against mRNA derived from a plasmid DNA template (PDT), showing that monocyte-derived dendritic cells (moDCs) electroporated with SDT-mRNA or PDT-mRNA, encoding HLA-I- or HLA-II-restricted (neo)epitopes, equally activated T cells that were modified to express the cognate T cell receptors. Furthermore, we validated the SDT-mRNA platform for neoepitope immunogenicity screening using the characterized HLA-A2-restricted neoepitope DHX40B and four new candidate HLA-A2-restricted melanoma neoepitopes. Finally, we compared SDT-mRNA with PDT-mRNA for vaccine development purposes. moDCs electroporated with mRNA encoding the HLA-A2-restricted, mutated Melan-A/Mart-1 epitope together with TriMix mRNA-generated high levels of functional Melan-A/Mart-1-specific CD8^+^ T cells. In conclusion, SDT single epitope mRNA can be manufactured in a more flexible, cost-efficient, and time-efficient way compared with PDT-mRNA, allowing prompt neoepitope immunogenicity screening, and might be exploited for the development of personalized cancer vaccines.

## Graphical abstract

**Figure:**
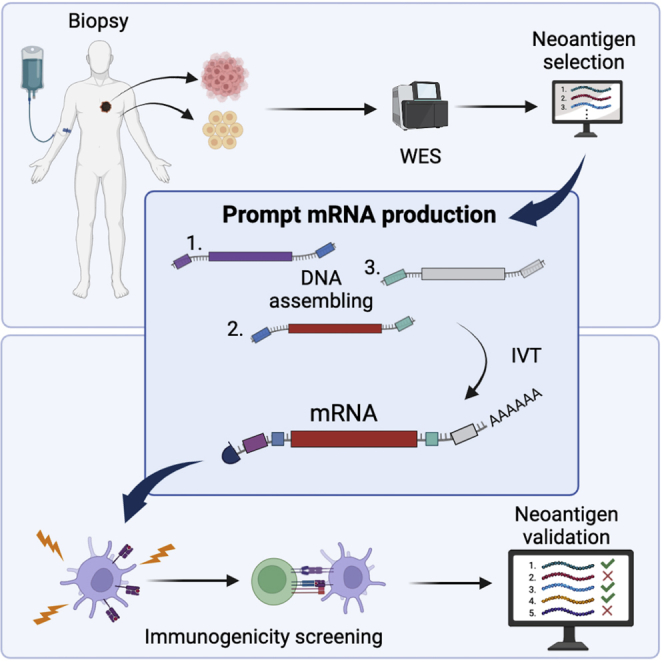

## Teaser

We developed a quick mRNA production platform, based on the use of a synthetic DNA template assembly process, allowing flexible, cost-efficient, and time-efficient manufacturing of single epitope mRNA libraries. The resulting SDT-mRNA can be used to screen neoepitope immunogenicity and might be exploited for the development of a personalized cancer vaccine.

## Introduction

The encouraging results achieved in the last decade with immune-checkpoint inhibitors (ICIs) have put immunotherapy on the front line of unconventional cancer treatments.^1^^,^^2^ However, studies have shown that only a subset of patients experience long-term clinical benefit from these new treatments.^3^ Therefore, many research groups are devoted to identifying strategies that act in synergy with ICIs.^4^ In this regard, cancer vaccination using tumor-specific neoantigens^5^, ^6^, ^7^ able to induce, expand, and broaden the tumor-directed T cell repertoire have shown promising results.^8^, ^9^, ^10^ Neoantigens are tumor-specific antigens, which originate from somatic mutations in the cancer cell genome and are not subjected to central tolerance.^11^ Advances in next-generation sequencing (NGS) have enabled fast identification of non-synonymous mutations resulting in neoantigens and at the same time allowing human leukocyte antigen (HLA) allele genotyping. Neoantigen identification is based on *in silico* screening and prioritization using bioinformatics pipelines that score each candidate neoantigen on parameters such as allele class presentation, peptide processing by the proteasome, T cell receptor (TCR) binding, major histocompatibility complex (MHC) affinity, peptide-MHC (pMHC) stability, and tumor neoantigen source.^12^^,^^13^

The main challenge is to identify, among the *in silico* predicted neoantigens, those able to trigger a robust immune response in the patient, as this could potentially result in the eradication of tumor cells presenting the neoantigens.^14^, ^15^, ^16^ Among the *in silico* identified neoantigen candidates, only a small number renders peptides (neoepitopes) that can be presented on MHC molecules on the cell surface, and only few of these pMHC complexes might be immunogenic, therefore eliciting a T cell response. For this reason, experimental validation of neoepitope immunogenicity is extremely important.^17^^,^^18^ Moreover, neoantigens are unique for each cancer patient, demanding a flexible and fast manufacturing platform to facilitate both the immunogenicity screening and subsequent personalized vaccine production. In this regard, mRNA is an attractive tool.^19^ Once in the cell, mRNA is translated into a polypeptide that contains the neoepitope sequence, which is further processed and degraded by the proteasome. Peptides released from proteasome degradation are transported to the endoplasmic reticulum and loaded onto MHC molecules, forming pMHC complexes. These pMHC complexes are transferred to the cell surface for presentation to the TCR of T cells.^20^

mRNA is transcribed from a DNA template,^21^ which contains the protein sequence aligned between a start and stop codon, indicated as the open reading frame (ORF). Besides the protein sequence, the template also contains untranslated sequences. These include the T7 promoter sequence, a binding site for the T7 polymerase to initiate the mRNA transcription enzymatic reaction from the DNA template,^22^ a Kozak sequence for the eukaryotic translation initiation,^23^^,^^24^ untranslated regions (UTRs) at the 5′ and 3′ end of the ORF sequence,^25^, ^26^, ^27^, ^28^ and a poly(A) tail at the 3′ end.^29^ UTRs and the poly(A) tail are key for mRNA stability and translatability, and as a result of enhanced biological activity. *In vitro* transcription (*iV*T) of mRNA requires a DNA template, for which often a plasmid DNA is used, produced by microbial fermentation, here indicated as plasmid DNA template (PDT).^30^^,^^31^ However, cloning and further preparation of PDT is a money-consuming, multistep process that takes from several days even up to weeks, involving the use of bacteria and antibiotics, without a guarantee that the correct bacterial clone will be identified and isolated for a long time.^32^ Moreover, the use of genetically modified antibiotic-resistant bacteria and the risk of biocontamination in the final product are serious concerns for the good manufacturing practice (GMP) development of mRNA-based vaccines.^33^ Here we introduce a novel, straightforward, and flexible approach for cost- and time-efficient generation of mRNA encoding single-antigen-derived peptides (epitopes). This approach is based on a synthetic DNA template (SDT) generated by assembly polymerase chain reaction (aPCR) using synthetic oligonucleotides as starting material. The resulting SDT is sequence verified and suited for *iV*T of mRNA. In the present work we showed that SDT-mRNA, similarly to PDT-mRNA, is presented to T cells after electroporation into monocyte-derived dendritic cells (moDCs) and that SDT-mRNA can be used to screen the immunogenicity of neoepitopes as well as develop cancer vaccines.

## Results

### Synthetic DNA template for *in vitro* mRNA transcription

**Figure 1:**
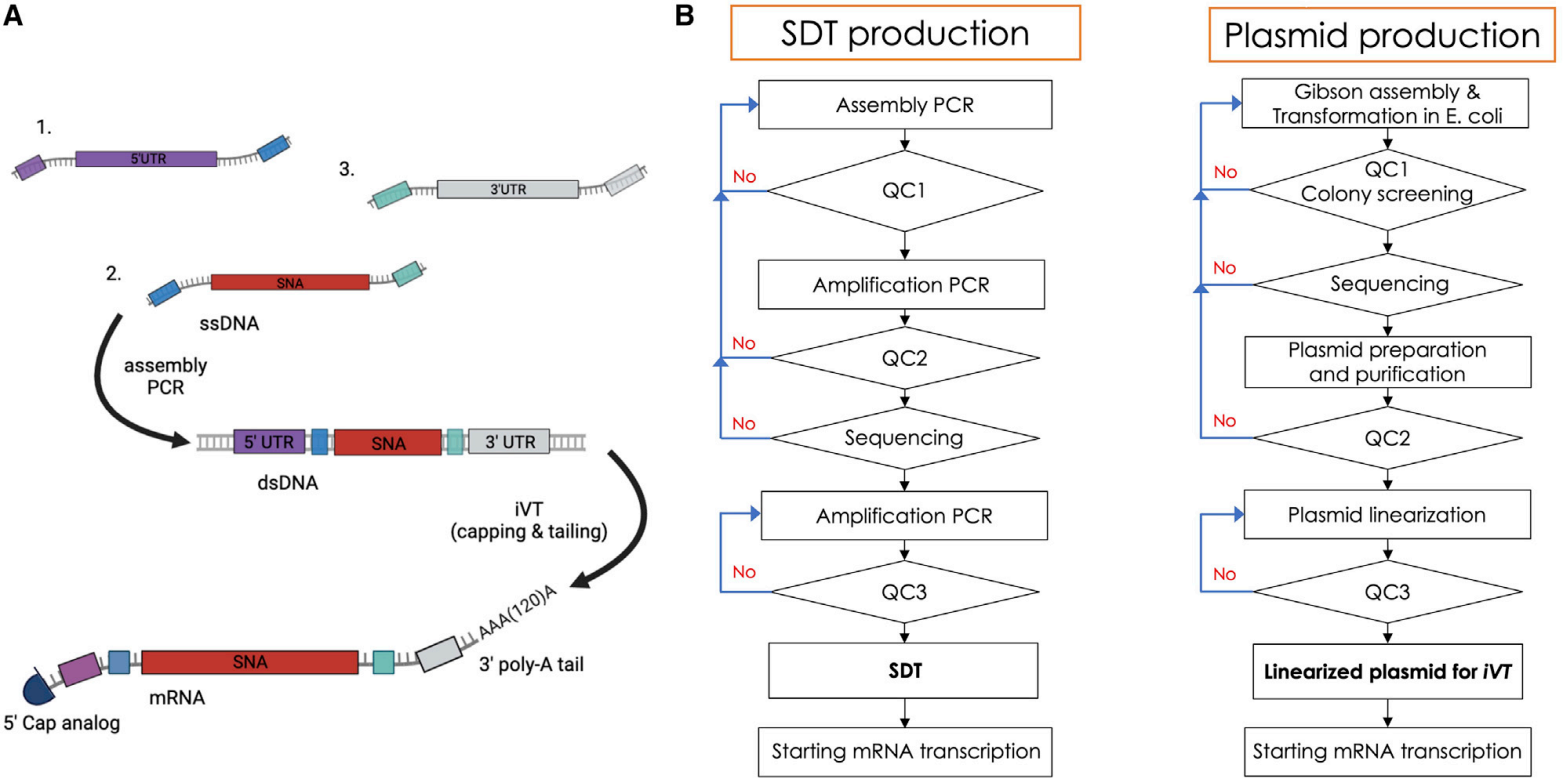
An SDT for *in vitro* synthesis of epitope encoding mRNA (A) Schematic representation of the SDT assembly reaction and mRNA synthesis: the SDT is formed by hybridization of three ssDNA molecules by aPCR: oligo 1 (purple) contains the T7 promoter, Kozak, and 5′ UTR; oligo 2 (red) encodes the cDNA to the mRNA of interest; and oligo 3 (gray) contains the 3′ UTR and a short poly(A) sequence. The resulting SDT is further amplified by PCR and used as template for *in vitro* transcription of mRNA. The mRNA is capped, and a poly(A) tail of ∼150 consecutive adenosine monophosphates is added during the enzymatic reaction. (B) Schematic representation of the workflow for mRNA production using an SDT (left) or a PDT (right). The DNA template and resulting mRNA are subjected to thorough quality controls (QC).

Three synthetic single-stranded DNA (ssDNA) molecules (<200 bases) were designed to hybridize together via aPCR, resulting in a synthetic double-stranded DNA (dsDNA) template that can be used for *iV*T of mRNA (Figure 1A). The SDT contains the sequence for the T7 promoter,^22^ Kozak sequence,^23^^,^^24^ and leader sequence from the human β-globin gene as 5′ UTR,^25^^,^^26^ a translation starting and stopping codon ATG/TGA respectively framing the ORF sequence of interest and the 3′ UTR derived from the goat β-globin protein.^27^ The ORF sequence encodes a 27-mer peptide, consisting of the immunodominant 9-mer epitope of interest. The ORF covers also the HLA-II sorting signal of dendritic cell (DC) lysosome-associated membrane protein (DC-LAMP, CD208) as this was shown to enhance presentation of HLA-I-restricted epitopes and to enable presentation of HLA-II-restricted epitopes.^34^ The resulting SDT can be amplified up to several micrograms by PCR. In one extensive enzymatic reaction and only in a few hours, it is possible to shift from DNA production to mRNA synthesis. Samples from each intermediate product can be sent for sequencing analysis and capillary gel electrophoresis (quality controls) (Figure 1B). Transcribed mRNA is capped, and an extensive poly(A) tail is added during the *iV*T reaction.^21^ We compared the manufacture of SDT-mRNA (Table S1) and PDT-mRNA (using the pLMCT plasmid, Table S2) encoding the published HLA-DP4-restricted epitope of the shared antigen MAGE-A3 (TQHFVQENYLEY)^34^^,^^35^ and the published HLA-A2-restricted epitopes of the shared antigens gp100 (YLEPGPVTA),^36^, ^37^, ^38^, ^39^ NY-ESO-1 (SLLMWITQC),^40^, ^41^, ^42^ and p53 (LLGRNSFEV),^43^^,^^44^ and, moreover, the published mutated antigen Melan-A/Mart-1 (A27L) (ELAGIGILTV).^45^ We subjected the resulting DNA templates and mRNA to thorough quality controls, including visual inspection (clear, colorless solution), spectrophotometry readout (yield, purity [A_260/280_]), capillary gel electrophoresis (integrity), and sequence analysis (alignment with reference sequence). According to the quality control specifications, we observed that SDT and SDT-mRNA were of quality comparable with that of PDT and PDT-mRNA, respectively, for these parameters (Tables 1 and 2; Figure S1).

**Table 1:** Release criteria of the synthetic DNA template versus plasmid DNA template

Test	Method	Release criteria	DNA							
			gp100-A2		p53-A2		NY-ESO1-A2		MAGE-A3-DP4	
			SDT	PDT	SDT	PDT	SDT	PDT	SDT	PDT
Appearance	visual inspection	clear, colorless solution	V	V	V	V	V	V	V	V
Content	spectrophotometry	>0.1 (SDT) or 1 mg/mL ± 10% (PDT)	0.138 mg/mL	0.929 mg/mL	0.144 mg/mL	0.944 mg/mL	0.138 mg/mL	0.940 mg/mL	0.156 mg/mL	1.053 mg/mL
A_260/280_	spectrophotometry	1.8–2.0	1.9	1.8	1.9	1.8	1.9	1.8	1.9	1.8
Integrity	CGE-DNA	one peak matching the theoretical DNA length	S1.A	S1.B	S1.A	S1.B	S1.A	S1.B	S1.A	S1.B
Identity	DNA sequencing	100% alignment with the reference sequence	100%	100%	100%	100%	100%	100%	100%	100%

V, verified.

A_260/280_, ratio of the absorbance at 260 versus 280 nm; A2, restricted to HLA-A2; CGE, capillary gel electrophoresis; DP4, restricted to HLA-DP4.

**Table 2:** Release criteria of the mRNA manufactured using the synthetic DNA template versus plasmid DNA template

Test	Method	Release criteria	RNA							
			gp100-A2		p53-A2		NY-ESO1-A2		MAGE-A3-DP4	
			SDT	PDT	SDT	PDT	SDT	PDT	SDT	PDT
Appearance	visual inspection	clear, colorless solution	V	V	V	V	V	V	V	V
Content	spectrophotometry	1 mg/mL ± 10%	1.050 mg/mL	0.997 mg/mL	1,043 mg/mL	1.009 mg/mL	1.080 mg/mL	0.916 mg/mL	1.086 mg/mL	1.053 mg/mL
A_260/280_	spectrophotometry	>2.0	2.54	2.029	2.25	2.105	2.25	2.073	2.38	2.089
Integrity	CGE-RNA	one peak matching the theoretical RNA length	S1.C	S1.D	S1.C	S1.D	S1.C	S1.D	S1.C	S1.D
Identity	RNase digestion	no detectable RNA after RNase digestion on CGE	V	V	V	V	V	V	V	V
	cDNA sequencing	epitope identification	V	V	V	V	V	V	V	V
Tailing efficiency	CGE-RNA	SDT: peak shift from the theoretical DNA length	S1.A/S1.C		S1.A/S1.C		S1.A/S1.C		S1.A/S1.C	
		PDT: peak size matching theoretical RNA length		S1.D		S1.D		S1.D		S1.D
5′ Capping validation	ELISA	CD8^+^ TCR^+^ T cell activation in response to moDCs electroporated with 5′ capped and non-capped RNA	V	V	V	V	V	V	V	V

V, verified.

A_260/280_, ratio of the absorbance at 260 versus 280 nm; A2, restricted to HLA-A2; CGE, capillary gel electrophoresis; DP4, restricted to HLA-DP4; cDNA, copy DNA.

### Electroporation of dendritic cells with mRNA transcribed from a synthetic DNA template or a plasmid DNA template does not induce phenotypic maturation

**Figure 2:**
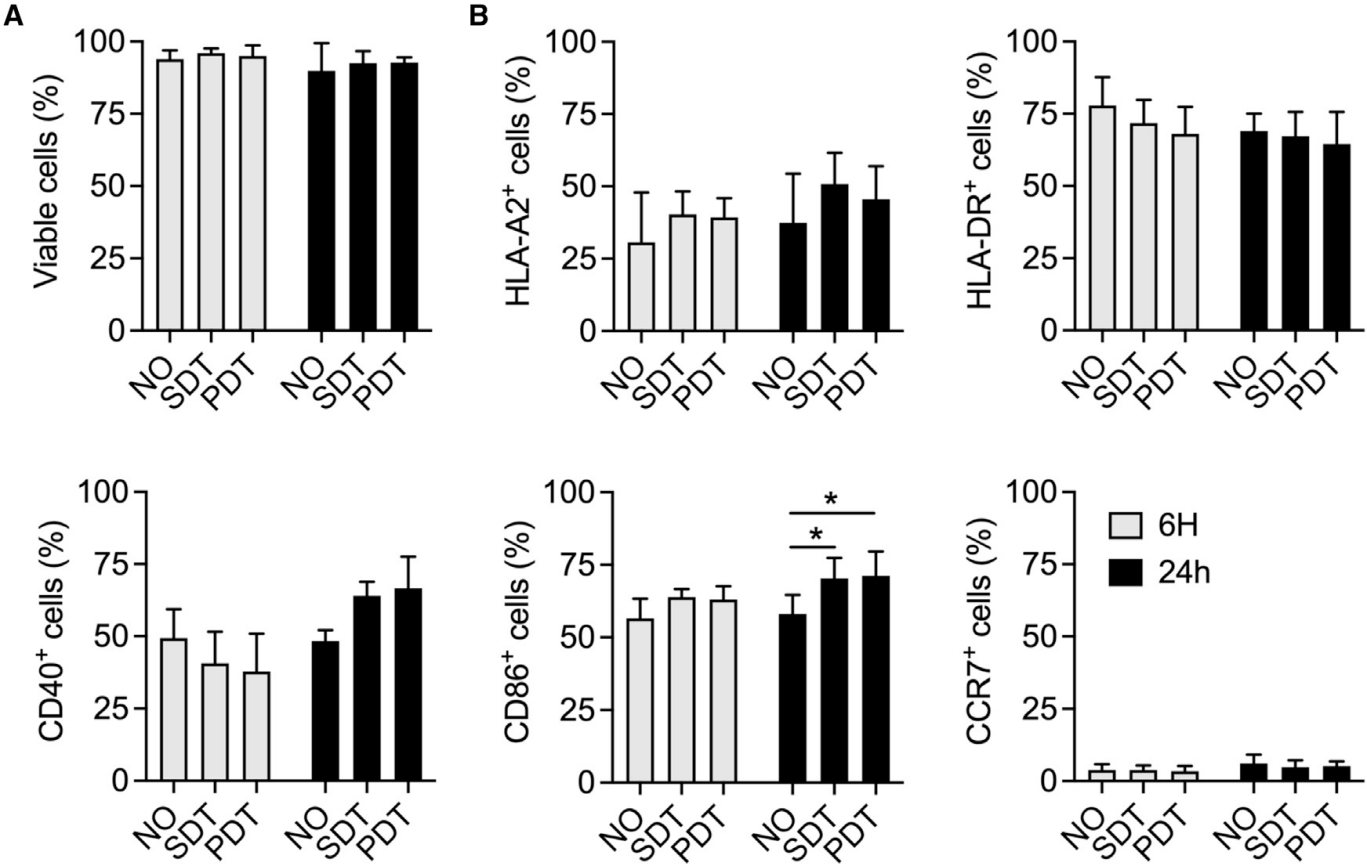
Viability and phenotype of dendritic cells electroporated with mRNA produced using a synthetic DNA template or a plasmid DNA template (A) Bar graph showing the viability of non-electroporated moDCs (NO) or moDCs electroporated with SDT-mRNA (SDT) or PDT-mRNA (PDT) expressed as percentage of viable cells. (B) Bar graphs showing the percentage of cells that express HLA-A2, HLA-DR, CD40, CD86, or CCR7 within the conditions: non-electroporated moDCs (NO) or moDCs electroporated with SDT-mRNA (SDT) or PDT-mRNA (PDT). Results are shown as mean ± standard error of the mean and summarize three independent experiments. One-way ANOVA with Bonferroni’s correction was performed to determine statistical significance, shown as ∗p < 0.05.

Electroporation has been extensively used to deliver mRNA into the cytosol of moDCs,^46^, ^47^, ^48^ thereby bypassing endosomes. As a result, pattern recognition receptors (PRRs) that could potentially detect mRNA, including Toll-like receptor 7 (TLR7) and TLR8,^49^ or that could potentially detect dsRNA impurities, including TLR3,^50^ are bypassed. However, several cytosolic PRRs, such as retinoic acid-inducible gene I (RIG-I), which could also sense dsRNA impurities, are not bypassed.^51^ Consequently, phenotypic maturation of moDCs after mRNA electroporation can be considered a result of sensing dsRNA impurities in the mRNA preparation. We electroporated moDCs with SDT-mRNA or PDT-mRNA and evaluated the moDC viability and phenotype at 6 h and 24 h after mRNA delivery. We did not observe changes in moDC viability after electroporation with SDT-mRNA or PDT-mRNA (Figure 2A). We further did not observe significant changes in the percentage of moDCs that expressed HLA-A2, HLA-DR, CD40, or CCR7 after electroporation with SDT-mRNA or PDT-mRNA, although we observed that the percentage of moDCs that expressed CD86 was significantly increased after electroporation with both SDT-mRNA and PDT-mRNA (Figure 2B).

### Enhanced presentation of (neo)epitopes to T cells by dendritic cells electroporated with (neo)epitope mRNA manufactured from a plasmid DNA template or synthetic DNA template

The faster, cheaper, and more flexible SDT-mRNA manufacturing platform could have far-reaching potential in vaccine development as a means to screen neoepitope immunogenicity. A prerequisite to unlock this potential is the presentation of the encoded neoepitope by SDT-mRNA electroporated DCs to T cells. Both CD4^+^ T helper 1 cells and CD8^+^ cytotoxic T cells are required to mount a potent antitumor immune response.^52^, ^53^, ^54^ However, mRNA that is transfected in DCs is translated into peptides that are processed for presentation onto HLA-I molecules and, hence, presentation to CD8^+^ T cells.^20^^,^^55^ Obtaining presentation of HLA-II-restricted epitopes, while simultaneously increasing the presentation of HLA-I-restricted epitopes upon delivery of mRNA-encoded antigens to moDCs, was shown to be possible by addition of the HLA-II sorting signal of DC-LAMP to the antigenic sequence.^34^ Therefore, this sorting signal was fused to the cDNA encoding a 27-mer peptide, consisting of the immunodominant 9-mer epitope under evaluation, which is flanked at the 5′ end and 3′ end by additional protein 9-mers. We evaluated presentation of the HLA-DP4-restricted epitope of the shared antigen MAGE-A3 (TQHFVQENYLEY)^34^^,^^35^ and the HLA-A2-restricted epitopes of the shared antigens gp100 (YLEPGPVTA),^36^, ^37^, ^38^, ^39^ NY-ESO-1 (SLLMWITQC),^40^, ^41^, ^42^ and p53 (LLGRNSFEV).^43^^,^^44^

**Figure 3:**
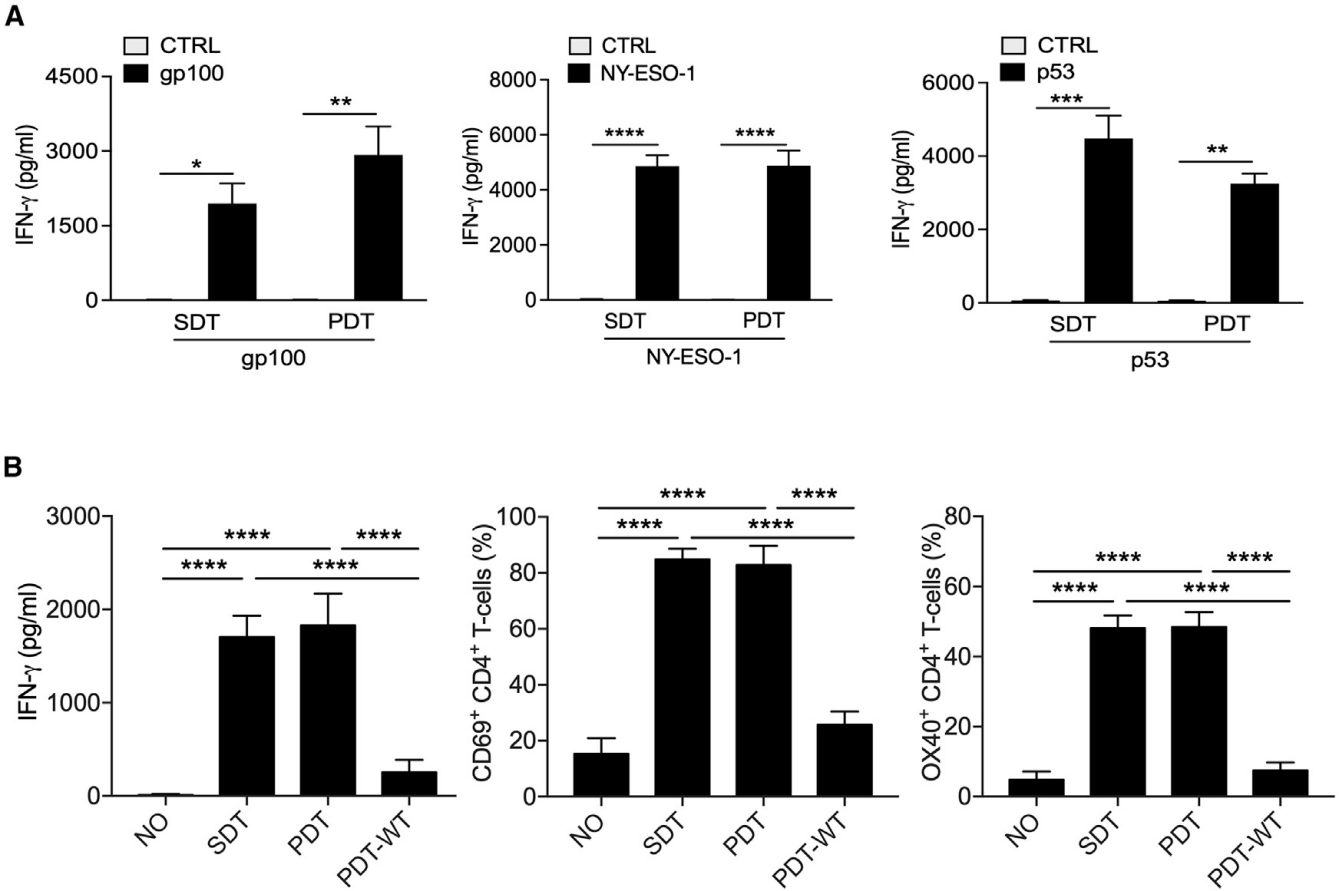
Antigen presentation by dendritic cells electroporated with epitope mRNA produced using a synthetic DNA template or a plasmid DNA template is similar (A) Bar graphs showing production of IFN-γ, quantified by ELISA, by CD8^+^ TCR^+^ T cells in response to antigen presentation by moDCs that were electroporated with SDT-mRNA or PDT-mRNA encoding the HLA-A2-restricted epitope of gp100 (left), NY-ESO-1 (middle), or p53 (right). The data are representative of results obtained in three independent experiments using cells of two different donors. (B) Bar graphs showing production of IFN-γ, quantified by ELISA (left), or the percentage of cells expressing the activation marker CD69 (middle) or OX40 (right), quantified in flow cytometry within CD4^+^ TCR^+^ T cells in response to antigen presentation by moDCs that were electroporated with SDT-mRNA or PDT-mRNA encoding the HLA-DP4-restricted and HLA-II-targeted epitope of MAGE-A3. As controls, T cells were co-cultured with non-electroporated moDCs (NO) or moDCs electroporated with mRNA encoding the non-HLA-II-targeted MAGE-A3 epitope (PDT-WT). The data are representative of results obtained in three independent experiments using cells of one donor. The results shown in (A) and (B) are summarized as mean ± standard error of the mean. One-way ANOVA with Bonferroni’s correction was performed to determine statistical significance, shown as follows: ∗p < 0.05, ∗∗p < 0.01, ∗∗∗p < 0.001, ∗∗∗∗p < 0.0001.

We electroporated HLA-A2^+^ moDCs with SDT-mRNA or PDT-mRNA and co-cultured them at a 1:1 ratio with CD8^+^ T cells that were electroporated with mRNA encoding the corresponding TCRα and TCRβ chain to test antigen presentation to CD8^+^ T cells (Table S3). Prior to the culture, we verified TCR expression on CD8^+^ T cells by flow cytometry (Figure S3). We quantified the amount of interferon-γ (IFN-γ) that was produced by the T cells during the 24-h co-culture in ELISA as a measure of antigen presentation by moDCs to the CD8^+^ T cells. As a control, we set up identical co-cultures with T cells that were not modified. We only observed production of IFN-γ when antigen-presenting moDCs were co-cultured with CD8^+^ T cells modified to express the corresponding TCR without statistically significant differences between antigen presentation by moDCs electroporated with SDT-mRNA or PDT-mRNA (Figure 3A).

We established similar co-cultures to study presentation of the HLA-DP4-restricted MAGE-A3 epitope by SDT-mRNA or PDT-mRNA electroporated moDCs to CD4^+^ T cells that were electroporated with mRNA encoding the corresponding TCRα and TCRβ chain.^35^ As a measure of antigen presentation by moDCs to these CD4^+^ T cells, we quantified IFN-γ produced by the T cells during the 24-h co-culture using ELISA and further analyzed the expression of the T cell activation markers CD69 and OX40 using flow cytometry (Figure 3B). To confirm the necessity of the HLA-II sorting signal of DC-LAMP to achieve antigen presentation in HLA-II, we used moDCs electroporated with PDT-mRNA encoding the MAGE-A3 antigen without its fusion to the DC-LAMP sorting signal. We observed little T cell activation when moDCs were electroporated with PDT-mRNA encoding for the non-HLA-II-targeted MAGE-A3, while similar levels of T cell activation were obtained in co-cultures with moDCs electroporated with SDT-mRNA or PDT-mRNA encoding the HLA-II-targeted MAGE-A3, signifying comparable levels of antigen presentation only when the epitope is actively shuttled to HLA-II compartments (Figure 3B).

### mRNA manufactured from synthetic DNA template might be used for personalized vaccine development

**Figure 4:**
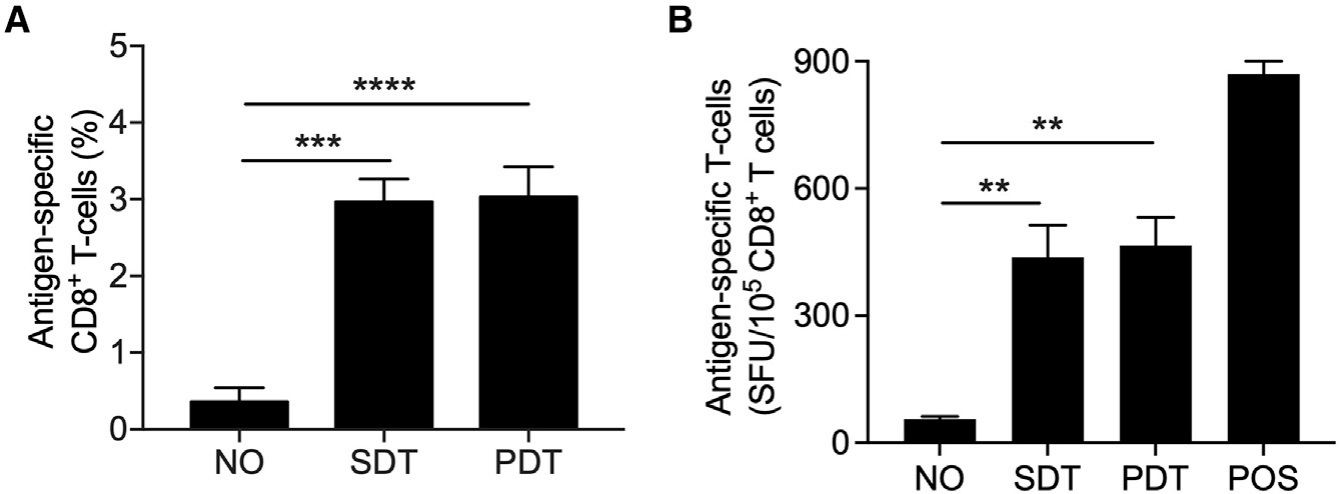
mRNA manufactured from a synthetic DNA template can be used for vaccine development (A) Bar graph showing the percentage of Melan-A/Mart-1 (A27L)-specific CD8^+^ T cells, quantified in flow cytometry, obtained after 10 days of co-culture with an autologous, HLA-A2^+^ moDC vaccine, consisting of moDCs co-electroporated with PDT-mRNA encoding TriMix, and PDT-mRNA of SDT-mRNA encoding the Melan-A/Mart-1 (A27L) epitope (fused to the sorting signal of DC-LAMP). As a control, CD8^+^ T cells were co-cultured with moDCs that were not modified to present the Melan-A/Mart-1 (A27L) epitope (NO). (B) Bar graph summarizing the number of SFU per 10^5^ CD8^+^ T cells, quantified using ELISPOT, after overnight restimulation of the activated CD8^+^ T cells with HLA-A2^+^ K562 cells presenting the Melan-A/Mart-1 (A27L) epitope. As a technical control, CD8^+^ T cells were activated with anti-CD3/CD28 antibody-coated beads (POS). The results in (A) are summarized as mean ± standard error of the mean of three independent experiments performed with cells of three different donors. The results in (B) are summarized as mean ± standard error of the mean of two independent experiments performed with cells of two different donors. One-way ANOVA with Bonferroni’s correction was performed to determine statistical significance, shown as follows: ∗∗p < 0.01, ∗∗∗p < 0.001, ∗∗∗∗p < 0.0001.

As we established that SDT-mRNA can be used to convert moDCs into (neo)epitope presenting cells, we next evaluated whether the SDT-mRNA could be used as an active ingredient for personalized vaccine development. Therefore, we generated HLA-A2^+^ moDCs and electroporated them with PDT-mRNA encoding the proprietary TriMix to endow the moDCs with strong T cell stimulatory capacity.^56^ We co-electroporated these moDCs with SDT-mRNA or PDT-mRNA encoding the mutated, highly immunogenic, HLA-A2-restricted Melan-A/Mart-1 (A27L) epitope^57^ and co-cultured them at a 1:2 ratio with autologous, naive (CD45RA^+^) CD8^+^ T cells for 10 days (Figure S3), according to the protocol described by Ali et al.^58^ We evaluated the percentage of activated CD8^+^ T cells in flow cytometry and their ability to produce IFN-γ using enzyme-linked immunospot assay (ELISPOT), measured by spot-forming units (SFU) per 10^5^ CD8^+^ T cells. We observed that HLA-A2^+^ moDCs presenting the Melan-A/Mart-1 (A27L) epitope after electroporation with SDT-mRNA or PDT-mRNA stimulated similarly high levels of functional Melan-A/Mart-1 (A27L)-specific CD8^+^ T cells (Figures 4A and 4B).

### Screening neoepitope immunogenicity using mRNA produced from a synthetic DNA template

**Figure 5:**
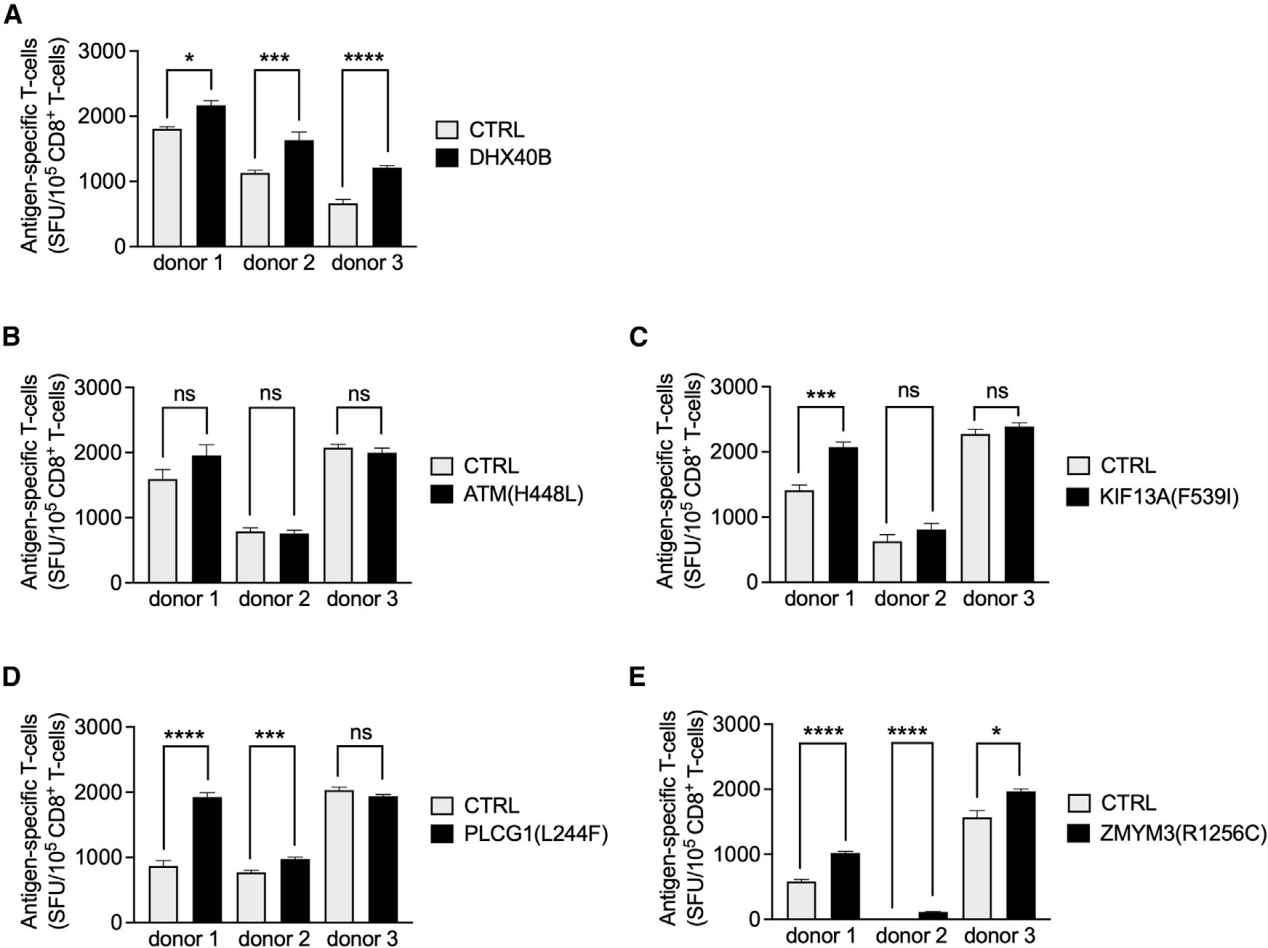
Screening of neoepitope immunogenicity is feasible using mRNA produced from a synthetic DNA template (A–E) Bar graphs summarizing the number of SFU per 10^5^ CD8^+^ T cells, quantified using ELISPOT, after overnight restimulation with HLA-A2^+^ K562 cells presenting (A) DHX40B, (B) ATM(H448L), (C) KIF13A(F539I), (D) PLCG1(L244F), or (E) ZMYM3(R1256C). Results are presented as mean ± standard error of the mean of triplicate samples for three independent experiments performed with cells of three different donors. Statistical significance was assessed using Student’s t test. Statistically significant differences are shown as follows: ∗p < 0.05, ∗∗∗p < 0.001, ∗∗∗∗p < 0.0001; ns, not significant.

Although advances in NGS techniques and computational pipelines have made *in silico* neoantigen prediction possible,^19^ each proposed candidate neoantigen should be experimentally validated to confirm its immunogenicity, i.e., its ability to trigger T cell activation.^15^^,^^16^^,^^59^ As SDT-mRNA encoding single epitopes can be manufactured in a time- and cost-reductive way and as electroporation of moDCs with single epitope encoding SDT-mRNA and TriMix mRNA results in antigen presentation and *de novo* stimulation of epitope-specific T cells, we used a similar setup to validate SDT-mRNA as a tool to screen the immunogenicity of neoepitopes. Using the proprietary ImmunoEngine bioinformatics pipeline developed by myNEO, 71 non-synonymous expressed somatic coding mutations were identified by whole-genome sequencing of a melanoma biopsy. Four of these neoantigens (ATM(H448L), KIF13A(F539I), PLCG1(L244F), ZMYM3(R1256C)) are predicted to be presented in HLA-A2 and were screened for their immunogenicity using moDCs from three HLA-A2^+^ donors and autologous naive (CD45RA^+^) CD8^+^ T cells (Table S1). An epitope that was previously described to be immunogenic (DHX40B) was used as a positive control.^60^ We observed a T cell response against DHX40B in all HLA-A2^+^ donors after two rounds of stimulation, confirming its immunogenicity and the validity of this screening approach using SDT-mRNA (Figure 5A). The neoantigen ATM(H448L) did not generate a T cell response in any of the donors, and we therefore considered this neoantigen non-immunogenic in this study. The neoantigens indicated as KIF13A(F539I), PLCG1(L244F), and ZMYM3(R1256C) generated a T cell response in one out of three, two out of three, and three out of three donors, respectively (Figure 5B).

## Discussion

Fast validation of immunogenic neoantigen candidates expressed and presented by tumor cells and, consequently, a prompt vaccine-manufacturing pipeline is necessary to develop personalized cancer vaccines.^14^, ^15^, ^16^ Identification of immunogenic neoepitopes is a major obstacle to the translation of neoantigen-based cancer immunotherapy into clinical studies.^61^, ^62^, ^63^ For *in vitro* studies to identify cognate TCRs and for the manufacture of neoantigen mRNA therapeutic vaccines, most often a cloning strategy is used in which a string of neoantigens are inserted into a plasmid. Also, personalized DNA-based vaccines or viral vectors most often encode a polyepitope construct.^31^^,^^64^, ^65^, ^66^ Several groups have been screening neoantigens through polyepitope constructs, using healthy donor HLA-matched peripheral blood mononuclear cells (PBMCs).^58^^,^^67^^,^^68^ Depending on the observed immunogenicity for each prioritized neoantigen, the polyepitope construct might undergo a design revision, resulting in a second round of cloning, which might delay this already laborious and time-consuming procedure. Furthermore, the design of the polyepitope construct could impact on epitope immunogenicity readout through effects introduced by the adjoining sequences within the polyepitope construct. Spacer sequences, in particular, which are commonly placed between each epitope, could lead to non-specific immunogenic effects. Moreover, the order of the epitopes within a polyepitope construct might also play a role in the observed immunogenicity and could induce competition within the polyepitope molecule owing to the prioritization based on epitope position.^58^^,^^69^^,^^70^ This might negatively affect the immunogenicity of screened neoantigens. Therefore, we developed a straightforward, cost- and time-reductive workflow to produce *in vitro* transcribed mRNA encoding for single (neo)epitopes starting from an SDT. The sequence design of the SDT-mRNA-encoded (neo)epitope includes a sorting signal of DC-LAMP, as this was shown to increase presentation of HLA-I-restricted peptides and as this was shown to be key to obtaining presentation of HLA-II-restricted peptides after their mRNA-mediated delivery,^34^^,^^71^ as confirmed in this study. Several studies have transcription of RNA from a plasmid-derived or synthetic PCR template.^72^^,^^73^ In this work, we used high-quality PAGE-purified oligonucleotides, within a price range similar to that of the gene fragments required for cloning of the control plasmids. Nevertheless, the manufacturing cost remains low because of the reduced operator working hours and the limited rental of facilities and necessary equipment linked herewith, owing to the quick SDT preparation process. The resulting SDT-mRNA is of quality comparable with that of the PDT-mRNA, as shown in the thorough quality control that evaluated yield, integrity, and purity. Moreover, the SDT-mRNA platform offers the advantage of fast, cost-efficient manufacturing of neoantigen mRNA without any risk of bioburden resulting from possible contamination with bacterial product traces or antibiotics. We showed that the SDT-mRNA and PDT-mRNA produced in this study and electroporated into moDCs did not impact on their cell viability and had little effect on their phenotype, with the exception of a small, though statistically significant, increase in CD86 expression. The latter might be a result of sensing of some dsRNA impurities by cytosolic dsRNA sensors,^51^ although this is of little concern given the proven antigen presentation by moDCs, which was comparable after electroporation with SDT-mRNA versus PDT-mRNA. As a result, the first prerequisite for use of SDT-mRNA for neoantigen immunogenicity screening and vaccine development is fulfilled. We showed that SDT-mRNA can indeed be used for both purposes using known mutated antigens, including DHX40 and Melan-A/Mart-1 (A27L), and four HLA-A2-restricted candidate neoantigens that were identified in melanoma tumor sample using NGS and the ImmunoEngine pipeline developed by myNEO. Therefore, we provided results showing that the SDT-mRNA platform is a cost- and time-reductive approach to generate neoepitope mRNA libraries that can be used to screen the immunogenicity of neoantigens and, moreover, that this might be exploited in the future to produce personalized mRNA batches suitable for a single patient dose in a very short time.

## Materials and methods

### Cell lines

The K562 cell line was purchased from the American Type Culture Collection. K562 cells were cultured in Iscove's modified Dulbecco's medium (IMDM) supplemented with 10% fetal bovine serum, 2 mM L-glutamine, 100 U/mL penicillin, and 100 μg/mL streptomycin, and maintained at 37°C in 5% CO_2_. The cell line was tested for the absence of mycoplasma contamination by PCR.

### Generation of monocyte-derived dendritic cells

Generation of moDCs was performed according to GMP. On day 0, a leukapheresis was performed on healthy donors at the Hematology Unit of the University Hospital in Brussels (UZ Brussel, Belgium) using an apheresis device (Spectra Optia apheresis system; Terumo BCT) to collect the PBMC fraction. This study was approved by the Ethical Committee of the UZ Brussel (2013/198). The leukapheresis product was further processed at the DC manufacturing unit of the Laboratory for Molecular and Cellular Therapy at the Vrije Universiteit Brussel (LMCT-VUB, Brussels, Belgium). An elutriation procedure (Elutra Cell Separation System; Terumo BCT) was performed to enrich monocytes. These monocytes were cultured in a cell culture bag with GMP-grade DC medium (CellGenix), supplemented with GMP-grade human serum albumin (1%; CAF-DCF) and GMP-grade cytokines: 500 IU/mL recombinant interleukin-4 (IL-4) (CellGenix) and 1,000 IU/mL recombinant granulocyte macrophage colony-stimulating factor (Leukine; Sanofi). Differentiation from monocyte to moDCs was allowed for 60–72 h by incubating the cells at 37°C and 5% CO_2_. The differentiated moDCs were harvested and, after count and viability assessment, cryopreserved in 5% DMSO cryopreservation medium (CryoStor CS5; BioLife Solutions). Cryovials were immediately transferred into a freezing container (CoolCell; Corning) and placed at −80°C. After overnight incubation at −80°C, vials were stored in the vapor phase of a liquid nitrogen container. From the same leukapheresis material, we stored the monocyte-depleted fraction as previously described.^74^

### Generation of DNA templates for *in vitro* mRNA transcription

The SDT was generated using three synthetic oligonucleotides (Ultramers; Integrated DNA Technologies [IDT]) that were designed to hybridize together during assembly PCR (KAPA HiFi HotStart ReadyMix; Roche), forming an SDT, which was further amplified by PCR. After each PCR, the formed SDT was purified (GeneJet PCR Purification Kit; Thermo Fisher Scientific). The PDT was generated using the plasmid pLMCT developed in-house. gBlocks for the different inserts were purchased from IDT and cloned into pLMCT using the Gibson assembly kit (New England Biolabs [NEB]) and XL2-Blue Ultracompetent Cells (Agilent). Cloned plasmids were sequence verified (Eurofins Genomics), and selected clones were further amplified by MIDI DNA preparation using plasmid kits from Qiagen. Each plasmid was linearized overnight by restriction enzyme digestion with BfuAI (NEB) to enable *in vitro* mRNA transcription. As quality controls, the yield (absorbance at 260/280 nm), integrity (BioAnalyzer 2100, DNA 7500 chip), and sequence (Eurofins Genomics) of both SDT and PDT were verified.

### mRNA synthesis

The *i*VT reaction was performed starting from a dsDNA template using a T7 enzyme mix containing: T7 RNA polymerase (Thermo Fisher Scientific), RNase inhibitor (Promega), and inorganic pyrophosphatase (Thermo Fisher Scientific). The reaction buffer mix included 10 mM Clean CAP AG reagent (TriLink Biotech) and 10 mM of each dNTP (adenosine-, guanosine-, cytidine- and uridine-triphosphate; Promega). The reaction was incubated at 37°C for 2 h. After incubation, DNaseI exonuclease (Thermo Fisher Scientific) was added to the reaction mix and incubated for 15 min at 37°C for the removal of residual dsDNA template. All enzymes added to the reaction were deactivated at 65°C for 20 min. For SDT-mRNA production, polymerase-A enzyme was added to the reaction mix for RNA polyadenylation (TebuBio poly(A) tailing kit). The reaction mix is incubated for 60 min at 37°C, after which 1.5 volumes of 40 mM EDTA solution were added to the mix to stop any further enzymatic activity. The mRNA was purified by LiCl-mediated precipitation. Half the reaction volume of 8 M LiCl (Sigma-Aldrich) was added to the mRNA solution and stored at −20°C overnight. The mRNA sample was centrifuged (15 min at 12,100 × *g*), and the obtained pellet was washed with 70% ethanol (Sigma-Aldrich) and subsequently dissolved in RNase-free water (Gibco). A second purification step was performed by NaCl/EtOH precipitation, adding 5 M NaCl (Sigma-Aldrich) and absolute ethanol (Sigma-Aldrich). The mRNA was centrifuged (15 min at 14,000 rpm), and the obtained pellet was washed with 70% ethanol and dissolved in RNase-free water (Gibco). The resulting SDT-mRNA and PDT-mRNA were subjected to quality controls, including spectrophotometric reading of optical density for the yield determination and purity (absorbance ratio at 260/280 nm), integrity (BioAnalyzer 2100, RNA 6500 chip), and cDNA sequence verification after reverse transcription (cDNA kit; NEB and Eurofins Genomics).

### Transfection of mRNA to cells by electroporation

Transfection of mRNA to moDCs and T cells was performed by electroporation. Cells were extensively washed in serum-free OptiMEM (Life Technologies, Belgium). The electroporation was performed in 200 μL of OptiMEM medium in a 4-mm electroporation cuvette (Cell Projects) using the following parameters: square wave pulse, 500 V, 2 ms, 1 pulse for moDCs; and square wave pulse, 500 V, 5 ms, 1 pulse for T cells, using the Gene Pulser Xcell device (Bio-Rad, Belgium). TCRα- and TCRβ-chain mRNA (5 μg each/10^6^ cells) was electroporated into CD8^+^ T cells. Electroporation of moDCs with mRNA was performed with a total concentration of 100 μg/mL mRNA.

### Antigen presentation assay

CD8^+^ T cells were isolated from monocyte-depleted PBMCs by magnetically activated cell sorting (MACS) using positive selection with human anti-CD8 microbeads according to the manufacturer’s instructions (Miltenyi Biotec). These CD8^+^ T cells, electroporated with TCR mRNA, were co-cultured with moDCs electroporated with the corresponding (neo)epitope mRNA at a 1:1 ratio in the presence of IL-2 (25 IU/mL, Thermo Fisher Scientific). Cells were plated in triplicate in 96-well round-bottom plates in IMDM supplemented with 1% human AB serum (200 μL/well) for 24 h at 37°C and 5% CO_2_. Supernatants from the co-cultured cells were collected to quantify IFN-γ in ELISA (Thermo Fisher Scientific) according to the manufacturer’s instructions.

### Stimulation of naive antigen-specific T cells

Naive CD8^+^ T cells were isolated from monocyte-depleted PBMCs by MACS using the CD8^+^ T cell isolation kit, with anti-CD45RO and anti-CD57 microbeads (Miltenyi Biotec). First the monocyte-depleted PBMCs were depleted from CD45RO- and CD57-positive cells, after which a positive selection was performed for CD8^+^ T cells. Cells were co-cultured with moDCs electroporated with TriMix and neoantigen mRNA at a 1:2 ratio in the presence of 30 ng/mL IL-21 (CellGenix). On days 3 and 7, IL-15 and IL-7 (Peprotech) were added at 5 ng/mL. In the case of stimulation of T cells recognizing the HLA-2-restricted, mutated Melan-A/Mart-1 (A27L) epitope, the T cells were collected and analyzed for Melan-A/Mart-1 (A27L) specificity with flow cytometry using HLA-A∗02:01/ELAGIGILTV (WB2162) dextramer staining performed according to the manufacturer’s instructions (Immudex, Denmark). Moreover, ELISPOT was performed to study IFN-γ production by the T cells after restimulation with HLA-A2^+^ and Melan-A/Mart-1 (A27L) presenting K562 cells. In the case of stimulation of T cells for candidate neoepitopes, including DHX40B, a restimulation of the CD8^+^ T cells was performed on day 7 using moDCs electroporated with SDT-mRNA encoding the neoepitope and PDT-mRNA encoding TriMix. On day 13, ELISPOT was performed to study IFN-γ production by the T cells after restimulation with HLA-A2^+^ and neoepitope presenting K562 cells.

### Flow cytometry

Cells were harvested and washed twice with PBS containing 1% BSA (flow cytometry buffer). The following antibody cocktail was used to phenotype moDCs: anti-CCR7-APC (clone G043H7, BioLegend), anti-CD40-BV605 (clone 5C3, BD), anti-CD86-FITC (clone FUN-1, BD Pharmingen), anti-HLA-DR-PE-CY7 (clone G46-6, BD Pharmingen), and anti-HLA-A2-BV421 (clone BB7.2, BioLegend). Cells were also stained with 7-AAD (BioLegend) to discriminate live from dead cells. T cell phenotyping was performed using the following antibodies: anti-CD8-BV421 (clone RPA-T8, BioLegend), anti-CD45RA-PE (clone HI100, BD), anti-CD45RO-APC (clone UCHL1, BD) and MHC dextramer Mel-A-PE or -APC (HLA-A∗0201/ELAGIGILTV, WB2162, Immudex) for CD8^+^ T cells and anti-CD4-FITC (clone OKT4, BioLegend), anti-CD69-PerCP-CY5.5 (clone FN50, BioLegend) and anti-OX40-APC (clone ACT35, BioLegend) for CD4^+^ T cells. T cells were also stained with 7-AAD (BioLegend) to discriminate live from dead cells. Cells were acquired on the LSR Fortessa flow cytometer and analyzed with FlowJo software, version 10.0.

### Enzyme-linked immunospot assay

Production of IFN-γ by T cells was measured by ELISPOT (Diaclone). T cells previously activated against HLA-A2-restricted neoepitopes were loaded onto ELISPOT plates at 30,000 T cells per well. Restimulation was performed at a 1:1 ratio with HLA-A2^+^ K562 cells electroporated with mRNA encoding the corresponding neoepitope or an unrelated antigen. After 24 h of co-culture, the ELISPOT plates were developed according to the manufacturer’s instructions to visualize spots, signifying IFN-γ-producing T cells. These were quantified as SFU with the aid of an ELISPOT reader (Autoimmun Diagnostika).

### Bioinformatics pipeline (ImmunoEngine)

Fresh frozen tumor biopsy material and blood was obtained from an anonymized melanoma patient. Tumor tissue was isolated by cryotome slicing and microdissection to ensure maximal tumor purity. Tumor DNA material (from microdissected tumor biopsy tissue) and DNA material from blood were extracted using the Qiagen DNeasy Blood & Tissue Kit. PCR-free whole-genome sequencing libraries were constructed using the NEBNext Ultra II FS DNA module and the NEBNext Ultra II Ligation module. Tumor RNA from microdissected tumor biopsy tissue was extracted using the Qiagen RNeasy Mini Kit, and a directional RNA-sequencing library was constructed using the NEBNext Ultra II Directional RNA Library Prep Kit for Illumina. All libraries were then sequenced on a Novaseq 6000 (v1.5). Sequencing results were subsequently analyzed by myNEO’s ImmunoEngine. In brief, sequencing data were aligned to the GRCh38 reference genome;^75^^,^^76^ blood and tumor sequencing data were then confronted using a combination of variant callers^77^^,^^78^ and custom filters to isolate a set of high-confidence, tumor-specific, DNA-borne single-nucleotide variants (SNVs) and insertion/deletions (indels) (n = 21,094 variants detected). Expression of these SNVs was further studied by assessing their presence in the tumor RNA-sequencing data^78^ (n = 1,072 variants significantly expressed). In a third step, the coding potential and neoantigen load of expressed SNVs was assessed^79^ (n = 71 non-synonymous expressed coding mutations), and the entire set of potential neoantigens was prioritized using a neoantigen score accounting for the following parameters: dissimilarity to self, predicted binding affinity rank as evaluated by MHCflurry,^80^ and predicted likelihood to elicit a T cell response as predicted by neoIM, a proprietary algorithm. Neoantigens with the highest score were selected for validation.

### Statistical analysis

All statistical analyses were performed using GraphPad Prism software, version 8.4.3. Statistical analysis on multiple datasets was performed with one-way ANOVA with Bonferroni’s correction, while statistical analysis for two datasets was performed using Student’s t test. Statistical analysis and significance is indicated in the figure legends.

### Data availability

The authors confirm that the data supporting the findings of this study are available within the article and its supplemental information.

## Supplemental information

Document S1. Tables S1–S3 and Figures S1–S3

Document S2. Article plus supplemental information
