# Supplementary material for: A synthetic DNA template for fast manufacturing of versatile single epitope mRNA
Source: Mol Ther Nucleic Acids. 2022 Aug 17;29:943–54. doi: 10.1016/j.omtn.2022.08.021 (PMC9464653; doi:10.1016/j.omtn.2022.08.021)
Supplement: Document S1. Tables S1–S3 and Figures S1–S3 [file mmc1.pdf]

OMTN, Volume *29*

Supplemental information

A synthetic DNA template for fast manufacturing of versatile single epitope mRNA

Wout de Mey, Phaedra De Schrijver, Dorien Autaers, Lena Pfitzer, Bruno Fant, Hanne Locy, Arthur Esprit, Lien Lybaert, Cedric Bogaert, Magali Verdonck, Kris Thielemans, Karine Breckpot, and Lorenzo Franceschini

Supplementary material

Table S1: Oligonucleotides used for synthetic DNA template production. Abbreviations: A2, restricted to HLA-A2; DP4, restricted to HLA-DP4; FW, forward; RV reverse; Oligo, oligonucleotide synthetic polymer.

Oligo 1 (FW 5′-3′)	TAATACGACTCACTATAGGAATACAAGCTACTTGTTCTTTTTGCATCACAACCAGGCCTCCACAACCATGGCGGCCCCTGGTAGTGCAAGGAGGCCCCTCCTGCTGCTTCTGT TGCTTTTGCTGTTAGGCTTGATGCAC
Oligo 3 (RV 5′-3′)	TTTTTTTTTGTCGACAATATACACTGGGATAAAAACCTTGCCGTCTGGTCACTGGAGGTGTCCGGTGCGAACAAGGAAAAACCCAGCCAAAACACTCGAGTCAAATCCTCTGAT AGCCTGAACTTTGACAGCGAAGGCGAATTTTATACACGCCCATGCCCATCAAGCAGAGGCCGACAACTATCGCTCC
Oligo 2 (gp100-A2 RV 5′-3′)	AGGCCGACAACTATCGCTCCTATAACTGGCAAAACAATAGTGTAGTCAGAAGATGGCGGGATGGCTGCTTGCAAAACGACCTGAGCAGTGACTGGGCCGGGTTCAAGGTACG TATGTGTGACAACCAGCGCTCTGCTAGCCGCAGAGGCACAGTGCATCAAGCCTAACAGCA
Oligo 2 (NY-ESO-1-A2 RV 5′-3′)	AGGCCGACAACTATCGCTCCTATAACTGGCAAAACAATAGTGTAGTCAGAAGATGGCGGTTGCGCCAGAAAGACCGGAAGAAAAACTTGTGTTATCCACATAAGCAGTGACAA TTGCTGCAAGCAAGAAGATATGCTAGCCGCAGAGGCACAGTGCATCAAGCCTAACAGCA
Oligo 2 (p53-A2 RV 5′-3′)	AGGCCGACAACTATCGCTCCTATAACTGGCAAAACAATAGTGTAGTCAGAAGATGGATCTCGGCCAGGACAGGCACAGACCCTAACTTCAAAACTGTTGCGTCCGAGCAAGTT ACCGCTACTATCCTCCAGAGTAATAGCCGCAGAGGCACAGTGCATCAAGCCTAACAGCA
Oligo 2 (MAGE-A3-DP4 RV 5′-3′)	AGGCCGACAACTATCGCTCCTATAACTGGCAAAACAATAGTGTAGTCAGAAGATGGGCCAGGTACTTGTCGATATTCCAAATAGTTTTCTTGTACGAAGTGTTGAGTGAGAAGT TTCTTTGGGTCTCCAAGGATGCTAGCCGCAGAGGCACAGTGCATCAAGCCTAACAGCA
Oligo 2 (Mel-A MUT-A2 RV 5′-3′)	AGGCCGACAACTATCGCTCCTATAACTGGCAAAACAATAGTGTAGTCAGAAGATGGGCCAATGAGCAACAGGACCCCAAGAATTACTGTCAGAATACCTATGCCAGCGAGTTC CTCGGCAGTCGTGTAACTATGGCCAGCCGCAGAGGCACAGTGCATCAAGCCTAACAGCA
Oligo 2 (DHX40b+C-A2 RV 5′-3′)	AGGCCGACAACTATCGCTCCTATAACTGGCAAAACAATAGTGTAGTCAGAAGATGGTCCTCTGCTAGACTCTCTCAGGGACACCAGGCCGTGCATCAGGATGCTCTTGTCGTC GTTCCGCCGCTGCATCTTAGCCGCAGAGGCACAGTGCATCAAGCCTAACAGCA
Oligo 2 (ATM(H448L)-A2 RV 5‘-3’)	AGGCCGACAACTATCGCTCCTATAACTGGCAAAACAATAGTGTAGTCAGAAGATGGCTCGGTCAGACATCTCAGCACGTAGGGTGTTCTCTCGCCCAGTCTCTGCTGAGGCAG CAGCTGGCTCAGGATCATCAGCAGAGCCGCAGAGGCACAGTGCATCAAGCCTAACAGCA
Oligo 2 (KIF13A(F539I)-A2 RV 5‘-3’)	AGGCCGACAACTATCGCTCCTATAACTGGCAAAACAATAGTGTAGTCAGAAGATGGCAGCCAATCTCTCCGCTTCCGCTTAGGCAGGTTGATCCGGATGAAGTGGTTGTTGCC CCACAGGATTCTATCGCCGTGCCAAGCCGCAGAGGCACAGTGCATCAAGCCTAACAGCA
Oligo 2 (PLCG1(L244F)-A2 RV 5‘-3’)	AGGCCGACAACTATCGCTCCTATAACTGGCAAAACAATAGTGTAGTCAGAAGATGGAAGTCTATCAACGGCCCACAGCTCGCCCTGGTAGTCCAGGAAGAACTGCTGGAACTC AGGCAGGGACACTCTGCACAGCTCAGCCGCAGAGGCACAGTGCATCAAGCCTAACAGCA
Oligo 2 (ZMYM3(R1256C)-A2 RV 5‘-3’)	AGGCCGACAACTATCGCTCCTATAACTGGCAAAACAATAGTGTAGTCAGAAGATGGTGTATCTCTGCCCTTTCTCTGCCGCACGGGAGCGTAGTAGCAGATGGACACCACCTT GGTGGTGCCTCTAGGTGTTGTGCAAGCCGCAGAGGCACAGTGCATCAAGCCTAACAGCA

Table S2: Sequence of the pLMCT (plasmid) and cDNA encoding the gene of interest (gBlock) used for plasmid DNA template production.

Abbreviations: A2, restricted to HLA-A2; DP4, restricted to HLA-DP4; FW, forward; RV reverse; SigDCL, HLA-II sorting signal of DC-LAMP.

pLMCT<br>Empty vector	TGACTCGAGTGTTTAAACCAGCCTCAAGAACACCCGAATGGAGTCTCTAAGCTACATAATACCAACTTACACTTTACAAAATGTTGTCCCCCAAAATGTAGCCATTCGTATCTGCTCCTAATAAAAAGAAA<br>GTTTCTTCACTATTGTCGACAAAAAAAAAAAAAAAAAAAAAAAAAAAAAAAAAAAAAAAAAAAAAAAAAAAAAAAAAAAAAAAAAAAAAAAAAAAAAAAAAAAAAAAAAAAAAAAAAAAAAAAAAA AAAAAA<br>GCAGGTGTGTCTCTCTCCACCTGCGAATTCACTGGCCGTCGTTTTACAACGTCGTGACTGGGAAAACCCTGGCGTTACCCAACTTAATCGCCTTGCAGCACATCCCCCTTTCGCCAGCTGGCGTAATAG<br>CGAAGAGGCCCGCACCGATCGCCCTTCCCAACAGTTGCGCAGCCTGAATGGCGAATGGCGCCTGATGCGGTATTTTCTCCTTACGCATCTGTGCGGTATTTCACACCGCATATGGTGCACTCTCAGTAC<br>AATCTGCTCTGATGCCGCATAGTTAAGCCAGCCCCGACACCCGCCAACACCCGCTGACGCGCCCTGACGGGCTTGTCTGCTCCCGGCATCCGCTTACAGACAAGCTGTGACCGTCTCCGGGAGCTGCA<br>TGTGTCAGAGGTTTTCACCGTCATCACCGAAACGCGCGAGACGAAAGGGCCTCGTGATACGCCTATTTTTATAGGTTAATGTCATGATAATAATGGTTTCTTAGACGTCAGGTGGCACTTTTCGGGGAAA<br>TGTGCGCGGAACCCCTATTTGTTTATTTTTCTAAATACATTCAAATATGTATCCGCTCATGAGACAATAACCCTGATAAATGCTTCAATAATATTGAAAAAGGAAGAGTATGAGTATTCAACATTTCCGTGT<br>CGCCCTTATTCCCTTTTTTGCGGCATTTTGCCTTCCTGTTTTTGCTCACCCAGAAACGCTGGTGAAAGTAAAAGATGCTGAAGATCAGTTGGGTGCACGAGTGGGTTACATCGAACTGGATCTCAACAGC<br>GGTAAGATCCTTGAGAGTTTTCGCCCCGAAGAACGTTTTCCAATGATGAGCACTTTTAAAGTTCTGCTATGTGGCGCGGTATTATCCCGTATTGACGCCGGGCAAGAGCAACTCGGTCGCCGCATACAC<br>TATTCTCAGAATGACTTGGTTGAGTACTCACCAGTCACAGAAAAGCATCTTACGGATGGCATGACAGTAAGAGAATTATGCAGTGCTGCCATAACCATGAGTGATAACACTGCGGCCAACTTACTTCTGA<br>CAACGATCGGAGGACCGAAGGAGCTAACCGCTTTTTTGCACAACATGGGGGATCATGTAACTCGCCTTGATCGTTGGGAACCGGAGCTGAATGAAGCCATACCAAACGACGAGCGTGACACCACGATG<br>CCTGTAGCAATGGCAACAACGTTGCGCAAACTATTAACTGGCGAACTACTTACTCTAGCTTCCCGGCAACAATTAATAGACTGGATGGAGGCGGATAAAGTTGCAGGACCACTTCTGCGCTCGGCCCTT<br>CCGGCTGGCTGGTTTATTGCTGATAAATCTGGAGCCGGTGAGCGTGGGTCTCGCGGTATCATTGCAGCACTGGGGCCAGATGGTAAGCCCTCCCGTATCGTAGTTATCTACACGACGGGGAGTCAGGC<br>AACTATGGATGAACGAAATAGACAGATCGCTGAGATAGGTGCCTCACTGATTAAGCATTGGTAACTGTCAGACCAAGTTTACTCATATATACTTTAGATTGATTTAAAACTTCATTTTTAATTTAAAAGGAT<br>CTAGGTGAAGATCCTTTTTGATAATCTCATGACCAAAATCCCTTAACGTGAGTTTTCGTTCCACTGAGCGTCAGACCCCGTAGAAAAGATCAAAGGATCTTCTTGAGATCCTTTTTTTCTGCGCGTAATCT<br>GCTGCTTGCAAACAAAAAAACCACCGCTACCAGCGGTGGTTTGTTTGCCGGATCAAGAGCTACCAACTCTTTTTCCGAAGGTAACTGGCTTCAGCAGAGCGCAGATACCAAATACTGTTCTTCTAGTGTA<br>GCCGTAGTTAGGCCACCACTTCAAGAACTCTGTAGCACCGCCTACATACCTCGCTCTGCTAATCCTGTTACCAGTGGCTGCTGCCAGTGGCGATAAGTCGTGTCTTACCGGGTTGGACTCAAGACGATA<br>GTTACCGGATAAGGCGCAGCGGTCGGGCTGAACGGGGGGTTCGTGCACACAGCCCAGCTTGGAGCGAACGACCTACACCGAACTGAGATACCTACAGCGTGAGCTATGAGAAAGCGCCACGCTTCCC<br>GAAGGGAGAAAGGCGGACAGGTATCCGGTAAGCGGCAGGGTCGGAACAGGAGAGCGCACGAGGGAGCTTCCAGGGGGAAACGCCTGGTATCTTTATAGTCCTGTCGGGTTTCGCCACCTCTGACTT<br>GAGCGTCGATTTTTGTGATGCTCGTCAGGGGGGCGGAGCCTATGGAAAAACGCCAGCAACGCGGCCTTTTTACGGTTCCTGGCCTTTTGCTGGCCTTTTGCTCACATGTTCTTTCCTGCGTTATCCCCTG<br>ATTCTGTGGATAACCGTATTACCGCCTTTGAGTGAGCTGATACCGCTCGCCGCAGCCGAACGACCGAGCGCAGCGAGTCAGTGAGCGAGGAAGCGGAAGAGCGCCCAATACGCAAACCGCCTCTCCC<br>CGCGCGTTGGCCGATTCATTAATGCAGCTGGCACGACAGGTTTCCCGACTGGAAAGCGGGCAGTGAGCGCAACGCAATTAATGTGAGTTAGCTCACTCATTAGGCACCCCAGGCTTTACACTTTATGCT<br>TCCGGCTCGTATGTTGTGTGGAATTGTGAGCGGATAACAATTTCACACAGGAAACAGCTATGACCATGATTACGCCAAGCTTTAATACGACTCACTATAAGGAATACAAGCTACTTGTTCTTTTTGCATCA<br>CAACCAGGCCTCCACAACCATG
p53-A2 SigDCL	CTTGTTCTTTTTGCATCACAACCAGGCCTCCACAACCATGGCGGCCCCTGGTAGTGCAAGGAGGCCCCTCCTGCTGCTTCTGTTGCTTTTGCTGTTAGGCTTGATGCACTGTGCCTCTGCGGCTATTACT<br>CTGGAGGATAGTAGCGGTAACTTGCTCGGACGCAACAGTTTTGAAGTTAGGGTCTGTGCCTGTCCTGGCCGAGATCCATCTTCTGACTACACTATTGTTTTGCCAGTTATAGGAGCGATAGTTGTCGGC<br>CTCTGCTTGATGGGCATGGGCGTGTATAAAATTCGCCTTCGCTGTCAAAGTTCAGGCTATCAGAGGATTTGATAACTCGAGTGTTTAAACCAGCCTCAAGAACACCCGAATGGAG
gp100-A2 SigDCL	CTTGTTCTTTTTGCATCACAACCAGGCCTCCACAACCATGGCGGCCCCTGGTAGTGCAAGGAGGCCCCTCCTGCTGCTTCTGTTGCTTTTGCTGTTAGGCTTGATGCACTGTGCCTCTGCGGCTAGCAGA<br>GCGCTGGTTGTCACACATACGTACCTTGAACCCGGCCCAGTCACTGCTCAGGTCGTTTTGCAAGCAGCCATCCCGCCATCTTCTGACTACACTATTGTTTTGCCAGTTATAGGAGCGATAGTTGTCGGCC<br>TCTGCTTGATGGGCATGGGCGTGTATAAAATTCGCCTTCGCTGTCAAAGTTCAGGCTATCAGAGGATTTGATAACTCGAGTGTTTAAACCAGCCTCAAGAACACCCGAATGGAG
NY-ESO-1-A2 SigDCL	CTTGTTCTTTTTGCATCACAACCAGGCCTCCACAACCATGGCGGCCCCTGGTAGTGCAAGGAGGCCCCTCCTGCTGCTTCTGTTGCTTTTGCTGTTAGGCTTGATGCACTGTGCCTCTGCGGCTAGCATA<br>TCTTCTTGCTTGCAGCAATTGTCACTGCTTATGTGGATAACACAAGTTTTTCTTCCGGTCTTTCTGGCGCAACCGCCATCTTCTGACTACACTATTGTTTTGCCAGTTATAGGAGCGATAGTTGTCGGCCT<br>CTGCTTGATGGGCATGGGCGTGTATAAAATTCGCCTTCGCTGTCAAAGTTCAGGCTATCAGAGGATTTGATAACTCGAGTGTTTAAACCAGCCTCAAGAACACCCGAATGGAG

Melan-A MUT-A2 SigDCL	CTTGTTCTTTTTGCATCACAACCAGGCCTCCACAACCATGGCGGCCCCTGGTAGTGCAAGGAGGCCCCTCCTGCTGCTTCTGTTGCTTTTGCTGTTAGGCTTGATGCACTGTGCCTCTGCGGCTGGCCAT<br>AGTTACACGACTGCCGAGGAACTCGCTGGCATAGGTATTCTGACAGTAATTCTTGGGGTCCTGTTGCTCATTGGCCCATCTTCTGACTACACTATTGTTTTGCCAGTTATAGGAGCGATAGTTGTCGGCC<br>TCTGCTTGATGGGCATGGGCGTGTATAAAATTCGCCTTCGCTGTCAAAGTTCAGGCTATCAGAGGATTTGATAACTCGAGTGTTTAAACCAGCCTCAAGAACACCCGAATGGAG
MAGE-A3-DP4 SigDCL	CTTGTTCTTTTTGCATCACAACCAGGCCTCCACAACCATGAGCATCCTTGGAGACCCAAAGAAACTTCTCACTCAACACTTCGTACAAGAAAACTATTTGGAATATCGACAAGTACCTGGCTGATAACTCG<br>AGTGTTTAAACCAGCCTCAAGAACACCCGAATGGAG

Table S3: Sequence of the gBlocks used to clone pLMCT plasmids encoding T-cell receptor alpha or beta chains. Abbreviations: A2, restricted to HLA-A2; DP4, restricted to HLA-DP4; TCRα, T-cell receptor alpha chain; TCRβ, T-cell receptor beta chain.

p53-A2, TCRα	ACTTGTTCTTTTTGCATCACAACCAGGCCTCCACAACCATGTTACTGGCGCTCCTCCCAGTGCTGGGGATACACTTTGTCCTGAGAGATGCCCAAGCTCAGTCAGTGACGCAGCCCGATGCTCGCGTCACTGTC<br>TCTGAAGGAGCCTCTCTGCAGCTGAGATGCAAGTATTCCTACTCTGGGACACCTTATCTGTTCTGGTATGTCCAGTACCCGCGGCAGGGGCTGCAGCTGCTCCTCAAGTACTATTCAGGAGACCCAGTGGTTCA<br>AGGAGTGAATGGCTTCGAGGCTGAGTTCAGCAAGAGTAACTCTTCCTTTCATCTGCGGAAAGCCTCTGTGCACTGGAGCGACTCTGCTGTGTACTTCTGTGTTTTGAGCGAGGATAGCAACTATCAGTTGATCT<br>GGGGCTCTGGGACCAAGCTAATTATAAAGCCAGACATCCAGAACCCAGAGCCTGCTGTGTACCAGTTAAAAGATCCTCGGTCTCAGGACAGCACCCTCTGCCTGTTCACCGACTTTGACTCCCAAATCAATGTG<br>CCGAAAACAATGGAATCTGGAACGTTCATCACTGACAAAACTGTGCTGGACATGAAAGCTATGGATTCCAAGAGCAATGGGGCCATTGCCTGGAGCAACCAGACAAGCTTCACATGCCAAGATATCTTCAAAGA<br>GACCAACGCCACCTACCCCAGTTCAGACGTTCCCTGTGATGCCACGTTGACTGAGAAAAGCTTTGAAACAGATATGAACCTAAACTTTCAAAACCTGTCAGTTATGGGACTCCGAATCCTCCTGCTGAAAGTAGC<br>CGGATTTAATCTGCTCATGACGCTGAGGCTGTGGTCCAGTTAACTCGAGTGTTTAAACCAGCCTCAAGAACACCCGAATG
p53-A2, TCRβ	ACTTGTTCTTTTTGCATCACAACCAGGCCTCCACAACCATGGCTACAAGGCTCCTCTGTTACACAGTACTTTGTCTCCTGGGTGCAAGAATTTTGAATTCAAAAGTCATTCAGACTCCAAGATATCTGGTGAAAGG<br>GCAAGGACAAAAAGCAAAGATGAGGTGTATCCCTGAAAAGGGACATCCGGTGGTCTTCTGGTACCAGCAGAACAAGAACAACGAGTTCAAGTTCTTGATTAACTTCCAGAATCAAGAAGTTCTTCAGCAAATAG<br>ACATGACTGAAAAACGATTCTCTGCTGAGTGTCCTTCAAACTCACCTTGCAGCCTAGAAATTCAGTCCTCTGAGGCAGGAGACTCAGCACTGTACCTCTGTGCCAGCAGTCTGTCAGGGGGCGGCACAGAAGTC<br>TTCTTTGGTAAAGGAACCAGACTCACAGTTGTAGAGGATCTGAGAAATGTGACTCCACCCAAGGTCTCCTTGTTTGAGCCATCAAAAGCAGAGATTGCAAACAAACAAAAGGCTACCCTCGTGTGCTTGGCCAG<br>GGGCTTCTTCCCTGACCACGTGGAGCTGAGCTGGTGGGTGAATGGCAAGGAGGTCCACAGTGGGGTCAGCACGGACCCTCAGGCCTACAAGGAGAGCAATTATAGCTACTGCCTGAGCAGCCGCCTGAGGG<br>TCTCTGCTACCTTCTGGCACAATCCTCGCAACCACTTCCGCTGCCAAGTGCAGTTCCATGGGCTTTCAGAGGAGGACAAGTGGCCAGAGGGCTCACCCAAACCTGTCACACAGAACATCAGTGCAGAGGCCTG<br>GGGCCGAGCAGACTGTGGGATTACCTCAGCATCCTATCAACAAGGGGTCTTGTCTGCCACCATCCTCTATGAGATCCTGCTAGGGAAAGCCACCCTGTATGCTGTGCTTGTCAGTACACTGGTGGTGATGGCT<br>ATGGTCAAAAGAAAGAATTCATAACTCGAGTGTTTAAACCAGCCTCAAGAACACCCGAATG
gp100-A2, TCRα	ACTTGTTCTTTTTGCATCACAACCAGGCCTCCACAACCATGGCATCCATTCGAGCTGTATTTATATTCCTGTGGCTGCAGCTGGACTTGGTGAATGGAGAGAATGTGGAGCAGCATCCTTCAACCCTGAGTGTCC<br>AGGAGGGAGACAGCGCTGTTATCAAGTGTACTTATTCAGACAGTGCCTCAAACTACTTCCCTTGGTATAAGCAAGAACTTGGAAAAGGACCTCAGCTTATTATAGACATTCGTTCAAATGTGGGCGAAAAGAAA<br>GACCAACGAATTGCTGTTACATTGAACAAGACAGCCAAACATTTCTCCCTGCACATCACAGAGACCCAACCTGAAGACTCGGCTGTCTACTTCTGTGCAGCAAGTACTTCGGGTGGTACTAGCTATGGAAAGCT<br>GACATTTGGACAAGGGACCATCTTGACTGTCCATCCAAATATCCAGAACCCTGACCCTGCCGTGTACCAGCTGAGAGACTCTAAATCCAGTGACAAGTCTGTCTGCCTATTCACCGATTTTGATTCTCAAACAAA<br>TGTGTCACAAAGTAAGGATTCTGATGTGTATATCACAGACAAAACTGTGCTAGACATGAGGTCTATGGACTTCAAGAGCAACAGTGCTGTGGCCTGGAGCAACAAATCCGACTTTGCATGTGCAAACGCCTTCA<br>ACAACAGCATTATTCCAGAAGACACCTTCTTCCCCAGCCCAGAAAGTTCCTGTGATGTCAAGCTGGTCGAGAAAAGCTTTGAAACAGATACGAACCTAAACTTTCAAAACCTGTCAGTGATTGGGTTCCGAATCC<br>TCCTCCTGAAAGTGGCCGGGTTTAATCTGCTCATGACGCTGCGGCTGTGGTCCAGCTAACTCGAGTGTTTAAACCAGCCTCAAGAACACCCGAATG
gp100-A2, TCRβ	ACTTGTTCTTTTTGCATCACAACCAGGCCTCCACAACCATGGGACCTCAGCTGCTGGGATACGTGGTGCTGTGTCTGCTTGGAGCCGGACCTCTGGAAGCCCAAGTGACACAGAACCCCAGATACCTGATCAC<br>CGTGACCGGCAAGAAACTGACCGTGACATGCAGCCAGAACATGAACCACGAGTACATGAGCTGGTACAGACAGGACCCTGGCCTGGGCCTGAGACAGATCTACTACAGCATGAACGTGGAAGTGACCGACAA<br>GGGCGACGTGCCCGAGGGCTACAAGGTGTCCAGAAAAGAGAAGCGGAACTTCCCACTGATCCTGGAAAGCCCATCTCCTAACCAGACCAGCCTGTACTTCTGCGCCAGCTCTCTGGGCAGCAGCTACGAGCA<br>GTATTTCGGCCCTGGCACCAGACTGACAGTGACCGAGGACCTGAAGAACGTGTTCCCACCTGAGGTGGCCGTGTTCGAGCCTTCTGAGGCCGAGATCAGCCACACACAGAAAGCCACACTCGTGTGTCTGGC<br>CACCGGCTTCTATCCCGATCACGTGGAACTGTCTTGGTGGGTCAACGGCAAAGAGGTGCACAGCGGCGTCAGCACAGATCCCCAGCCTCTGAAAGAACAGCCCGCTCTGAACGACAGCCGGTACTGTCTGAG<br>CAGCAGACTGAGAGTGTCCGCCACCTTCTGGCAGAACCCTCGGAACCACTTCAGATGCCAGGTGCAGTTCTACGGCCTGAGCGAGAACGATGAGTGGACCCAGGATAGAGCCAAGCCTGTGACTCAGATCGT<br>GTCTGCCGAAGCCTGGGGCAGAGCCGATTGTGGCTTTACCAGCGAGAGCTACCAGCAGGGCGTGCTGTCTGCCACAATCCTGTACGAGATCCTGCTGGGCAAAGCCACTCTGTACGCCGTGCTGGTTTCTGC<br>CCTGGTGCTGATGGCAATGGTCAAGCGGAAGGACAGCAGAGGCTAACTCGAGTGTTTAAACCAGCCTCAAGAACACCCGAATG
NY-ESO-1-A2, TCRα	ACTTGTTCTTTTTGCATCACAACCAGGCCTCCACAACCATGGAGACCCTGCTGGGCCTCCTTATACTGTGGCTTCAACTCCAATGGGTTAGCTCAAAGCAGGAAGTGACGCAAATTCCGGCAGCGTTGTCTGTTC<br>CAGAAGGTGAAAACCTTGTACTTAATTGTAGCTTCACAGACTCTGCGATTTACAATCTTCAGTGGTTTCGCCAAGACCCTGGGAAGGGTCTTACATCACTGCTCCTGATTCAATCTTCACAGCGCGAACAAACTA<br>GCGGGCGGCTTAACGCCTCACTTGATAAGAGCAGTGGCCGCTCCACTCTCTATATAGCGGCGTCTCAGCCGGGGGACTCTGCGACGTATTTGTGTGCTGTGCGACCACAAACTGGCGGGTCCTACATCCCAAC<br>ATTCGGAAGAGGCACCTCACTGATAGTACATCCAAATATACAGAATCCAGATCCCGCAGTATACCAACTTCGAGATAGTAAATCTAGTGACAAGTCCGTCTGTTTGTTTACAGATTTTGACTCCCAGACAAACGT

	CAGCCAGTCCAAGGACTCTGACGTCTATATCACAGACAAGACAGTGCTTGATATGCGGTCAATGGACTTCAAAAGCAACAGCGCCGTCGCATGGTCCAATAAGTCAGATTTCGCCTGTGCGAACGCGTTTAACA<br>ACTCCATAATCCCAGAGGATACCTTTTTCCCCAGTCCCGAGAGTTCATGTGACGTAAAGCTGGTAGAAAAAAGTTTCGAAACTGACACTAACCTCAACTTTCAAAATCTCAGTGTTATCGGCTTTCGGATTCTGTT<br>GTTGAAAGTGGCTGGATTCAATCTCCTGATGACACTTAGGCTGTGGAGTAGTTAACTCGAGTGTTTAAACCAGCCTCAAGAACACCCGAATG
NY-ESO-1-A2, TCRβ	ACTTGTTCTTTTTGCATCACAACCAGGCCTCCACAACCATGGCCACTACAATGGCGCCACGCCTCCTTTGCTGCGCTGCTTTGTCACTGCTTTGGGCAGGGCCGGTCAATGCCGGAGTAACCCAGACACCGAAA<br>TTCCAGGTGTTGAAAACCGGTCAGTCTATGACGTTGCAGTGCGCTCAAGATATGAACCATGAGTATATGTCCTGGTATAGGCAAGATCCAGGGATGGGTTTGCGACTCATCCATTATAGTGTGGGCGCCGGAAT<br>CACTGATCAGGGTGAGGTGCCGAACGGATATAACGTTAGTCGAAGTACGACGGAAGACTTTCCATTGAGGCTGCTTTCTGCGGCTCCTTCCCAGACGAGTGTGTACTTCTGCGCGTCATCTTATGTGGGGGCG<br>GCAGGGGAGCTGTTTTTCGGCGAAGGCTCACGGCTTACAGTGCTGGAAGACTTGAAAAATGTTTTTCCACCAGAAGTAGCGGTATTTGAACCGTCAGAGGCTGAAATCAGCCATACACAGAAGGCAACTCTGG<br>TTTGCTTGGCCACGGGCTTCTACCCTGATCACGTAGAGTTGTCCTGGTGGGTGAATGGAAAAGAAGTACACTCTGGTGTTTCCACCGACCCGCAACCGCTCAAGGAACAGCCAGCCCTTAACGATTCCCGGTAT<br>TGTCTGTCTTCAAGGCTGCGAGTATCCGCTACGTTCTGGCAAAATCCTCGAAATCACTTTCGGTGCCAAGTTCAATTCTATGGCCTTAGCGAGAATGACGAGTGGACTCAGGATCGAGCCAAACCCGTTACCCA<br>GATTGTCAGTGCCGAGGCTTGGGGTCGCGCTGACTGTGGTTTCACCTCTGAAAGCTACCAACAGGGTGTTCTTTCCGCTACAATACTGTATGAAATTCTCCTCGGCAAAGCGACGTTGTATGCGGTTTTGGTAT<br>CTGCCCTCGTTCTTATGGCAATGGTCAAGAGAAAAGATAGCCGAGGCTAACTCGAGTGTTTAAACCAGCCTCAAGAACACCCGAATG
MAGE-A3-DP4,<br>TCRα	ACTTGTTCTTTTTGCATCACAACCAGGCCTCCACCACCATGGCCTGTCCTGGATTTCTGTGGGCCCTCGTCATCTCTACCTGTCTGGAATTCAGCATGGCCCAGACCGTCACACAGAGCCAGCCTGAGATGTCTG<br>TGCAGGAGGCCGAGACAGTGACCCTGAGCTGCACCTACGATACAAGCGAGAGCGACTACTACCTGTTTTGGTACAAGCAGCCTCCTTCACGGCAGATGATCCTGGTCATCCGTCAAGAAGCCTATAAGCAGCA<br>GAATGCCACCGAGAACCGTTTCAGCGTGAATTTCCAGAAGGCCGCCAAGAGCTTCAGCCTGAAGATCAGCGATAGCCAGTTGGGAGATGCCGCTATGTATTTCTGTGCCCTGAGATCCTCCGGAACCTACAAG<br>TACATCTTCGGCACCGGTACCAGACTTAAGGTGCTGGCCAATATTCAGAACCCCGAGCCTGCCGTCTACCAGCTGAAGGATCCTAGAAGCCAGGATAGCACCCTGTGCCTGTTCACCGATTTCGACAGCCAGAT<br>TAACGTCCCCAAGACCATGGAATCCGGCACCTTCATCACAGATAAGACCGTTCTGGACATGAAAGCCATGGACTCCAAGAGCAATGGAGCCATTGCTTGGTCCAATCAAACCTCCTTCACCTGCCAGGATATCT<br>TCAAAGAGACAAACGCTACCTATCCTAGCTCCGATGTGCCCTGTGATGCCACCCTGACCGAGAAGTCCTTCGAGACAGATATGAACCTGAATTTCCAGAACCTGTCTGTCATGGGCCTGAGAATCCTGCTGCTG<br>AAGGTCGCCGGATTCAACCTGCTGATGACACTTAGACTGTGGTCCAGCTGACTCGAGTGTTTAAACCAGCCTCAAGAACACCCGAATG
MAGE-A3-DP4, TCRβ	AATACAAGCTACTTGTTCTTTTTGCATCACAACCAGGCCTCCACCACCATGGGAACCAGACTGCTGTTCTGGGTCGCCTTCTGTCTGCTGGGAGCCGATCATACAGGTGCTGGTGTCTCCCAGTCCCCCTCCAA<br>CAAGGTCACCGAGAAGGGCAAAGATGTGGAACTGCGATGTGACCCCATCAGCGGACACACAGCTCTGTACTGGTACAGACAGTCTCTCGGCCAGGGCCTCGAGTTCCTGATCTATTTCCAGGGAAACAGCGC<br>CCCTGACAAGTCAGGACTGCCTAGCGATAGATTCTCAGCTGAACGTACAGGAGGCTCCGTTTCCACACTGACCATCCAGAGAACCCAGCAAGAGGACTCTGCTGTGTACCTGTGTGCTTCTATCAGAACCGGC<br>CCTTTCTTCTCCGGCAATACAATCTACTTTGGCGAAGGCTCCTGGCTGACCGTGGTGGAAGATCTGAGAAATGTGACCCCACCTAAGGTTTCCCTGTTTGAACCTAGCAAGGCCGAGATCGCTAACAAGCAGAA<br>GGCTACCCTGGTCTGCCTGGCTAGAGGATTCTTTCCCGATCATGTTGAGCTGTCTTGGTGGGTCAACGGAAAGGAGGTCCACAGCGGAGTCTCTACAGATCCCCAGGCTTACAAAGAGTCCAACTACAGCTAC<br>TGCCTGAGCAGCAGACTGAGAGTGTCCGCCACCTTCTGGCACAACCCCAGAAACCATTTCAGATGCCAGGTGCAGTTCCACGGCCTGAGCGAAGAGGATAAGTGGCCTGAGGGATCTCCCAAGCCTGTGACA<br>CAAAATATCTCTGCAGAAGCTTGGGGAAGAGCTGATTGTGGAATCACCAGCGCCAGCTACCATCAGGGCGTTCTGTCCGCCACAATCCTGTATGAGATCCTTCTGGGCAAAGCTACTCTCTACGCTGTGCTGGT<br>GTCTGGCCTGGTGCTGATGGCCATGGTCAAGCGTAAGAACTCCTGACTCGAGTGTTTAAACCAGCCTCAAGAACACCCGAATG

Supplementary Figures

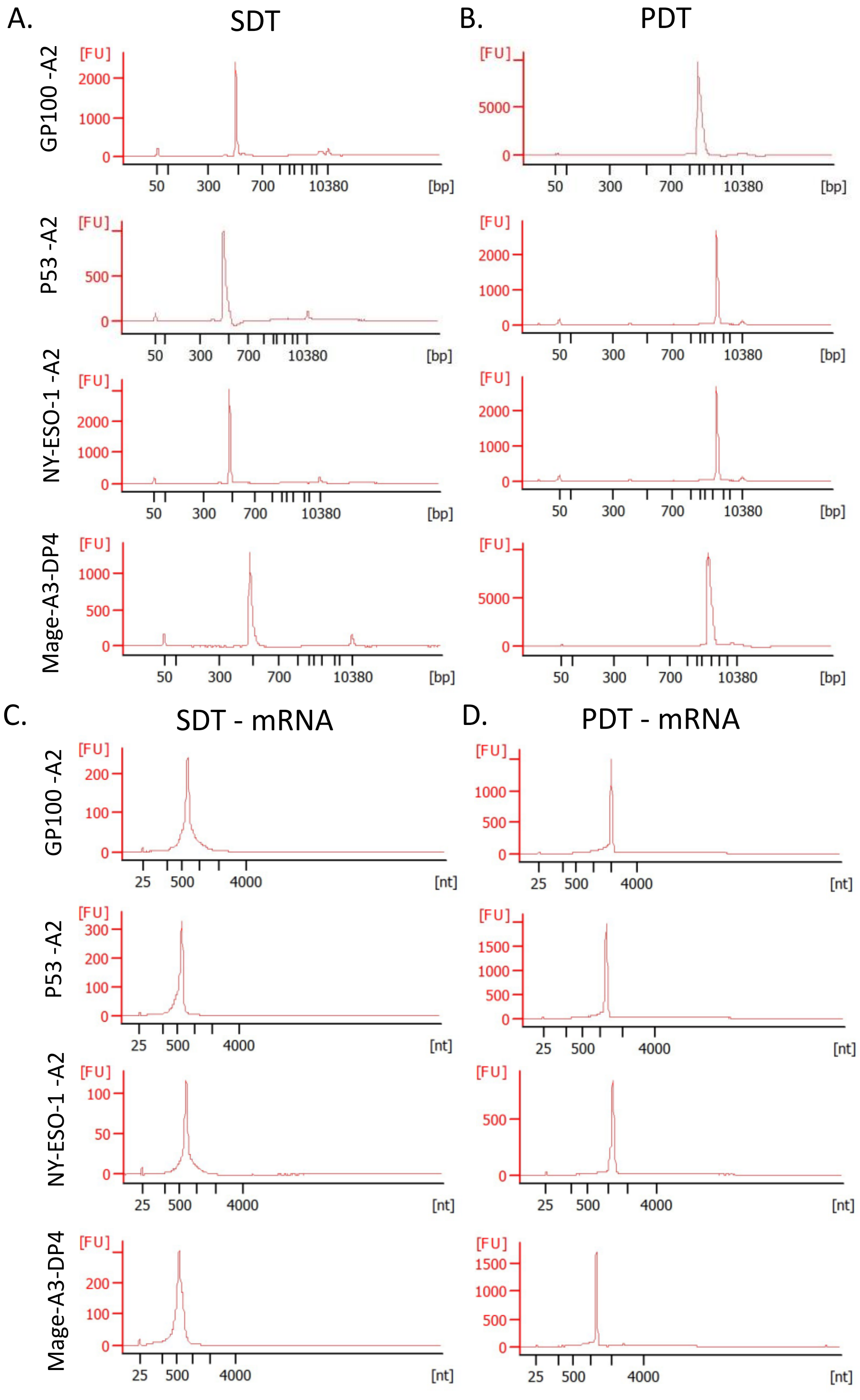

Figure S1: Representative capillary gel electropherograms. (A) SDT, (B) PDT, (C) SDT-mRNA and (D) PDT-mRNA encoding the HLA-A2-restricted epitopes of gp100 (YLEPGPVTA), NY-ESO-1 (SLLMWITQC) and p53 (LLGRNSFEV) and the HLA-DP4-restricted epitope of MAGE-A3 (TQHFVQENYLEY).

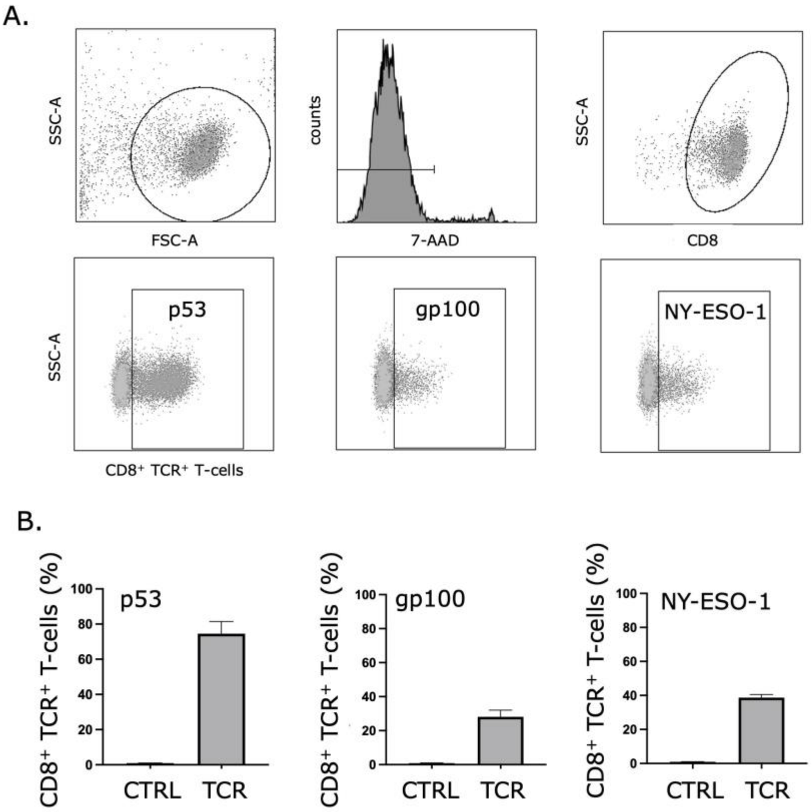

Figure S2: Expression of T-cell receptors on CD8⁺ T-cells after electroporation with mRNA encoding the T-cell receptor alpha and beta chain. (A) Representative flow cytometry graphs showing the gating strategy. We gated on viable cells based on forward (FSC-A) and side (SSC-A) scatter characteristics as well as absence of 7-AAD. We subsequently gated on cells that were bound by anti-CD8 antibodies and within this $CD8^+$ T-cell population we gated on cells that bound to dextramers specific for the introduced TCR. Three independent experiments were performed. (B) The graphs show the percentage of $CD8^+$ TCR^+ T-cells as detected in flow cytometry. The results are shown as mean ± standard error of the mean and summarize three independent experiments. Statistical analysis was not performed.

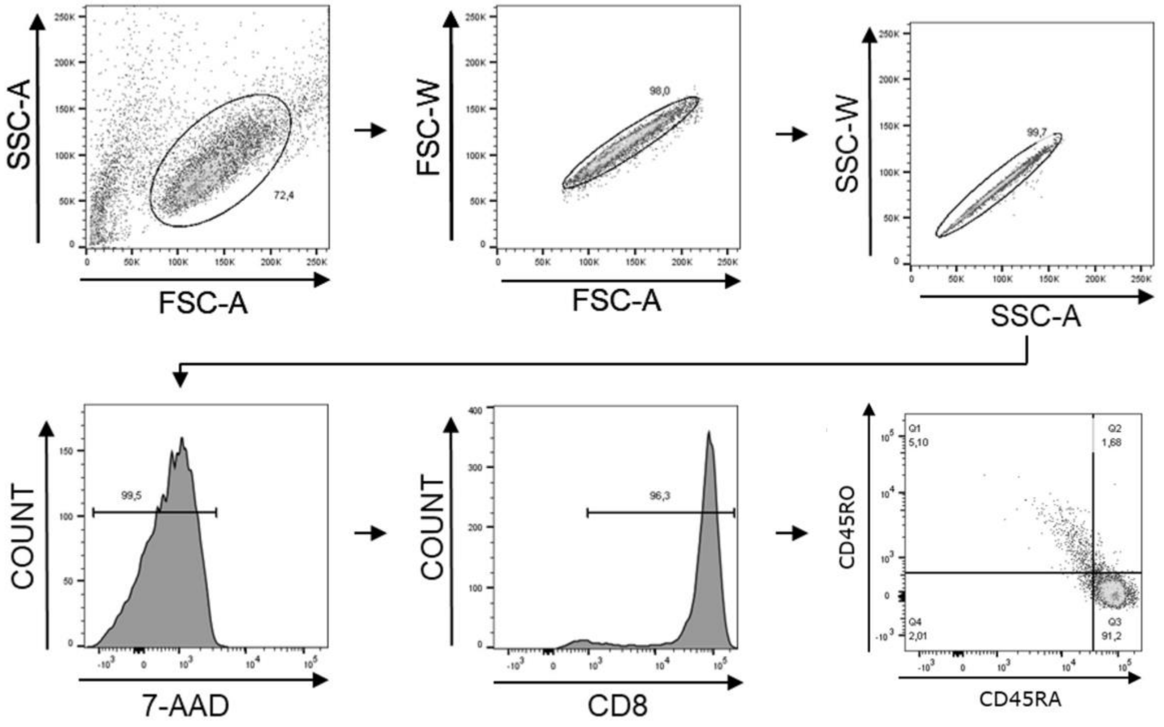

Figure S3: Selection of naïve (CD45RA⁺) CD8⁺ T-cells for neoantigen immunogenicity screening. Representative flow cytometry graphs showing the gating strategy. We gated on single viable cells based using forward (FSC-A/W) and side (SSC-A/W) scatter characteristics as well as absence of 7-AAD. We subsequently gated on cells that were bound by anti-CD8 antibodies and within this $CD8^+$ T-cell population we gated on cells that are $CD45RA^+$. Three independent experiments were performed.
