# Supplementary material for: A synthetic DNA template for fast manufacturing of versatile single epitope mRNA
Source: Mol Ther Nucleic Acids. 2022 Aug 17;29:943–54. doi: 10.1016/j.omtn.2022.08.021 (PMC9464653; doi:10.1016/j.omtn.2022.08.021)
Supplement: Document S2. Article plus supplemental information [file mmc2.pdf]

Molecular Therapy
Nucleic Acids
Original Article

A synthetic DNA template for fast manufacturing of versatile single epitope mRNA

Wout de Mey,[1] Phaedra De Schrijver,[1] Dorien Autaers,[1] Lena Pfitzer,[2] Bruno Fant,[2] Hanne Locy,[1] Arthur Esprit,[1] Lien Lybaert,[1,2] Cedric Bogaert,[2] Magali Verdonck,[1] Kris Thielemans,[1] Karine Breckpot,[1] and Lorenzo Franceschini[1]

[1]Laboratory for Molecular and Cellular Therapy, Department of Biomedical Sciences, Vrije Universiteit Brussel, Laarbeeklaan 103/E, 1090 Brussels, Belgium; [2]myNEO, Ottergemsesteenweg-Zuid 808, 9000 Ghent, Belgium

A flexible, affordable, and rapid vaccine platform is necessary to unlock the potential of personalized cancer vaccines in order to achieve full clinical efficiency. mRNA cancer vaccine manufacture relies on the rigid sequence design of multiepitope constructs produced by laborious bacterial cloning and time-consuming plasmid preparation. Here, we introduce a synthetic DNA template (SDT) assembly process, which allows cost- and time-efficient manufacturing of single (neo) epitope mRNA. We benchmarked SDT-derived mRNA against mRNA derived from a plasmid DNA template (PDT), showing that monocyte-derived dendritic cells (moDCs) electroporated with SDT-mRNA or PDT-mRNA, encoding HLA-I- or HLA-II-restricted (neo)epitopes, equally activated T cells that were modified to express the cognate T cell receptors. Furthermore, we validated the SDT-mRNA platform for neoepitope immunogenicity screening using the characterized HLA-A2-restricted neoepitope DHX40B and four new candidate HLA-A2-restricted melanoma neoepitopes. Finally, we compared SDT-mRNA with PDT-mRNA for vaccine development purposes. moDCs electroporated with mRNA encoding the HLA-A2-restricted, mutated Melan-A/Mart-1 epitope together with TriMix mRNA-generated high levels of functional Melan-A/Mart-1-specific CD8$^+$ T cells. In conclusion, SDT single epitope mRNA can be manufactured in a more flexible, cost-efficient, and time-efficient way compared with PDT-mRNA, allowing prompt neoepitope immunogenicity screening, and might be exploited for the development of personalized cancer vaccines.

INTRODUCTION

The encouraging results achieved in the last decade with immune-checkpoint inhibitors (ICIs) have put immunotherapy on the front line of unconventional cancer treatments.[1,2] However, studies have shown that only a subset of patients experience long-term clinical benefit from these new treatments.[3] Therefore, many research groups are devoted to identifying strategies that act in synergy with ICIs.[4] In this regard, cancer vaccination using tumor-specific neoantigens[5–7] able to induce, expand, and broaden the tumor-directed T cell repertoire have shown promising results.[8–10] Neoantigens are tumor-specific antigens, which originate from somatic mutations in the cancer cell genome and are not subjected to central tolerance.[11] Advances in next-generation sequencing (NGS) have enabled fast identification of non-synonymous mutations resulting in neoantigens and at the same time allowing human leukocyte antigen (HLA) allele genotyping. Neoantigen identification is based on *in silico* screening and prioritization using bioinformatics pipelines that score each candidate neoantigen on parameters such as allele class presentation, peptide processing by the proteasome, T cell receptor (TCR) binding, major histocompatibility complex (MHC) affinity, peptide-MHC (pMHC) stability, and tumor neoantigen source.[12,13]

The main challenge is to identify, among the *in silico* predicted neoantigens, those able to trigger a robust immune response in the patient, as this could potentially result in the eradication of tumor cells presenting the neoantigens.[14–16] Among the *in silico* identified neoantigen candidates, only a small number renders peptides (neoepitopes) that can be presented on MHC molecules on the cell surface, and only few of these pMHC complexes might be immunogenic, therefore eliciting a T cell response. For this reason, experimental validation of neoepitope immunogenicity is extremely important.[17,18] Moreover, neoantigens are unique for each cancer patient, demanding a flexible and fast manufacturing platform to facilitate both the immunogenicity screening and subsequent personalized vaccine production. In this regard, mRNA is an attractive tool.[19] Once in the cell, mRNA is translated into a polypeptide that contains the neoepitope sequence, which is further processed and degraded by the proteasome. Peptides released from proteasome degradation are transported to the endoplasmic reticulum and loaded onto MHC molecules, forming pMHC complexes. These pMHC complexes are transferred to the cell surface for presentation to the TCR of T cells.[20]

mRNA is transcribed from a DNA template,[21] which contains the protein sequence aligned between a start and stop codon, indicated as the open reading frame (ORF). Besides the protein sequence, the template also contains untranslated sequences. These include the T7

Received 4 May 2022; accepted 14 August 2022;
https://doi.org/10.1016/j.omtn.2022.08.021.

Correspondence: Lorenzo Franceschini, Laboratory for Molecular and Cellular Therapy, Department of Biomedical Sciences, Vrije Universiteit Brussel, Laarbeeklaan 103/E, 1090 Brussels, Belgium.
E-mail: lorenzo.franceschini@vub.be

This is an open access article under the CC BY license (http://creativecommons.org/licenses/by/4.0/).

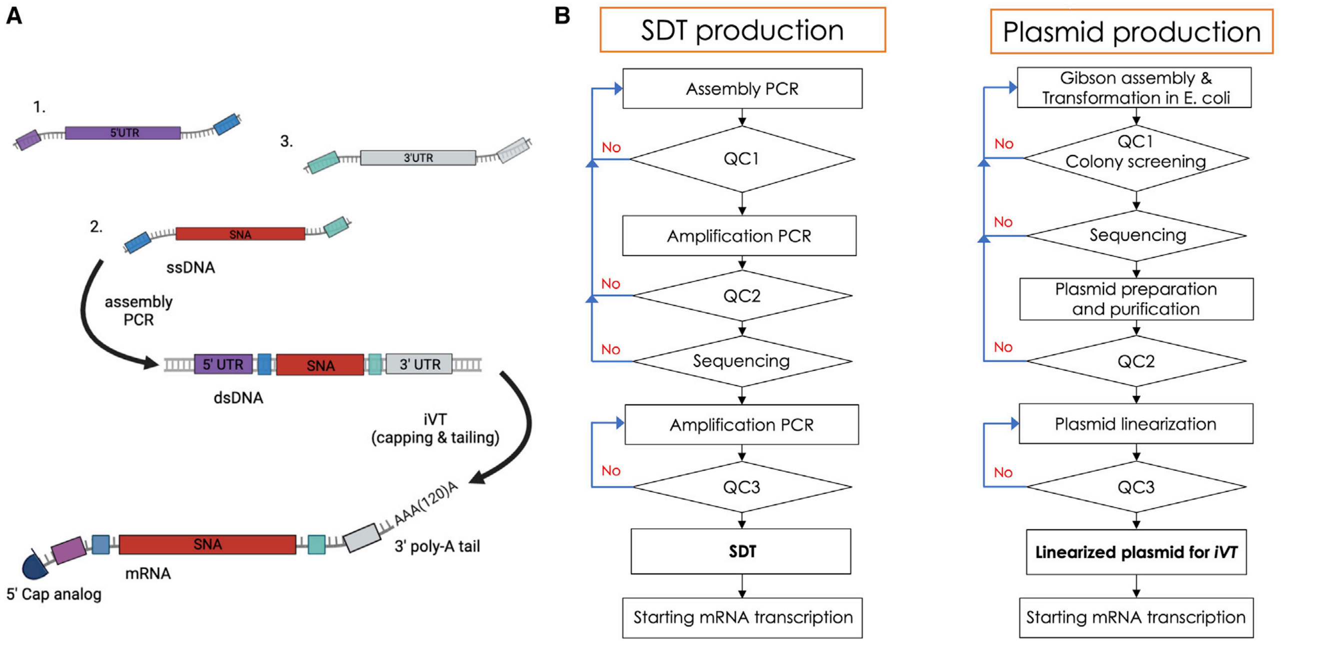

Figure 1. An SDT for *in vitro* synthesis of epitope encoding mRNA

(A) Schematic representation of the SDT assembly reaction and mRNA synthesis: the SDT is formed by hybridization of three ssDNA molecules by aPCR: oligo 1 (purple) contains the T7 promoter, Kozak, and 5′ UTR; oligo 2 (red) encodes the cDNA to the mRNA of interest; and oligo 3 (gray) contains the 3′ UTR and a short poly(A) sequence. The resulting SDT is further amplified by PCR and used as template for *in vitro* transcription of mRNA. The mRNA is capped, and a poly(A) tail of ~150 consecutive adenosine monophosphates is added during the enzymatic reaction. (B) Schematic representation of the workflow for mRNA production using an SDT (left) or a PDT (right). The DNA template and resulting mRNA are subjected to thorough quality controls (QC).

promoter sequence, a binding site for the T7 polymerase to initiate the mRNA transcription enzymatic reaction from the DNA template,[22] a Kozak sequence for the eukaryotic translation initiation,[23,24] untranslated regions (UTRs) at the 5′ and 3′ end of the ORF sequence,[25–28] and a poly(A) tail at the 3′ end.[29] UTRs and the poly(A) tail are key for mRNA stability and translatability, and as a result of enhanced biological activity. *In vitro* transcription (*iVT*) of mRNA requires a DNA template, for which often a plasmid DNA is used, produced by microbial fermentation, here indicated as plasmid DNA template (PDT).[30,31] However, cloning and further preparation of PDT is a money-consuming, multistep process that takes from several days even up to weeks, involving the use of bacteria and antibiotics, without a guarantee that the correct bacterial clone will be identified and isolated for a long time.[32] Moreover, the use of genetically modified antibiotic-resistant bacteria and the risk of biocontamination in the final product are serious concerns for the good manufacturing practice (GMP) development of mRNA-based vaccines.[33] Here we introduce a novel, straightforward, and flexible approach for cost- and time-efficient generation of mRNA encoding single-antigen-derived peptides (epitopes). This approach is based on a synthetic DNA template (SDT) generated by assembly polymerase chain reaction (aPCR) using synthetic oligonucleotides as starting material. The resulting SDT is sequence verified and suited for *iVT* of mRNA. In the present work we showed that SDT-mRNA, similarly to PDT-mRNA, is presented to T cells after electroporation into monocyte-derived dendritic cells (moDCs) and that SDT-mRNA can be used to screen the immunogenicity of neoepitopes as well as develop cancer vaccines.

RESULTS

Synthetic DNA template for *in vitro* mRNA transcription

Three synthetic single-stranded DNA (ssDNA) molecules (<200 bases) were designed to hybridize together via aPCR, resulting in a synthetic double-stranded DNA (dsDNA) template that can be used for *iVT* of mRNA (Figure 1A). The SDT contains the sequence for the T7 promoter,[22] Kozak sequence,[23,24] and leader sequence from the human β-globin gene as 5′ UTR,[25,26] a translation starting and stopping codon ATG/TGA respectively framing the ORF sequence of interest and the 3′ UTR derived from the goat β-globin protein.[27] The ORF sequence encodes a 27-mer peptide, consisting of the immunodominant 9-mer epitope of interest. The ORF covers also the HLA-II sorting signal of dendritic cell (DC) lysosome-associated membrane protein (DC-LAMP, CD208) as this was shown to enhance presentation of HLA-I-restricted epitopes and to enable presentation of HLA-II-restricted epitopes.[34] The resulting SDT can be amplified up to several micrograms by PCR. In one extensive enzymatic reaction and only in a few hours, it is possible to shift from DNA production to mRNA synthesis. Samples from each intermediate product can be sent for sequencing analysis and capillary gel electrophoresis (quality controls) (Figure 1B). Transcribed

Table 1. Release criteria of the synthetic DNA template versus plasmid DNA template

			DNA							
			gp100-A2		p53-A2		NY-ESO1-A2		MAGE-A3-DP4	
Test	Method	Release criteria	SDT	PDT	SDT	PDT	SDT	PDT	SDT	PDT
Appearance	visual inspection	clear, colorless solution	V	V	V	V	V	V	V	V
Content	spectrophotometry	>0.1 (SDT) or 1 mg/mL ± 10% (PDT)	0.138 mg/mL	0.929 mg/mL	0.144 mg/mL	0.944 mg/mL	0.138 mg/mL	0.940 mg/mL	0.156 mg/mL	1.053 mg/mL
$A_{260/280}$	spectrophotometry	1.8–2.0	1.9	1.8	1.9	1.8	1.9	1.8	1.9	1.8
Integrity	CGE-DNA	one peak matching the theoretical DNA length	S1.A	S1.B	S1.A	S1.B	S1.A	S1.B	S1.A	S1.B
Identity	DNA sequencing	100% alignment with the reference sequence	100%	100%	100%	100%	100%	100%	100%	100%

V, verified.
$A_{260/280}$, ratio of the absorbance at 260 versus 280 nm; A2, restricted to HLA-A2; CGE, capillary gel electrophoresis; DP4, restricted to HLA-DP4.

mRNA is capped, and an extensive poly(A) tail is added during the *iVT* reaction.[21] We compared the manufacture of SDT-mRNA (Table S1) and PDT-mRNA (using the pLMCT plasmid, Table S2) encoding the published HLA-DP4-restricted epitope of the shared antigen MAGE-A3 (TQHFVQENYLEY)[34,35] and the published HLA-A2-restricted epitopes of the shared antigens gp100 (YLEPGPVTA),[36–39] NY-ESO-1 (SLLMWITQC),[40–42] and p53 (LLGRNSFEV),[43,44] and, moreover, the published mutated antigen Melan-A/Mart-1 (A27L) (ELAGIGILTV).[45] We subjected the resulting DNA templates and mRNA to thorough quality controls, including visual inspection (clear, colorless solution), spectrophotometry readout (yield, purity [$A_{260/280}$]), capillary gel electrophoresis (integrity), and sequence analysis (alignment with reference sequence). According to the quality control specifications, we observed that SDT and SDT-mRNA were of quality comparable with that of PDT and PDT-mRNA, respectively, for these parameters (Tables 1 and 2; Figure S1).

Electroporation of dendritic cells with mRNA transcribed from a synthetic DNA template or a plasmid DNA template does not induce phenotypic maturation

Electroporation has been extensively used to deliver mRNA into the cytosol of moDCs,[46–48] thereby bypassing endosomes. As a result, pattern recognition receptors (PRRs) that could potentially detect mRNA, including Toll-like receptor 7 (TLR7) and TLR8,[49] or that could potentially detect dsRNA impurities, including TLR3,[50] are bypassed. However, several cytosolic PRRs, such as retinoic acid-inducible gene I (RIG-I), which could also sense dsRNA impurities, are not bypassed.[51] Consequently, phenotypic maturation of moDCs after mRNA electroporation can be considered a result of sensing dsRNA impurities in the mRNA preparation. We electroporated moDCs with SDT-mRNA or PDT-mRNA and evaluated the moDC viability and phenotype at 6 h and 24 h after mRNA delivery. We did not observe changes in moDC viability after electroporation with SDT-mRNA or PDT-mRNA (Figure 2A). We further did not observe significant changes in the percentage of moDCs that expressed HLA-A2, HLA-DR, CD40, or CCR7 after electroporation with SDT-mRNA or PDT-mRNA, although we observed that the percentage of moDCs that expressed CD86 was significantly increased after electroporation with both SDT-mRNA and PDT-mRNA (Figure 2B).

Enhanced presentation of (neo)epitopes to T cells by dendritic cells electroporated with (neo)epitope mRNA manufactured from a plasmid DNA template or synthetic DNA template

The faster, cheaper, and more flexible SDT-mRNA manufacturing platform could have far-reaching potential in vaccine development as a means to screen neoepitope immunogenicity. A prerequisite to unlock this potential is the presentation of the encoded neoepitope by SDT-mRNA electroporated DCs to T cells. Both $CD4^+$ T helper 1 cells and $CD8^+$ cytotoxic T cells are required to mount a potent antitumor immune response.[52–54] However, mRNA that is transfected in DCs is translated into peptides that are processed for presentation onto HLA-I molecules and, hence, presentation to $CD8^+$ T cells.[20,55] Obtaining presentation of HLA-II-restricted epitopes, while simultaneously increasing the presentation of HLA-I-restricted epitopes upon delivery of mRNA-encoded antigens to moDCs, was shown to be possible by addition of the HLA-II sorting signal of DC-LAMP to the antigenic sequence.[34] Therefore, this sorting signal was fused to the cDNA encoding a 27-mer peptide, consisting of the immunodominant 9-mer epitope under evaluation, which is flanked at the 5′ end and 3′ end by additional protein 9-mers. We evaluated presentation of the HLA-DP4-restricted epitope of the shared antigen MAGE-A3 (TQHFVQENYLEY)[34,35] and the HLA-A2-restricted epitopes of the shared antigens gp100 (YLEPGPVTA),[36–39] NY-ESO-1 (SLLMWITQC),[40–42] and p53 (LLGRNSFEV).[43,44]

We electroporated $HLA\text{-}A2^+$ moDCs with SDT-mRNA or PDT-mRNA and co-cultured them at a 1:1 ratio with $CD8^+$ T cells that were electroporated with mRNA encoding the corresponding TCRα and TCRβ chain to test antigen presentation to $CD8^+$ T cells (Table S3). Prior to the culture, we verified TCR expression on $CD8^+$ T cells by flow cytometry (Figure S3). We quantified the amount of interferon-γ (IFN-γ) that was produced by the T cells during the 24-h co-culture in ELISA as a measure of antigen presentation

Table 2. Release criteria of the mRNA manufactured using the synthetic DNA template versus plasmid DNA template

			RNA							
			gp100-A2		p53-A2		NY-ESO1-A2		MAGE-A3-DP4	
Test	Method	Release criteria	SDT	PDT	SDT	PDT	SDT	PDT	SDT	PDT
Appearance	visual inspection	clear, colorless solution	V	V	V	V	V	V	V	V
Content	spectrophotometry	1 mg/mL ± 10%	1.050 mg/mL	0.997 mg/mL	1,043 mg/mL	1.009 mg/mL	1.080 mg/mL	0.916 mg/mL	1.086 mg/mL	1.053 mg/mL
$A_{260/280}$	spectrophotometry	>2.0	2.54	2.029	2.25	2.105	2.25	2.073	2.38	2.089
Integrity	CGE-RNA	one peak matching the theoretical RNA length	S1.C	S1.D	S1.C	S1.D	S1.C	S1.D	S1.C	S1.D
Identity	RNase digestion	no detectable RNA after RNase digestion on CGE	V	V	V	V	V	V	V	V
	cDNA sequencing	epitope identification	V	V	V	V	V	V	V	V
Tailing efficiency	CGE-RNA	SDT: peak shift from the theoretical DNA length	S1.A/S1.C		S1.A/S1.C		S1.A/S1.C		S1.A/S1.C	
		PDT: peak size matching theoretical RNA length		S1.D		S1.D		S1.D		S1.D
5′ Capping validation	ELISA	$CD8^+$ TCR^+ T cell activation in response to moDCs electroporated with 5′ capped and non-capped RNA	V	V	V	V	V	V	V	V

V, verified.

$A_{260/280}$, ratio of the absorbance at 260 versus 280 nm; A2, restricted to HLA-A2; CGE, capillary gel electrophoresis; DP4, restricted to HLA-DP4; cDNA, copy DNA.

by moDCs to the $CD8^+$ T cells. As a control, we set up identical co-cultures with T cells that were not modified. We only observed production of IFN-γ when antigen-presenting moDCs were co-cultured with $CD8^+$ T cells modified to express the corresponding TCR without statistically significant differences between antigen presentation by moDCs electroporated with SDT-mRNA or PDT-mRNA (Figure 3A).

We established similar co-cultures to study presentation of the HLA-DP4-restricted MAGE-A3 epitope by SDT-mRNA or PDT-mRNA electroporated moDCs to $CD4^+$ T cells that were electroporated with mRNA encoding the corresponding TCRα and TCRβ chain.[35] As a measure of antigen presentation by moDCs to these $CD4^+$ T cells, we quantified IFN-γ produced by the T cells during the 24-h co-culture using ELISA and further analyzed the expression of the T cell activation markers CD69 and OX40 using flow cytometry (Figure 3B). To confirm the necessity of the HLA-II sorting signal of DC-LAMP to achieve antigen presentation in HLA-II, we used moDCs electroporated with PDT-mRNA encoding the MAGE-A3 antigen without its fusion to the DC-LAMP sorting signal. We observed little T cell activation when moDCs were electroporated with PDT-mRNA encoding for the non-HLA-II-targeted MAGE-A3, while similar levels of T cell activation were obtained in co-cultures with moDCs electroporated with SDT-mRNA or PDT-mRNA encoding the HLA-II-targeted MAGE-A3, signifying comparable levels of antigen presentation only when the epitope is actively shuttled to HLA-II compartments (Figure 3B).

mRNA manufactured from synthetic DNA template might be used for personalized vaccine development

As we established that SDT-mRNA can be used to convert moDCs into (neo)epitope presenting cells, we next evaluated whether the SDT-mRNA could be used as an active ingredient for personalized vaccine development. Therefore, we generated HLA-$A2^+$ moDCs and electroporated them with PDT-mRNA encoding the proprietary TriMix to endow the moDCs with strong T cell stimulatory capacity.[56] We co-electroporated these moDCs with SDT-mRNA or PDT-mRNA encoding the mutated, highly immunogenic, HLA-A2-restricted Melan-A/Mart-1 (A27L) epitope[57] and co-cultured them at a 1:2 ratio with autologous, naive ($CD45RA^+$) $CD8^+$ T cells for 10 days (Figure S3), according to the protocol described by Ali et al.[58] We evaluated the percentage of activated $CD8^+$ T cells in flow cytometry and their ability to produce IFN-γ using enzyme-linked immunospot assay (ELISPOT), measured by spot-forming units (SFU) per 10^5 $CD8^+$ T cells. We observed that HLA-$A2^+$ moDCs presenting the Melan-A/Mart-1 (A27L) epitope after electroporation with SDT-mRNA or PDT-mRNA stimulated similarly high levels of functional Melan-A/Mart-1 (A27L)-specific $CD8^+$ T cells (Figures 4A and 4B).

Screening neoepitope immunogenicity using mRNA produced from a synthetic DNA template

Although advances in NGS techniques and computational pipelines have made *in silico* neoantigen prediction possible,[19] each proposed candidate neoantigen should be experimentally validated to confirm its immunogenicity, i.e., its ability to trigger T cell

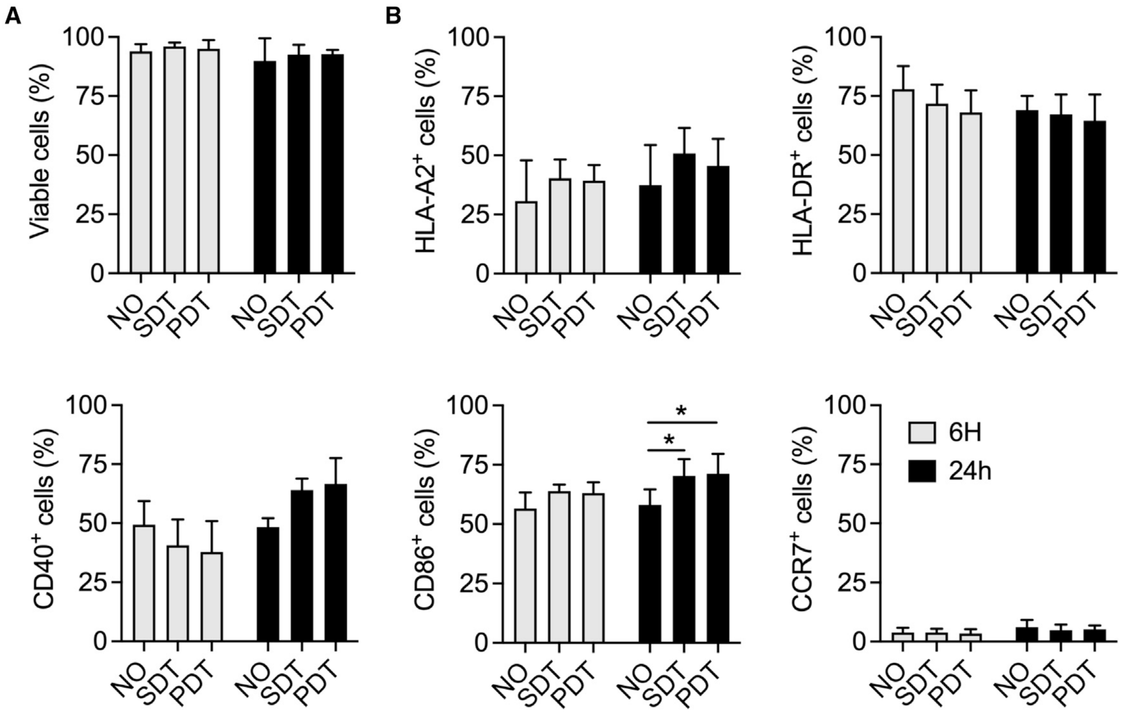

Figure 2. Viability and phenotype of dendritic cells electroporated with mRNA produced using a synthetic DNA template or a plasmid DNA template
(A) Bar graph showing the viability of non-electroporated moDCs (NO) or moDCs electroporated with SDT-mRNA (SDT) or PDT-mRNA (PDT) expressed as percentage of viable cells. (B) Bar graphs showing the percentage of cells that express HLA-A2, HLA-DR, CD40, CD86, or CCR7 within the conditions: non-electroporated moDCs (NO) or moDCs electroporated with SDT-mRNA (SDT) or PDT-mRNA (PDT). Results are shown as mean ± standard error of the mean and summarize three independent experiments. One-way ANOVA with Bonferroni's correction was performed to determine statistical significance, shown as $*p < 0.05$.

activation.[15,16,59] As SDT-mRNA encoding single epitopes can be manufactured in a time- and cost-reductive way and as electroporation of moDCs with single epitope encoding SDT-mRNA and TriMix mRNA results in antigen presentation and *de novo* stimulation of epitope-specific T cells, we used a similar setup to validate SDT-mRNA as a tool to screen the immunogenicity of neoepitopes. Using the proprietary ImmunoEngine bioinformatics pipeline developed by myNEO, 71 non-synonymous expressed somatic coding mutations were identified by whole-genome sequencing of a melanoma biopsy. Four of these neoantigens (ATM(H448L), KIF13A(F539I), PLCG1(L244F), ZMYM3(R1256C)) are predicted to be presented in HLA-A2 and were screened for their immunogenicity using moDCs from three HLA-A2$^+$ donors and autologous naive (CD45RA$^+$) CD8$^+$ T cells (Table S1). An epitope that was previously described to be immunogenic (DHX40B) was used as a positive control.[60] We observed a T cell response against DHX40B in all HLA-A2$^+$ donors after two rounds of stimulation, confirming its immunogenicity and the validity of this screening approach using SDT-mRNA (Figure 5A). The neoantigen ATM(H448L) did not generate a T cell response in any of the donors, and we therefore considered this neoantigen non-immunogenic in this study. The neoantigens indicated as KIF13A(F539I), PLCG1(L244F), and ZMYM3(R1256C) generated a T cell response in one out of three, two out of three, and three out of three donors, respectively (Figure 5B).

DISCUSSION

Fast validation of immunogenic neoantigen candidates expressed and presented by tumor cells and, consequently, a prompt vaccine-manufacturing pipeline is necessary to develop personalized cancer vaccines.[14–16] Identification of immunogenic neoepitopes is a major obstacle to the translation of neoantigen-based cancer immunotherapy into clinical studies.[61–63] For *in vitro* studies to identify cognate TCRs and for the manufacture of neoantigen mRNA therapeutic vaccines, most often a cloning strategy is used in which a string of neoantigens are inserted into a plasmid. Also, personalized DNA-based vaccines or viral vectors most often encode a polyepitope construct.[31,64–66] Several groups have been screening neoantigens through polyepitope constructs, using healthy donor HLA-matched peripheral blood mononuclear cells (PBMCs).[58,67,68] Depending on the observed immunogenicity for each prioritized neoantigen, the polyepitope construct might undergo a design revision, resulting in a second round of cloning, which might delay this already laborious and time-consuming procedure. Furthermore, the design of the polyepitope construct could impact on epitope immunogenicity readout through effects introduced by the adjoining sequences within the polyepitope construct. Spacer

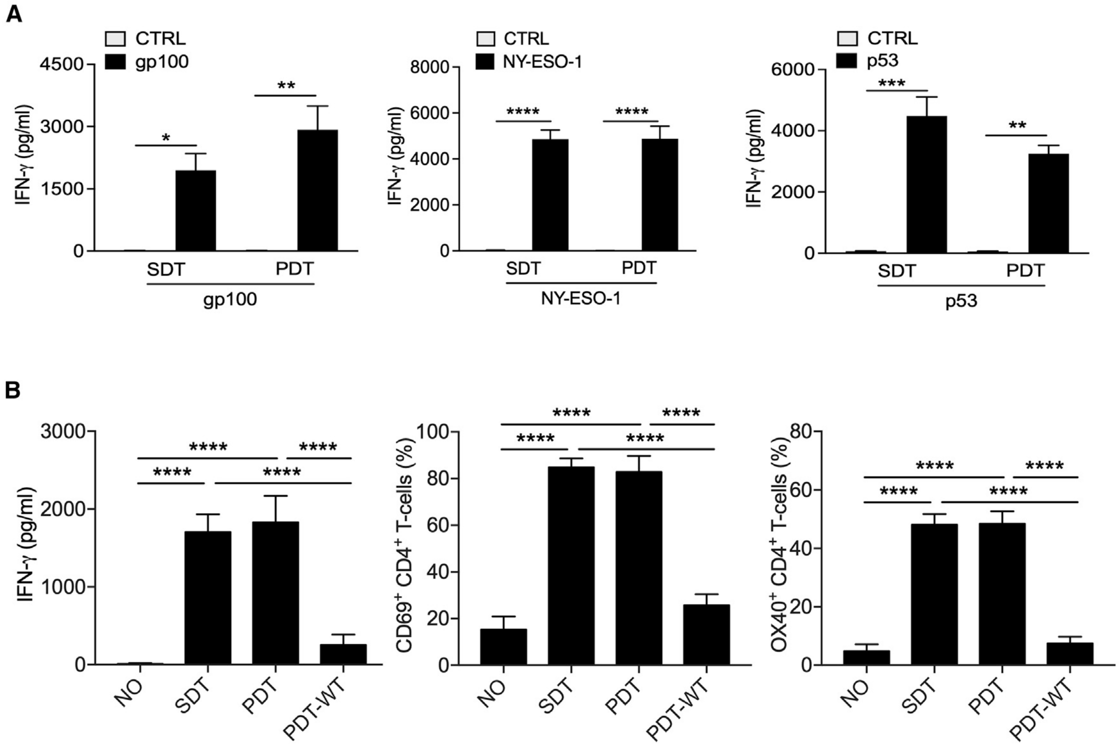

Figure 3. Antigen presentation by dendritic cells electroporated with epitope mRNA produced using a synthetic DNA template or a plasmid DNA template is similar

(A) Bar graphs showing production of IFN-γ, quantified by ELISA, by $CD8^+$ TCR^+ T cells in response to antigen presentation by moDCs that were electroporated with SDT-mRNA or PDT-mRNA encoding the HLA-A2-restricted epitope of gp100 (left), NY-ESO-1 (middle), or p53 (right). The data are representative of results obtained in three independent experiments using cells of two different donors. (B) Bar graphs showing production of IFN-γ, quantified by ELISA (left), or the percentage of cells expressing the activation marker CD69 (middle) or OX40 (right), quantified in flow cytometry within $CD4^+$ TCR^+ T cells in response to antigen presentation by moDCs that were electroporated with SDT-mRNA or PDT-mRNA encoding the HLA-DP4-restricted and HLA-II-targeted epitope of MAGE-A3. As controls, T cells were co-cultured with non-electroporated moDCs (NO) or moDCs electroporated with mRNA encoding the non-HLA-II-targeted MAGE-A3 epitope (PDT-WT). The data are representative of results obtained in three independent experiments using cells of one donor. The results shown in (A) and (B) are summarized as mean ± standard error of the mean. One-way ANOVA with Bonferroni's correction was performed to determine statistical significance, shown as follows: $^{*}p < 0.05$, $^{**}p < 0.01$, $^{***}p < 0.001$, $^{****}p < 0.0001$.

sequences, in particular, which are commonly placed between each epitope, could lead to non-specific immunogenic effects. Moreover, the order of the epitopes within a polyepitope construct might also play a role in the observed immunogenicity and could induce competition within the polyepitope molecule owing to the prioritization based on epitope position.[58,69,70] This might negatively affect the immunogenicity of screened neoantigens. Therefore, we developed a straightforward, cost- and time-reductive workflow to produce *in vitro* transcribed mRNA encoding for single (neo)epitopes starting from an SDT. The sequence design of the SDT-mRNA-encoded (neo)epitope includes a sorting signal of DC-LAMP, as this was shown to increase presentation of HLA-I-restricted peptides and as this was shown to be key to obtaining presentation of HLA-II-restricted peptides after their mRNA-mediated delivery,[34,71] as confirmed in this study. Several studies have transcription of RNA from a plasmid-derived or synthetic PCR template.[72,73] In this work, we used high-quality PAGE-purified oligonucleotides, within a price range similar to that of the gene fragments required for cloning of the control plasmids. Nevertheless, the manufacturing cost remains low because of the reduced operator working hours and the limited rental of facilities and necessary equipment linked herewith, owing to the quick SDT preparation process. The resulting SDT-mRNA is of quality comparable with that of the PDT-mRNA, as shown in the thorough quality control that evaluated yield, integrity, and purity. Moreover, the SDT-mRNA platform offers the advantage of fast, cost-efficient manufacturing of neoantigen mRNA without any risk of bioburden resulting from possible contamination with bacterial product traces or antibiotics. We showed that the SDT-mRNA and PDT-mRNA produced in this study and electroporated into moDCs did not impact on their cell viability and had little effect on their phenotype, with the exception of a small, though statistically significant, increase in CD86 expression. The latter might be a result of sensing of some dsRNA impurities by cytosolic dsRNA sensors,[51] although this is of little concern given the proven antigen presentation by moDCs, which was comparable after electroporation with SDT-mRNA versus PDT-mRNA. As a result, the first prerequisite for use of SDT-mRNA for neoantigen immunogenicity screening and vaccine

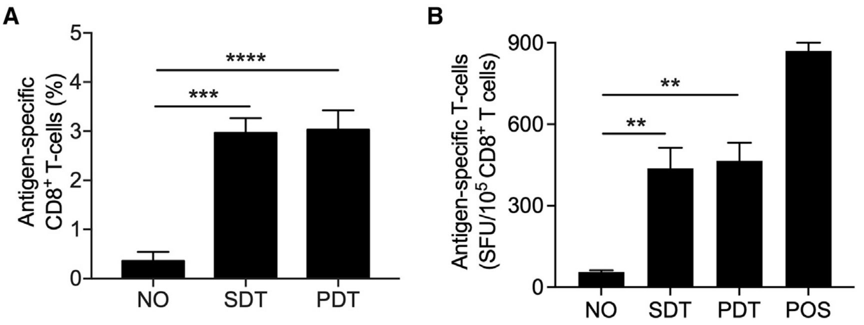

Figure 4. mRNA manufactured from a synthetic DNA template can be used for vaccine development

(A) Bar graph showing the percentage of Melan-A/Mart-1 (A27L)-specific $CD8^+$ T cells, quantified in flow cytometry, obtained after 10 days of co-culture with an autologous, HLA-A2$^+$ moDC vaccine, consisting of moDCs co-electroporated with PDT-mRNA encoding TriMix, and PDT-mRNA of SDT-mRNA encoding the Melan-A/Mart-1 (A27L) epitope (fused to the sorting signal of DC-LAMP). As a control, $CD8^+$ T cells were co-cultured with moDCs that were not modified to present the Melan-A/Mart-1 (A27L) epitope (NO). (B) Bar graph summarizing the number of SFU per 10^5 $CD8^+$ T cells, quantified using ELISPOT, after overnight restimulation of the activated $CD8^+$ T cells with HLA-A2$^+$ K562 cells presenting the Melan-A/Mart-1 (A27L) epitope. As a technical control, $CD8^+$ T cells were activated with anti-CD3/CD28 antibody-coated beads (POS). The results in (A) are summarized as mean ± standard error of the mean of three independent experiments performed with cells of three different donors. The results in (B) are summarized as mean ± standard error of the mean of two independent experiments performed with cells of two different donors. One-way ANOVA with Bonferroni's correction was performed to determine statistical significance, shown as follows: $^{**}p < 0.01$, $^{***}p < 0.001$, $^{****}p < 0.0001$.

development is fulfilled. We showed that SDT-mRNA can indeed be used for both purposes using known mutated antigens, including DHX40 and Melan-A/Mart-1 (A27L), and four HLA-A2-restricted candidate neoantigens that were identified in melanoma tumor sample using NGS and the ImmunoEngine pipeline developed by myNEO. Therefore, we provided results showing that the SDT-mRNA platform is a cost- and time-reductive approach to generate neoepitope mRNA libraries that can be used to screen the immunogenicity of neoantigens and, moreover, that this might be exploited in the future to produce personalized mRNA batches suitable for a single patient dose in a very short time.

MATERIALS AND METHODS

Cell lines

The K562 cell line was purchased from the American Type Culture Collection. K562 cells were cultured in Iscove's modified Dulbecco's medium (IMDM) supplemented with 10% fetal bovine serum, 2 mM L-glutamine, 100 U/mL penicillin, and 100 μg/mL streptomycin, and maintained at 37°C in 5% CO_2. The cell line was tested for the absence of mycoplasma contamination by PCR.

Generation of monocyte-derived dendritic cells

Generation of moDCs was performed according to GMP. On day 0, a leukapheresis was performed on healthy donors at the Hematology Unit of the University Hospital in Brussels (UZ Brussel, Belgium) using an apheresis device (Spectra Optia apheresis system; Terumo BCT) to collect the PBMC fraction. This study was approved by the Ethical Committee of the UZ Brussel (2013/198). The leukapheresis product was further processed at the DC manufacturing unit of the Laboratory for Molecular and Cellular Therapy at the Vrije Universiteit Brussel (LMCT-VUB, Brussels, Belgium). An elutriation procedure (Elutra Cell Separation System; Terumo BCT) was performed to enrich monocytes. These monocytes were cultured in a cell culture bag with GMP-grade DC medium (CellGenix), supplemented with GMP-grade human serum albumin (1%; CAF-DCF) and GMP-grade cytokines: 500 IU/mL recombinant interleukin-4 (IL-4) (CellGenix) and 1,000 IU/mL recombinant granulocyte macrophage colony-stimulating factor (Leukine; Sanofi). Differentiation from monocyte to moDCs was allowed for 60–72 h by incubating the cells at 37°C and 5% CO_2. The differentiated moDCs were harvested and, after count and viability assessment, cryopreserved in 5% DMSO cryopreservation medium (CryoStor CS5; BioLife Solutions). Cryovials were immediately transferred into a freezing container (CoolCell; Corning) and placed at −80°C. After overnight incubation at −80°C, vials were stored in the vapor phase of a liquid nitrogen container. From the same leukapheresis material, we stored the monocyte-depleted fraction as previously described.[74]

Generation of DNA templates for *in vitro* mRNA transcription

The SDT was generated using three synthetic oligonucleotides (Ultramers; Integrated DNA Technologies [IDT]) that were designed to hybridize together during assembly PCR (KAPA HiFi HotStart ReadyMix; Roche), forming an SDT, which was further amplified by PCR. After each PCR, the formed SDT was purified (GeneJet PCR Purification Kit; Thermo Fisher Scientific). The PDT was generated using the plasmid pLMCT developed in-house. gBlocks for the different inserts were purchased from IDT and cloned into pLMCT using the Gibson assembly kit (New England Biolabs [NEB]) and XL2-Blue Ultracompetent Cells (Agilent). Cloned plasmids were sequence verified (Eurofins Genomics), and selected clones were further amplified by MIDI DNA preparation using plasmid kits from Qiagen. Each plasmid was linearized overnight by restriction enzyme digestion with BfuAI (NEB) to enable *in vitro* mRNA transcription. As quality controls, the yield (absorbance at 260/280 nm), integrity (BioAnalyzer 2100, DNA 7500 chip), and sequence (Eurofins Genomics) of both SDT and PDT were verified.

mRNA synthesis

The *i*VT reaction was performed starting from a dsDNA template using a T7 enzyme mix containing: T7 RNA polymerase (Thermo Fisher Scientific), RNase inhibitor (Promega), and inorganic pyrophosphatase (Thermo Fisher Scientific). The reaction buffer mix included 10 mM Clean CAP AG reagent (TriLink Biotech) and 10 mM of each dNTP (adenosine-, guanosine-, cytidine- and uridine-triphosphate; Promega). The reaction was incubated at 37°C

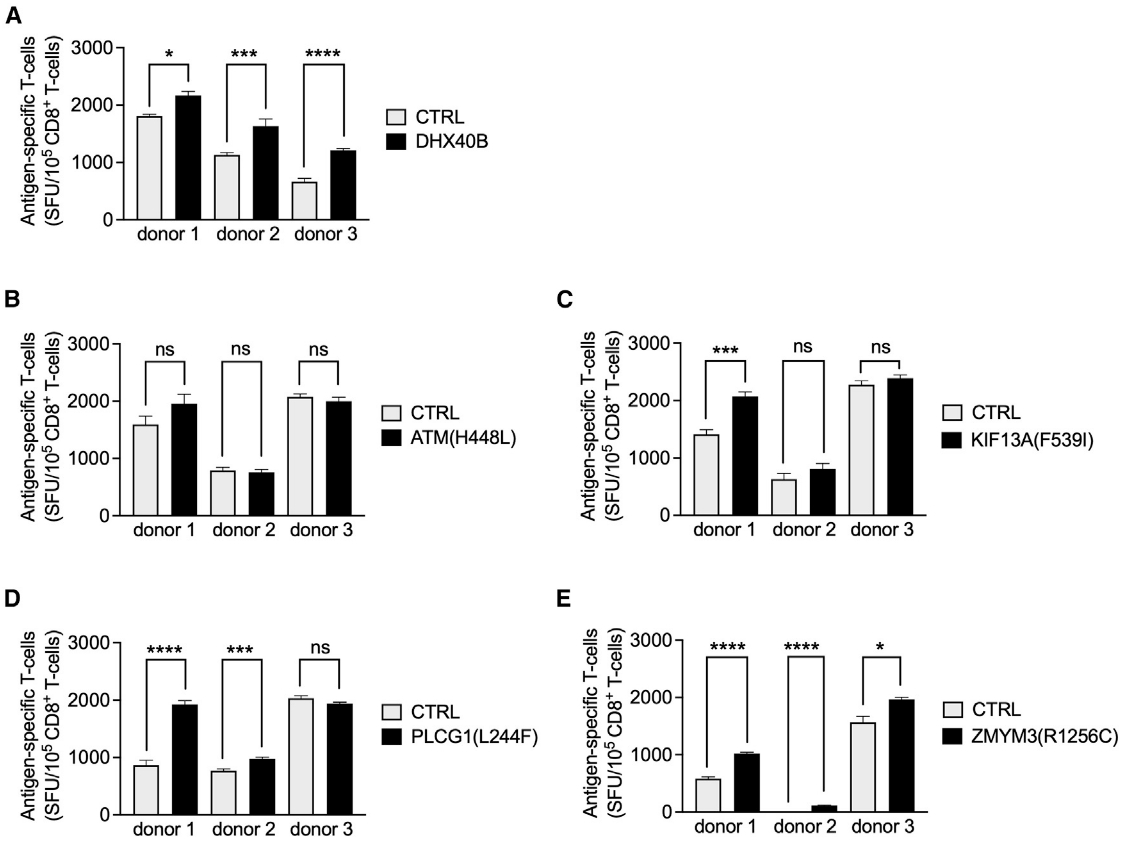

Figure 5. Screening of neoepitope immunogenicity is feasible using mRNA produced from a synthetic DNA template

(A–E) Bar graphs summarizing the number of SFU per 10^5 $CD8^+$ T cells, quantified using ELISPOT, after overnight restimulation with HLA-A2$^+$ K562 cells presenting (A) DHX40B, (B) ATM(H448L), (C) KIF13A(F539I), (D) PLCG1(L244F), or (E) ZMYM3(R1256C). Results are presented as mean ± standard error of the mean of triplicate samples for three independent experiments performed with cells of three different donors. Statistical significance was assessed using Student's t test. Statistically significant differences are shown as follows: *$p < 0.05$, ***$p < 0.001$, ****$p < 0.0001$; ns, not significant.

for 2 h. After incubation, DNaseI exonuclease (Thermo Fisher Scientific) was added to the reaction mix and incubated for 15 min at 37°C for the removal of residual dsDNA template. All enzymes added to the reaction were deactivated at 65°C for 20 min. For SDT-mRNA production, polymerase-A enzyme was added to the reaction mix for RNA polyadenylation (TebuBio poly(A) tailing kit). The reaction mix is incubated for 60 min at 37°C, after which 1.5 volumes of 40 mM EDTA solution were added to the mix to stop any further enzymatic activity. The mRNA was purified by LiCl-mediated precipitation. Half the reaction volume of 8 M LiCl (Sigma-Aldrich) was added to the mRNA solution and stored at −20°C overnight. The mRNA sample was centrifuged (15 min at 12,100 × *g*), and the obtained pellet was washed with 70% ethanol (Sigma-Aldrich) and subsequently dissolved in RNase-free water (Gibco). A second purification step was performed by NaCl/EtOH precipitation, adding 5 M NaCl (Sigma-Aldrich) and absolute ethanol (Sigma-Aldrich). The mRNA was centrifuged (15 min at 14,000 rpm), and the obtained pellet was washed with 70% ethanol and dissolved in RNase-free water (Gibco). The resulting SDT-mRNA and PDT-mRNA were subjected to quality controls, including spectrophotometric reading of optical density for the yield determination and purity (absorbance ratio at 260/280 nm), integrity (BioAnalyzer 2100, RNA 6500 chip), and cDNA sequence verification after reverse transcription (cDNA kit; NEB and Eurofins Genomics).

Transfection of mRNA to cells by electroporation

Transfection of mRNA to moDCs and T cells was performed by electroporation. Cells were extensively washed in serum-free OptiMEM (Life Technologies, Belgium). The electroporation was performed in 200 μL of OptiMEM medium in a 4-mm electroporation cuvette (Cell Projects) using the following parameters: square wave pulse, 500 V, 2 ms, 1 pulse for moDCs; and square wave pulse, 500 V, 5 ms, 1 pulse for T cells, using the Gene Pulser Xcell device (Bio-Rad, Belgium). TCRα- and TCRβ-chain mRNA (5 μg each/10^6 cells) was electroporated into $CD8^+$ T cells. Electroporation of moDCs with mRNA was performed with a total concentration of 100 μg/mL mRNA.

Antigen presentation assay

$CD8^+$ T cells were isolated from monocyte-depleted PBMCs by magnetically activated cell sorting (MACS) using positive selection

with human anti-CD8 microbeads according to the manufacturer's instructions (Miltenyi Biotec). These CD8+ T cells, electroporated with TCR mRNA, were co-cultured with moDCs electroporated with the corresponding (neo)epitope mRNA at a 1:1 ratio in the presence of IL-2 (25 IU/mL, Thermo Fisher Scientific). Cells were plated in triplicate in 96-well round-bottom plates in IMDM supplemented with 1% human AB serum (200 μL/well) for 24 h at 37°C and 5% CO_2. Supernatants from the co-cultured cells were collected to quantify IFN-γ in ELISA (Thermo Fisher Scientific) according to the manufacturer's instructions.

Stimulation of naive antigen-specific T cells

Naive CD8+ T cells were isolated from monocyte-depleted PBMCs by MACS using the CD8+ T cell isolation kit, with anti-CD45RO and anti-CD57 microbeads (Miltenyi Biotec). First the monocyte-depleted PBMCs were depleted from CD45RO- and CD57-positive cells, after which a positive selection was performed for CD8+ T cells. Cells were co-cultured with moDCs electroporated with TriMix and neoantigen mRNA at a 1:2 ratio in the presence of 30 ng/mL IL-21 (CellGenix). On days 3 and 7, IL-15 and IL-7 (Peprotech) were added at 5 ng/mL. In the case of stimulation of T cells recognizing the HLA-2-restricted, mutated Melan-A/Mart-1 (A27L) epitope, the T cells were collected and analyzed for Melan-A/Mart-1 (A27L) specificity with flow cytometry using HLA-A*02:01/ELAGIGILTV (WB2162) dextramer staining performed according to the manufacturer's instructions (Immudex, Denmark). Moreover, ELISPOT was performed to study IFN-γ production by the T cells after restimulation with HLA-A2+ and Melan-A/Mart-1 (A27L) presenting K562 cells. In the case of stimulation of T cells for candidate neoepitopes, including DHX40B, a restimulation of the CD8+ T cells was performed on day 7 using moDCs electroporated with SDT-mRNA encoding the neoepitope and PDT-mRNA encoding TriMix. On day 13, ELISPOT was performed to study IFN-γ production by the T cells after restimulation with HLA-A2+ and neoepitope presenting K562 cells.

Flow cytometry

Cells were harvested and washed twice with PBS containing 1% BSA (flow cytometry buffer). The following antibody cocktail was used to phenotype moDCs: anti-CCR7-APC (clone G043H7, BioLegend), anti-CD40-BV605 (clone 5C3, BD), anti-CD86-FITC (clone FUN-1, BD Pharmingen), anti-HLA-DR-PE-CY7 (clone G46-6, BD Pharmingen), and anti-HLA-A2-BV421 (clone BB7.2, BioLegend). Cells were also stained with 7-AAD (BioLegend) to discriminate live from dead cells. T cell phenotyping was performed using the following antibodies: anti-CD8-BV421 (clone RPA-T8, BioLegend), anti-CD45RA-PE (clone HI100, BD), anti-CD45RO-APC (clone UCHL1, BD) and MHC dextramer Mel-A-PE or -APC (HLA-A*0201/ELAGIGILTV, WB2162, Immudex) for CD8+ T cells and anti-CD4-FITC (clone OKT4, BioLegend), anti-CD69-PerCP-CY5.5 (clone FN50, BioLegend) and anti-OX40-APC (clone ACT35, BioLegend) for CD4+ T cells. T cells were also stained with 7-AAD (BioLegend) to discriminate live from dead cells. Cells were acquired on the LSR Fortessa flow cytometer and analyzed with FlowJo software, version 10.0.

Enzyme-linked immunospot assay

Production of IFN-γ by T cells was measured by ELISPOT (Diaclone). T cells previously activated against HLA-A2-restricted neoepitopes were loaded onto ELISPOT plates at 30,000 T cells per well. Restimulation was performed at a 1:1 ratio with HLA-A2+ K562 cells electroporated with mRNA encoding the corresponding neoepitope or an unrelated antigen. After 24 h of co-culture, the ELISPOT plates were developed according to the manufacturer's instructions to visualize spots, signifying IFN-γ-producing T cells. These were quantified as SFU with the aid of an ELISPOT reader (Autoimmun Diagnostika).

Bioinformatics pipeline (ImmunoEngine)

Fresh frozen tumor biopsy material and blood was obtained from an anonymized melanoma patient. Tumor tissue was isolated by cryotome slicing and microdissection to ensure maximal tumor purity. Tumor DNA material (from microdissected tumor biopsy tissue) and DNA material from blood were extracted using the Qiagen DNeasy Blood & Tissue Kit. PCR-free whole-genome sequencing libraries were constructed using the NEBNext Ultra II FS DNA module and the NEBNext Ultra II Ligation module. Tumor RNA from microdissected tumor biopsy tissue was extracted using the Qiagen RNeasy Mini Kit, and a directional RNA-sequencing library was constructed using the NEBNext Ultra II Directional RNA Library Prep Kit for Illumina. All libraries were then sequenced on a Novaseq 6000 (v1.5). Sequencing results were subsequently analyzed by myNEO's ImmunoEngine. In brief, sequencing data were aligned to the GRCh38 reference genome;[75,76] blood and tumor sequencing data were then confronted using a combination of variant callers[77,78] and custom filters to isolate a set of high-confidence, tumor-specific, DNA-borne single-nucleotide variants (SNVs) and insertion/deletions (indels) (n = 21,094 variants detected). Expression of these SNVs was further studied by assessing their presence in the tumor RNA-sequencing data[78] (n = 1,072 variants significantly expressed). In a third step, the coding potential and neoantigen load of expressed SNVs was assessed[79] (n = 71 non-synonymous expressed coding mutations), and the entire set of potential neoantigens was prioritized using a neoantigen score accounting for the following parameters: dissimilarity to self, predicted binding affinity rank as evaluated by MHCflurry,[80] and predicted likelihood to elicit a T cell response as predicted by neoIM, a proprietary algorithm. Neoantigens with the highest score were selected for validation.

Statistical analysis

All statistical analyses were performed using GraphPad Prism software, version 8.4.3. Statistical analysis on multiple datasets was performed with one-way ANOVA with Bonferroni's correction, while statistical analysis for two datasets was performed using Student's t test. Statistical analysis and significance is indicated in the figure legends.

Data availability

The authors confirm that the data supporting the findings of this study are available within the article and its supplemental information.

SUPPLEMENTAL INFORMATION

Supplemental information can be found online at https://doi.org/10.1016/j.omtn.2022.08.021.

ACKNOWLEDGMENTS

This work was performed with the financial support of the Flanders Innovation and Entrepreneurship agency (HBC.2019.2522 and HBC.2019.2564), the Belgian Foundation against Cancer (FAF-C/2018/1222 [2018-128] and FAF-F/2018/1223 [2018-089]), and the Research Council of the Vrije Universiteit Brussel under its Strategic Research Program (SRP48) and Industrial Research Fund (IOF3009). H.L. received financial support from the Flanders Innovation and Entrepreneurship agency (HBC.2017.0564). W.d.M. received financial support from the Oncology Research Center (Prijs Kankeronderzoek) and the Research Council of the Vrije Universiteit Brussel, and the Scientific Fund Willy Gepts. We thank Petra Roman, Elsy Vaeremans, and Sara Dehou (DNA template and mRNA production), Asma Lakdimi and Nada Yayhaoui (moDC generation), and Angelo Willems (flow cytometry maintenance) for the laboratory and technical work.

AUTHOR CONTRIBUTIONS

Conception and design, W.d.M., M.V., K.B., K.T., and L.F.; development of methodology, W.d.M., B.F., L.L., C.B., M.V., K.B., K.T., and L.F.; acquisition of data, W.d.M., P.D.S., D.A., L.P., B.F., H.L., A.E., M.V., and L.F.; analysis and interpretation of data, W.d.M., P.D.S., D.A., L.P., B.F., L.L., H.L., A.E., C.B., M.V., K.B., K.T., and L.F.; writing, review, and/or revision of the manuscript, W.d.M., P.D.S., D.A., L.P., B.F., H.L., A.E., L.L., C.B., M.V., K.B., K.T., and L.F.; study supervision, K.B., K.T., and L.F.

DECLARATION OF INTERESTS

The use of dendritic cells electroporated with tumor antigen and TriMix mRNA is the topic of a patent (WO2009/034172) on which K.T. is filed as an inventor. This patent is licensed to eTheRNA Immunotherapies NV. The synthetic DNA template for *in vitro* mRNA transcription is listed under patent application WO2021/185833 on which K.T., L.F., and C.B. are listed as inventors. L.P., B.F., L.L., and C.B. are employees and shareholders at myNEO NV, a company developing neoantigen immunotherapies. The prediction pipeline used for the identification of patient-specific neoantigens is the topic of two patents (NeoMS:EP20185779.4 and NeoIM:EP20202140.8) belonging to myNEO NV.

REFERENCES

1. Zhang, Y., and Zhang, Z. (2020). The history and advances in cancer immunotherapy: understanding the characteristics of tumor-infiltrating immune cells and their therapeutic implications. Cell. Mol. Immunol. *17*, 807–821. https://doi.org/10.1038/s41423-020-0488-6.
2. Waldman, A.D., Fritz, J.M., and Lenardo, M.J. (2020). A guide to cancer immunotherapy: from T cell basic science to clinical practice. Nat. Rev. Immunol. *20*, 651–668. https://doi.org/10.1038/s41577-020-0306-5.
3. Fan, L., Li, Y., Chen, J.Y., Zheng, Y.F., and Xu, X.M. (2019). Immune checkpoint modulators in cancer immunotherapy: recent advances and combination rationales. Cancer Lett. *456*, 23–28. https://doi.org/10.1016/j.canlet.2019.03.050.
4. Hegde, P.S., and Chen, D.S. (2020). Top 10 challenges in cancer immunotherapy. Immunity *52*, 17–35. https://doi.org/10.1016/j.immuni.2019.12.011.
5. Peng, M., Mo, Y., Wang, Y., Wu, P., Zhang, Y., Xiong, F., Guo, C., Wu, X., Li, Y., Li, X., et al. (2019). Neoantigen vaccine: an emerging tumor immunotherapy. Mol. Cancer *18*, 128. https://doi.org/10.1186/s12943-019-1055-6.
6. Zhang, Z., Lu, M., Qin, Y., Gao, W., Tao, L., Su, W., and Zhong, J. (2021). Neoantigen: a new breakthrough in tumor immunotherapy. Front. Immunol. *12*, 672356. https://doi.org/10.3389/fimmu.2021.672356.
7. Blass, E., and Ott, P.A. (2021). Advances in the development of personalized neoantigen-based therapeutic cancer vaccines. Nat. Rev. Clin. Oncol. *18*, 215–229. https://doi.org/10.1038/s41571-020-00460-2.
8. Curran, M.A., and Glisson, B.S. (2019). New hope for therapeutic cancer vaccines in the era of immune checkpoint modulation. Annu. Rev. Med. *70*, 409–424. https://doi.org/10.1146/annurev-med-050217-121900.
9. Lee, K.L., Schlom, J., and Hamilton, D.H. (2021). Combination therapies utilizing neoepitope-targeted vaccines. Cancer Immunol. Immunother. *70*, 875–885. https://doi.org/10.1007/s00262-020-02729-y.
10. Hu, Z., Leet, D.E., Allesøe, R.L., Oliveira, G., Li, S., Luoma, A.M., Liu, J., Forman, J., Huang, T., Iorgulescu, J.B., et al. (2021). Personal neoantigen vaccines induce persistent memory T cell responses and epitope spreading in patients with melanoma. Nat. Med. *27*, 515–525. https://doi.org/10.1038/s41591-020-01206-4.
11. Gupta, R.G., Li, F., Roszik, J., and Lizée, G. (2021). Exploiting tumor neoantigens to target cancer evolution: current challenges and promising therapeutic approaches. Cancer Discov. *11*, 1024–1039. https://doi.org/10.1158/2159-8290.
12. Richters, M.M., Xia, H., Campbell, K.M., Gillanders, W.E., Griffith, O.L., and Griffith, M. (2019). Best practices for bioinformatic characterization of neoantigens for clinical utility. Genome Med. *11*, 56. https://doi.org/10.1186/s13073-019-0666-2.
13. Roudko, V., Greenbaum, B., and Bhardwaj, N. (2020). Computational prediction and validation of tumor-associated neoantigens. Front. Immunol. *11*, 27. https://doi.org/10.3389/fimmu.2020.00027.
14. De Mattos-Arruda, L., Blanco-Heredia, J., Aguilar-Gurrieri, C., Carrillo, J., and Blanco, J. (2020). New emerging targets in cancer immunotherapy: the role of neoantigens. ESMO Open *4*, e000684. https://doi.org/10.1136/esmoopen-2020-000684.
15. Tran, E., Ahmadzadeh, M., Lu, Y.C., Gros, A., Turcotte, S., Robbins, P.F., Gartner, J.J., Zheng, Z., Li, Y.F., Ray, S., et al. (2015). Immunogenicity of somatic mutations in human gastrointestinal cancers. Science *350*, 1387–1390. https://doi.org/10.1126/science.aad1253.
16. Yossef, R., Tran, E., Deniger, D.C., Gros, A., Pasetto, A., Parkhurst, M.R., Gartner, J.J., Prickett, T.D., Cafri, G., Robbins, P.F., and Rosenberg, S.A. (2018). Enhanced detection of neoantigen-reactive T cells targeting unique and shared oncogenes for personalized cancer immunotherapy. JCI Insight *3*, 122467. https://doi.org/10.1172/jci.insight.122467.
17. Castro, A., Zanetti, M., and Carter, H. (2021). Neoantigen controversies. Annu. Rev. Biomed. Data Sci. *4*, 227–253. https://doi.org/10.1146/annurev-biodatasci-092820-112713.
18. Vitiello, A., and Zanetti, M. (2017). Neoantigen prediction and the need for validation. Nat. Biotechnol. *35*, 815–817. https://doi.org/10.1038/nbt.3932.
19. Esprit, A., de Mey, W., Bahadur Shahi, R., Thielemans, K., Franceschini, L., and Breckpot, K. (2020). Neo-Antigen mRNA vaccines. Vaccines *8*. https://doi.org/10.3390/vaccines8040776.
20. Vigneron, N., Abi Habib, J., and Van den Eynde, B.J. (2017). Learning from the proteasome how to fine-tune cancer immunotherapy. Trends Cancer *3*, 726–741. https://doi.org/10.1016/j.trecan.2017.07.007.
21. Henderson, J.M., Ujita, A., Hill, E., Yousif-Rosales, S., Smith, C., Ko, N., McReynolds, T., Cabral, C.R., Escamilla-Powers, J.R., and Houston, M.E. (2021). Cap 1 messenger RNA synthesis with Co-transcriptional CleanCap. Curr. Protoc. *1*, e39. https://doi.org/10.1002/cpz1.39.
22. Rong, M., He, B., McAllister, W.T., and Durbin, R.K. (1998). Promoter specificity determinants of T7 RNA polymerase. Proc. Natl. Acad. Sci. USA *95*, 515–519. https://doi.org/10.1073/pnas.95.2.515.

23. Kozak, M. (1986). Point mutations define a sequence flanking the AUG initiator codon that modulates translation by eukaryotic ribosomes. Cell *44*, 283–292. https://doi.org/10.1016/0092-8674(86)90762-2.

24. Kozak, M. (1987). At least six nucleotides preceding the AUG initiator codon enhance translation in mammalian cells. J. Mol. Biol. *196*, 947–950. https://doi.org/10.1016/0022-2836(87)90418-9.

25. Annweiler, A., Hipskind, R.A., and Wirth, T. (1991). A strategy for efficient in vitro translation of cDNAs using the rabbit beta-globin leader sequence. Nucleic Acids Res. *19*, 3750. https://doi.org/10.1093/nar/19.13.3750.

26. Kozak, M. (1994). Features in the 5' non-coding sequences of rabbit alpha and beta-globin mRNAs that affect translational efficiency. J. Mol. Biol. *235*, 95–110. https://doi.org/10.1016/s0022-2836(05)80019-1.

27. Conrad, N.K., and Steitz, J.A. (2005). A Kaposi's sarcoma virus RNA element that increases the nuclear abundance of intronless transcripts. EMBO J. *24*, 1831–1841. https://doi.org/10.1038/sj.emboj.7600662.

28. Jiang, Y., Xu, X.S., and Russell, J.E. (2006). A nucleolin-binding 3' untranslated region element stabilizes beta-globin mRNA in vivo. Mol. Cell Biol. *26*, 2419–2429. https://doi.org/10.1128/MCB.26.6.2419-2429.2006.

29. Pardi, N., Hogan, M.J., Porter, F.W., and Weissman, D. (2018). mRNA vaccines - a new era in vaccinology. Nat. Rev. Drug Discov. *17*, 261–279. https://doi.org/10.1038/nrd.2017.243.

30. Pardi, N., Muramatsu, H., Weissman, D., and Karikó, K. (2013). In vitro transcription of long RNA containing modified nucleosides. Methods Mol. Biol. *969*, 29–42. https://doi.org/10.1007/978-1-62703-260-5_2.

31. Ingels, J., De Cock, L., Mayer, R.L., Devreker, P., Weening, K., Heyns, K., Lootens, N., De Smet, S., Brusseel, M., De Munter, S., et al. (2022). Small-scale manufacturing of neoantigen-encoding messenger RNA for early-phase clinical trials. Cytotherapy *24*, 213–222. https://doi.org/10.1016/j.jcyt.2021.08.005.

32. Ohlson, J. (2020). Plasmid manufacture is the bottleneck of the genetic medicine revolution. Drug Discov. Today *25*, 1891–1893. https://doi.org/10.1016/j.drudis.2020.09.040.

33. Rosenberg, S.A. (2019). A Phase I/II Trial to Evaluate the Safety and Immunogenicity of a Messenger RNA (mRNA)-Based, Personalized Cancer Vaccine against Neoantigens Expressed by the Autologous Cancer (Study Protocol). https://clinicaltrials.gov/ct2/show/study/NCT03480152.

34. Bonehill, A., Heirman, C., Tuyaerts, S., Michiels, A., Breckpot, K., Brasseur, F., Zhang, Y., Van Der Bruggen, P., and Thielemans, K. (2004). Messenger RNA-electroporated dendritic cells presenting MAGE-A3 simultaneously in HLA class I and class II molecules. J. Immunol. *172*, 6649–6657. https://doi.org/10.4049/jimmunol.172.11.6649.

35. Schultz, E.S., Lethé, B., Cambiaso, C.L., Van Snick, J., Chaux, P., Corthals, J., Heirman, C., Thielemans, K., Boon, T., and van der Bruggen, P. (2000). A MAGE-A3 peptide presented by HLA-DP4 is recognized on tumor cells by CD4+ cytolytic T lymphocytes. Cancer Res. *60*, 6272–6275.

36. Cox, A.L., Skipper, J., Chen, Y., Henderson, R.A., Darrow, T.L., Shabanowitz, J., Engelhard, V.H., Hunt, D.F., and Slingluff, C.L. (1994). Identification of a peptide recognized by five melanoma-specific human cytotoxic T cell lines. Science *264*, 716–719. https://doi.org/10.1126/science.7513441.

37. Bakker, A.B., Schreurs, M.W., de Boer, A.J., Kawakami, Y., Rosenberg, S.A., Adema, G.J., and Figdor, C.G. (1994). Melanocyte lineage-specific antigen gp100 is recognized by melanoma-derived tumor-infiltrating lymphocytes. J. Exp. Med. *179*, 1005–1009. https://doi.org/10.1084/jem.179.3.1005.

38. Kawakami, Y., Eliyahu, S., Jennings, C., Sakaguchi, K., Kang, X., Southwood, S., Robbins, P.F., Sette, A., Appella, E., and Rosenberg, S.A. (1995). Recognition of multiple epitopes in the human melanoma antigen gp100 by tumor-infiltrating T lymphocytes associated with in vivo tumor regression. J. Immunol. *154*, 3961–3968.

39. Kiniwa, M., Gately, M., Gubler, U., Chizzonite, R., Fargeas, C., and Delespesse, G. (1992). Recombinant interleukin-12 suppresses the synthesis of immunoglobulin E by interleukin-4 stimulated human lymphocytes. J. Clin. Invest. *90*, 262–266. https://doi.org/10.1172/JCI115846.

40. Chen, Y.T., Scanlan, M.J., Sahin, U., Türeci, O., Gure, A.O., Tsang, S., Williamson, B., Stockert, E., Pfreundschuh, M., and Old, L.J. (1997). A testicular antigen aberrantly expressed in human cancers detected by autologous antibody screening. Proc. Natl. Acad. Sci. USA *94*, 1914–1918. https://doi.org/10.1073/pnas.94.5.1914.

41. Lethé, B., Lucas, S., Michaux, L., De Smet, C., Godelaine, D., Serrano, A., De Plaen, E., and Boon, T. (1998). LAGE-1, a new gene with tumor specificity. Int. J. Cancer *76*, 903–908. https://doi.org/10.1002/(sici)1097-0215(19980610)76:6<903::aid-ijc22>3.0.co.

42. Raza, A., Merhi, M., Inchakalody, V.P., Krishnankutty, R., Relecom, A., Uddin, S., and Dermime, S. (2020). Unleashing the immune response to NY-ESO-1 cancer testis antigen as a potential target for cancer immunotherapy. J. Transl. Med. *18*, 140. https://doi.org/10.1186/s12967-020-02306-y.

43. Zeh, H.J., Leder, G.H., Lotze, M.T., Salter, R.D., Tector, M., Stuber, G., Modrow, S., and Storkus, W.J. (1994). Flow-cytometric determination of peptide-class I complex formation. Identification of p53 peptides that bind to HLA-A2. Hum. Immunol. *39*, 79–86. https://doi.org/10.1016/0198-8859(94)90105-8.

44. Houbiers, J.G., Nijman, H.W., van der Burg, S.H., Drijfhout, J.W., Kenemans, P., van de Velde, C.J., Brand, A., Momburg, F., Kast, W.M., and Melief, C.J. (1993). In vitro induction of human cytotoxic T lymphocyte responses against peptides of mutant and wild-type p53. Eur. J. Immunol. *23*, 2072–2077. https://doi.org/10.1002/eji.1830230905.

45. Pittet, M.J., Valmori, D., Dunbar, P.R., Speiser, D.E., Liénard, D., Lejeune, F., Fleischhauer, K., Cerundolo, V., Cerottini, J.C., and Romero, P. (1999). High frequencies of naive Melan-A/MART-1-specific CD8(+) T cells in a large proportion of human histocompatibility leukocyte antigen (HLA)-A2 individuals. J. Exp. Med. *190*, 705–715. https://doi.org/10.1084/jem.190.5.705.

46. Dörrie, J., Schaft, N., Schuler, G., and Schuler-Thurner, B. (2020). Therapeutic cancer vaccination with ex vivo RNA-transfected dendritic cells-an update. Pharmaceutics *12*, E92.

47. De Keersmaecker, B., Claerhout, S., Carrasco, J., Bar, I., Corthals, J., Wilgenhof, S., Neyns, B., and Thielemans, K. (2020). TriMix and tumor antigen mRNA electroporated dendritic cell vaccination plus ipilimumab: link between T-cell activation and clinical responses in advanced melanoma. J. Immunother. Cancer *8*, e000329. https://doi.org/10.1136/jitc-2019-000329.

48. Wilgenhof, S., Corthals, J., Heirman, C., van Baren, N., Lucas, S., Kvistborg, P., Thielemans, K., and Neyns, B. (2016). Phase II study of autologous monocyte-derived mRNA electroporated dendritic cells (TriMixDC-MEL) plus ipilimumab in patients with pretreated advanced melanoma. J. Clin. Oncol. *34*, 1330–1338. https://doi.org/10.1200/JCO.2015.63.4121.

49. Heil, F., Hemmi, H., Hochrein, H., Ampenberger, F., Kirschning, C., Akira, S., Lipford, G., Wagner, H., and Bauer, S. (2004). Species-specific recognition of single-stranded RNA via toll-like receptor 7 and 8. Science *303*, 1526–1529. https://doi.org/10.1126/science.1093620.

50. Leonard, J.N., Ghirlando, R., Askins, J., Bell, J.K., Margulies, D.H., Davies, D.R., and Segal, D.M. (2008). The TLR3 signaling complex forms by cooperative receptor dimerization. Proc. Natl. Acad. Sci. USA *105*, 258–263. https://doi.org/10.1073/pnas.0710779105.

51. Rehwinkel, J., and Gack, M.U. (2020). RIG-I-like receptors: their regulation and roles in RNA sensing. Nat. Rev. Immunol. *20*, 537–551. https://doi.org/10.1038/s41577-020-0288-3.

52. DeNardo, D.G., Barreto, J.B., Andreu, P., Vasquez, L., Tawfik, D., Kolhatkar, N., and Coussens, L.M. (2009). CD4(+) T cells regulate pulmonary metastasis of mammary carcinomas by enhancing protumor properties of macrophages. Cancer Cell *16*, 91–102. https://doi.org/10.1016/j.ccr.2009.06.018.

53. Perez-Diez, A., Joncker, N.T., Choi, K., Chan, W.F.N., Anderson, C.C., Lantz, O., and Matzinger, P. (2007). CD4 cells can be more efficient at tumor rejection than CD8 cells. Blood *109*, 5346–5354. https://doi.org/10.1182/blood-2006-10-051318.

54. Boissonnas, A., Fetler, L., Zeelenberg, I.S., Hugues, S., and Amigorena, S. (2007). In vivo imaging of cytotoxic T cell infiltration and elimination of a solid tumor. J. Exp. Med. *204*, 345–356. https://doi.org/10.1084/jem.20061890.

55. Lee, M.Y., Jeon, J.W., Sievers, C., and Allen, C.T. (2020). Antigen processing and presentation in cancer immunotherapy. J. Immunother. Cancer *8*, e001111. https://doi.org/10.1136/jitc-2020-001111.

56. Bonehill, A., Tuyaerts, S., Van Nuffel, A.M.T., Heirman, C., Bos, T.J., Fostier, K., Neyns, B., and Thielemans, K. (2008). Enhancing the T-cell stimulatory capacity of human dendritic cells by co-electroporation with CD40L, CD70 and constitutively

active TLR4 encoding mRNA. Mol. Ther. *16*, 1170–1180. https://doi.org/10.1038/mt.2008.77.

57. Pittet, M.J., Gati, A., Le Gal, F.A., Bioley, G., Guillaume, P., de Smedt, M., Plum, J., Speiser, D.E., Cerottini, J.C., Dietrich, P.Y., et al. (2006). Ex vivo characterization of allo-MHC-restricted T cells specific for a single MHC-peptide complex. J. Immunol. *176*, 2330–2336. https://doi.org/10.4049/jimmunol.176.4.2330.

58. Ali, M., Foldvari, Z., Giannakopoulou, E., Böschen, M.L., Strønen, E., Yang, W., Toebes, M., Schubert, B., Kohlbacher, O., Schumacher, T.N., and Olweus, J. (2019). Induction of neoantigen-reactive T cells from healthy donors. Nat. Protoc. *14*, 1926–1943. https://doi.org/10.1038/s41596-019-0170-6.

59. De Mattos-Arruda, L., Blanco-Heredia, J., Aguilar-Gurrieri, C., Carrillo, J., and Blanco, J. (2020). New emerging targets in cancer immunotherapy: the role of neoantigens. ESMO Open *4*, e000684. https://doi.org/10.1136/esmoopen-2020-000684.

60. Ott, P.A., Hu, Z., Keskin, D.B., Shukla, S.A., Sun, J., Bozym, D.J., Zhang, W., Luoma, A., Giobbie-Hurder, A., Peter, L., et al. (2017). An immunogenic personal neoantigen vaccine for patients with melanoma. Nature *547*, 217–221. https://doi.org/10.1038/nature22991.

61. Shemesh, C.S., Hsu, J.C., Hosseini, I., Shen, B.Q., Rotte, A., Twomey, P., Girish, S., and Wu, B. (2021). Personalized cancer vaccines: clinical landscape, challenges, and opportunities. Mol. Ther. *29*, 555–570. https://doi.org/10.1016/j.ymthe.2020.09.038.

62. Shetty, K., and Ott, P.A. (2021). Personal neoantigen vaccines for the treatment of cancer. Annu. Rev. Cancer Biol. *5*, 259–276. https://doi.org/10.1146/annurev-cancer-bio-060820-111701.

63. (2017). The problem with neoantigen prediction. Nat. Biotechnol. *35*, 97. https://doi.org/10.1038/nbt.3800.

64. Cafri, G., Gartner, J.J., Zaks, T., Hopson, K., Levin, N., Paria, B.C., Parkhurst, M.R., Yossef, R., Lowery, F.J., Jafferji, M.S., et al. (2020). mRNA vaccine-induced neoantigen-specific T cell immunity in patients with gastrointestinal cancer. J. Clin. Invest. *130*, 5976–5988. https://doi.org/10.1172/JCI134915.

65. Sahin, U., Derhovanessian, E., Miller, M., Kloke, B.P., Simon, P., Löwer, M., Bukur, V., Tadmor, A.D., Luxemburger, U., Schrörs, B., et al. (2017). Personalized RNA mutanome vaccines mobilize poly-specific therapeutic immunity against cancer. Nature *547*, 222–226. https://doi.org/10.1038/nature23003.

66. Sahin, U., and Türeci, Ö. (2018). Personalized vaccines for cancer immunotherapy. Science *359*, 1355–1360. https://doi.org/10.1126/science.aar7112.

67. Strønen, E., Toebes, M., Kelderman, S., van Buuren, M.M., Yang, W., van Rooij, N., Donia, M., Böschen, M.L., Lund-Johansen, F., Olweus, J., and Schumacher, T.N. (2016). Targeting of cancer neoantigens with donor-derived T cell receptor repertoires. Science *352*, 1337–1341. https://doi.org/10.1126/science.aaf2288.

68. Lazdun, Y., Si, H., Creasy, T., Ranade, K., Higgs, B.W., Streicher, K., and Durham, N.M. (2021). A new pipeline to predict and confirm tumor neoantigens predict better response to immune checkpoint blockade. Mol. Cancer Res. *19*, 498–506. https://doi.org/10.1158/1541-7786.

69. Schubert, B., and Kohlbacher, O. (2016). Designing string-of-beads vaccines with optimal spacers. Genome Med. *8*, 9. https://doi.org/10.1186/s13073-016-0263-6.

70. Dorigatti, E., and Schubert, B. (2020). Joint epitope selection and spacer design for string-of-beads vaccines. Bioinformatics *36*, i643–i650. https://doi.org/10.1093/bioinformatics/btaa790.

71. De Keersmaecker, B., Heirman, C., Allard, S., Bonehill, A., Corthals, J., Thielemans, K., and Aerts, J.L. (2010). Lumenal part of the DC-LAMP protein is not required for induction of antigen-specific T cell responses by means of antigen-DC-LAMP messenger RNA-electroporated dendritic cells. Hum. Gene Ther. *21*, 479–485. https://doi.org/10.1089/hum.2009.080.

72. Hu, Z., Wang, L., Shi, Z., Jiang, J., Li, X., Chen, Y., Li, K., and Luo, D. (2019). Customized one-step preparation of sgRNA transcription templates via overlapping PCR Using short primers and its application in vitro and in vivo gene editing. Cell Biosci. *9*, 87. https://doi.org/10.1186/s13578-019-0350-7.

73. Paria, B.C., Levin, N., Lowery, F.J., Pasetto, A., Deniger, D.C., Parkhurst, M.R., Yossef, R., Kim, S.P., Florentin, M., Ngo, L.T., et al. (2021). Rapid identification and evaluation of neoantigen-reactive T-cell receptors from single cells. J. Immunother. *44*, 1–8. https://doi.org/10.1097/CJI.0000000000000342.

74. Awad, R.M., Lecocq, Q., Zeven, K., Ertveldt, T., De Beck, L., Ceuppens, H., Broos, K., De Vlaeminck, Y., Goyvaerts, C., Verdonck, M., et al. (2021). Formatting and gene-based delivery of a human PD-L1 single domain antibody for immune checkpoint blockade. Mol. Ther. Methods Clin. Dev. *22*, 172–182. https://doi.org/10.1016/j.omtm.2021.05.017.

75. Li, H. (2013). Aligning Sequence Reads, Clone Sequences and Assembly Contigs with BWA-MEM (arxiv:Genomics). http://arxiv.org/abs/1303.3997.

76. Dobin, A., Davis, C.A., Schlesinger, F., Drenkow, J., Zaleski, C., Jha, S., Batut, P., Chaisson, M., and Gingeras, T.R. (2013). STAR: ultrafast universal RNA-seq aligner. Bioinformatics *29*, 15–21. https://doi.org/10.1093/bioinformatics/bts635.

77. Benjamin, D., Sato, T., Cibulskis, K., Getz, G., Stewart, C., and Lichtenstein, L. (2019). Calling somatic SNVs and indels with Mutect2. Preprint at bioRxiv. https://doi.org/10.1101/861054.

78. Kim, S., Scheffler, K., Halpern, A.L., Bekritsky, M.A., Noh, E., Källberg, M., Chen, X., Kim, Y., Beyter, D., Krusche, P., and Saunders, C.T. (2018). Strelka2: fast and accurate calling of germline and somatic variants. Nat. Methods *15*, 591–594. https://doi.org/10.1038/s41592-018-0051-x.

79. Bjerregaard, A.M., Nielsen, M., Hadrup, S.R., Szallasi, Z., and Eklund, A.C. (2017). MuPeXI: prediction of neo-epitopes from tumor sequencing data. Cancer Immunol. Immunother. *66*, 1123–1130. https://doi.org/10.1007/s00262-017-2001-3.

80. Shao, X.M., Bhattacharya, R., Huang, J., Sivakumar, I.K.A., Tokheim, C., Zheng, L., Hirsch, D., Kaminow, B., Omdahl, A., Bonsack, M., et al. (2020). High-throughput prediction of MHC class I and II neoantigens with MHCnuggets. Cancer Immunol. Res. *8*, 396–408. https://doi.org/10.1158/2326-6066.cir-19-0464.

OMTN, Volume *29*

Supplemental information

A synthetic DNA template for fast manufacturing of versatile single epitope mRNA

Wout de Mey, Phaedra De Schrijver, Dorien Autaers, Lena Pfitzer, Bruno Fant, Hanne Locy, Arthur Esprit, Lien Lybaert, Cedric Bogaert, Magali Verdonck, Kris Thielemans, Karine Breckpot, and Lorenzo Franceschini

Supplementary material

Table S1: Oligonucleotides used for synthetic DNA template production. Abbreviations: A2, restricted to HLA-A2; DP4, restricted to HLA-DP4; FW, forward; RV reverse; Oligo, oligonucleotide synthetic polymer.

Oligo 1 (FW 5′-3′)	TAATACGACTCACTATAAGGAATACAAGCTACTTGTTCTTTTTGCATCACAACCAGGCCTCCACAACCATGGCGGCCCCTGGTAGTGCAAGGAGGCCCCTCCTGCTGCTTCTGTTGCTTTTGCTGTTAGGCTTGATGCAC
Oligo 3 (RV 5′-3′)	TTTTTTTTTGTCGACAATATACACTGGGATAAAAACCTTGCCGTCTGGTCACTGGAGGTGTCCGGTGCGAACAAGGAAAAACCCAGCCAAAACACTCGAGTCAAATCCTCTGATAGCCTGAACTTTGACAGCGAAGGCGAATTTTATACACGCCCATGCCCATCAAGCAGAGGCCGACAACTATCGCTCC
Oligo 2 (gp100-A2 RV 5′-3′)	AGGCCGACAACTATCGCTCCTATAACTGGCAAAACAATAGTGTAGTCAGAAGATGGCGGGATGGCTGCTTGCAAAACGACCTGAGCAGTGACTGGGCCGGGTTCAAGGTACGTATGTGTGACAACCAGCGCTCTGCTAGCCGCAGAGGCACAGTGCATCAAGCCTAACAGCA
Oligo 2 (NY-ESO-1-A2 RV 5′-3′)	AGGCCGACAACTATCGCTCCTATAACTGGCAAAACAATAGTGTAGTCAGAAGATGGCGGTTGCGCCAGAAAGACCGGAAGAAAAACTTGTGTTATCCACATAAGCAGTGACAATTGCTGCAAGCAAGAAGATATGCTAGCCGCAGAGGCACAGTGCATCAAGCCTAACAGCA
Oligo 2 (p53-A2 RV 5′-3′)	AGGCCGACAACTATCGCTCCTATAACTGGCAAAACAATAGTGTAGTCAGAAGATGGATCTCGGCCAGGACAGGCACAGACCCTAACTTCAAAACTGTTGCGTCCGAGCAAGTTACCGCTACTATCCTCCAGAGTAATAGCCGCAGAGGCACAGTGCATCAAGCCTAACAGCA
Oligo 2 (MAGE-A3-DP4 RV 5′-3′)	AGGCCGACAACTATCGCTCCTATAACTGGCAAAACAATAGTGTAGTCAGAAGATGGGCCAGGTACTTGTCGATATTCCAAATAGTTTTCTTGTACGAAGTGTTGAGTGAGAAGTTTCTTTGGGTCTCCAAGGATGCTAGCCGCAGAGGCACAGTGCATCAAGCCTAACAGCA
Oligo 2 (Mel-A MUT-A2 RV 5′-3′)	AGGCCGACAACTATCGCTCCTATAACTGGCAAAACAATAGTGTAGTCAGAAGATGGGCCAATGAGCAACAGGACCCCAAGAATTACTGTCAGAATACCTATGCCAGCGAGTTCCTCGGCAGTCGTGTAACTATGGCCAGCCGCAGAGGCACAGTGCATCAAGCCTAACAGCA
Oligo 2 (DHX40b+C-A2 RV 5′-3′)	AGGCCGACAACTATCGCTCCTATAACTGGCAAAACAATAGTGTAGTCAGAAGATGGTCCTCTGCTAGACTCTCTCAGGGACACCAGGCCGTGCATCAGGATGCTCTTGTCGTCGTTCCGCCGCTGCATCTTAGCCGCAGAGGCACAGTGCATCAAGCCTAACAGCA
Oligo 2 (ATM(H448L)-A2 RV 5‘-3’)	AGGCCGACAACTATCGCTCCTATAACTGGCAAAACAATAGTGTAGTCAGAAGATGGCTCGGTCAGACATCTCAGCACGTAGGGTGTTCTCTCGCCCAGTCTCTGCTGAGGCAGCAGCTGGCTCAGGATCATCAGCAGAGCCGCAGAGGCACAGTGCATCAAGCCTAACAGCA
Oligo 2 (KIF13A(F539I)-A2 RV 5‘-3’)	AGGCCGACAACTATCGCTCCTATAACTGGCAAAACAATAGTGTAGTCAGAAGATGGCAGCCAATCTCTCCGCTTCCGCTTAGGCAGGTTGATCCGGATGAAGTGGTTGTTGCCCCACAGGATTCTATCGCCGTGCCAAGCCGCAGAGGCACAGTGCATCAAGCCTAACAGCA
Oligo 2 (PLCG1(L244F)-A2 RV 5‘-3’)	AGGCCGACAACTATCGCTCCTATAACTGGCAAAACAATAGTGTAGTCAGAAGATGGAAGTCTATCAACGGCCCACAGCTCGCCCTGGTAGTCCAGGAAGAACTGCTGGAACTCAGGCAGGGACACTCTGCACAGCTCAGCCGCAGAGGCACAGTGCATCAAGCCTAACAGCA
Oligo 2 (ZMYM3(R1256C)-A2 RV 5‘-3’)	AGGCCGACAACTATCGCTCCTATAACTGGCAAAACAATAGTGTAGTCAGAAGATGGTGTATCTCTGCCCTTTCTCTGCCGCACGGGAGCGTAGTAGCAGATGGACACCACCTTGGTGGTGCCTCTAGGTGTTGTGCAAGCCGCAGAGGCACAGTGCATCAAGCCTAACAGCA

Table S2: Sequence of the pLMCT (plasmid) and cDNA encoding the gene of interest (gBlock) used for plasmid DNA template production.

Abbreviations: A2, restricted to HLA-A2; DP4, restricted to HLA-DP4; FW, forward; RV reverse; SigDCL, HLA-II sorting signal of DC-LAMP.

pLMCT<br>Empty vector	TGACTCGAGTGTTTAAACCAGCCTCAAGAACACCCGAATGGAGTCTCTAAGCTACATAATACCAACTTACACTTTACAAAATGTTGTCCCCCAAAATGTAGCCATTCGTATCTGCTCCTAATAAAAAGAAA<br>GTTTCTTCACTATTGTCGACAAAAAAAAAAAAAAAAAAAAAAAAAAAAAAAAAAAAAAAAAAAAAAAAAAAAAAAAAAAAAAAAAAAAAAAAAAAAAAAAAAAAAAAAAAAAAAAAAAAAAAAAAAAAAAAAAAAAAAAAAAAAA AAAAAA<br>GCAGGTGTGTCTCTCTCCACCTGCGAATTCACTGGCCGTCGTTTTACAACGTCGTGACTGGGAAAACCCTGGCGTTACCCAACTTAATCGCCTTGCAGCACATCCCCCTTTCGCCAGCTGGCGTAATAG<br>CGAAGAGGCCCGCACCGATCGCCCTTCCCAACAGTTGCGCAGCCTGAATGGCGAATGGCGCCTGATGCGGTATTTTCTCCTTACGCATCTGTGCGGTATTTCACACCGCATATGGTGCACTCTCAGTAC<br>AATCTGCTCTGATGCCGCATAGTTAAGCCAGCCCCGACACCCGCCAACACCCGCTGACGCGCCCTGACGGGCTTGTCTGCTCCCGGCATCCGCTTACAGACAAGCTGTGACCGTCTCCGGGAGCTGCA<br>TGTGTCAGAGGTTTTCACCGTCATCACCGAAACGCGCGAGACGAAAGGGCCTCGTGATACGCCTATTTTTATAGGTTAATGTCATGATAATAATGGTTTCTTAGACGTCAGGTGGCACTTTTCGGGGAAA<br>TGTGCGCGGAACCCCTATTTGTTTATTTTTCTAAATACATTCAAATATGTATCCGCTCATGAGACAATAACCCTGATAAATGCTTCAATAATATTGAAAAAGGAAGAGTATGAGTATTCAACATTTCCGTGT<br>CGCCCTTATTCCCTTTTTTGCGGCATTTTGCCTTCCTGTTTTTGCTCACCCAGAAACGCTGGTGAAAGTAAAAGATGCTGAAGATCAGTTGGGTGCACGAGTGGGTTACATCGAACTGGATCTCAACAGC<br>GGTAAGATCCTTGAGAGTTTTCGCCCCGAAGAACGTTTTCCAATGATGAGCACTTTTAAAGTTCTGCTATGTGGCGCGGTATTATCCCGTATTGACGCCGGGCAAGAGCAACTCGGTCGCCGCATACAC<br>TATTCTCAGAATGACTTGGTTGAGTACTCACCAGTCACAGAAAAGCATCTTACGGATGGCATGACAGTAAGAGAATTATGCAGTGCTGCCATAACCATGAGTGATAACACTGCGGCCAACTTACTTCTGA<br>CAACGATCGGAGGACCGAAGGAGCTAACCGCTTTTTTGCACAACATGGGGGATCATGTAACTCGCCTTGATCGTTGGGAACCGGAGCTGAATGAAGCCATACCAAACGACGAGCGTGACACCACGATG<br>CCTGTAGCAATGGCAACAACGTTGCGCAAACTATTAACTGGCGAACTACTTACTCTAGCTTCCCGGCAACAATTAATAGACTGGATGGAGGCGGATAAAGTTGCAGGACCACTTCTGCGCTCGGCCCTT<br>CCGGCTGGCTGGTTTATTGCTGATAAATCTGGAGCCGGTGAGCGTGGGTCTCGCGGTATCATTGCAGCACTGGGGCCAGATGGTAAGCCCTCCCGTATCGTAGTTATCTACACGACGGGGAGTCAGGC<br>AACTATGGATGAACGAAATAGACAGATCGCTGAGATAGGTGCCTCACTGATTAAGCATTGGTAACTGTCAGACCAAGTTTACTCATATATACTTTAGATTGATTTAAAACTTCATTTTTAATTTAAAAGGAT<br>CTAGGTGAAGATCCTTTTTGATAATCTCATGACCAAAATCCCTTAACGTGAGTTTTCGTTCCACTGAGCGTCAGACCCCGTAGAAAAGATCAAAGGATCTTCTTGAGATCCTTTTTTTCTGCGCGTAATCT<br>GCTGCTTGCAAACAAAAAAACCACCGCTACCAGCGGTGGTTTGTTTGCCGGATCAAGAGCTACCAACTCTTTTTCCGAAGGTAACTGGCTTCAGCAGAGCGCAGATACCAAATACTGTTCTTCTAGTGTA<br>GCCGTAGTTAGGCCACCACTTCAAGAACTCTGTAGCACCGCCTACATACCTCGCTCTGCTAATCCTGTTACCAGTGGCTGCTGCCAGTGGCGATAAGTCGTGTCTTACCGGGTTGGACTCAAGACGATA<br>GTTACCGGATAAGGCGCAGCGGTCGGGCTGAACGGGGGGTTCGTGCACACAGCCCAGCTTGGAGCGAACGACCTACACCGAACTGAGATACCTACAGCGTGAGCTATGAGAAAGCGCCACGCTTCCC<br>GAAGGGAGAAAGGCGGACAGGTATCCGGTAAGCGGCAGGGTCGGAACAGGAGAGCGCACGAGGGAGCTTCCAGGGGGAAACGCCTGGTATCTTTATAGTCCTGTCGGGTTTCGCCACCTCTGACTT<br>GAGCGTCGATTTTTGTGATGCTCGTCAGGGGGGCGGAGCCTATGGAAAAACGCCAGCAACGCGGCCTTTTTACGGTTCCTGGCCTTTTGCTGGCCTTTTGCTCACATGTTCTTTCCTGCGTTATCCCCTG<br>ATTCTGTGGATAACCGTATTACCGCCTTTGAGTGAGCTGATACCGCTCGCCGCAGCCGAACGACCGAGCGCAGCGAGTCAGTGAGCGAGGAAGCGGAAGAGCGCCCAATACGCAAACCGCCTCTCCC<br>CGCGCGTTGGCCGATTCATTAATGCAGCTGGCACGACAGGTTTCCCGACTGGAAAGCGGGCAGTGAGCGCAACGCAATTAATGTGAGTTAGCTCACTCATTAGGCACCCCAGGCTTTACACTTTATGCT<br>TCCGGCTCGTATGTTGTGTGGAATTGTGAGCGGATAACAATTTCACACAGGAAACAGCTATGACCATGATTACGCCAAGCTTTAATACGACTCACTATAAGGAATACAAGCTACTTGTTCTTTTTGCATCA<br>CAACCAGGCCTCCACAACCATG
p53-A2 SigDCL	CTTGTTCTTTTTGCATCACAACCAGGCCTCCACAACCATGGCGGCCCCTGGTAGTGCAAGGAGGCCCCTCCTGCTGCTTCTGTTGCTTTTGCTGTTAGGCTTGATGCACTGTGCCTCTGCGGCTATTACT<br>CTGGAGGATAGTAGCGGTAACTTGCTCGGACGCAACAGTTTTGAAGTTAGGGTCTGTGCCTGTCCTGGCCGAGATCCATCTTCTGACTACACTATTGTTTTGCCAGTTATAGGAGCGATAGTTGTCGGC<br>CTCTGCTTGATGGGCATGGGCGTGTATAAAATTCGCCTTCGCTGTCAAAGTTCAGGCTATCAGAGGATTTGATAACTCGAGTGTTTAAACCAGCCTCAAGAACACCCGAATGGAG
gp100-A2 SigDCL	CTTGTTCTTTTTGCATCACAACCAGGCCTCCACAACCATGGCGGCCCCTGGTAGTGCAAGGAGGCCCCTCCTGCTGCTTCTGTTGCTTTTGCTGTTAGGCTTGATGCACTGTGCCTCTGCGGCTAGCAGA<br>GCGCTGGTTGTCACACATACGTACCTTGAACCCGGCCCAGTCACTGCTCAGGTCGTTTTGCAAGCAGCCATCCCGCCATCTTCTGACTACACTATTGTTTTGCCAGTTATAGGAGCGATAGTTGTCGGCC<br>TCTGCTTGATGGGCATGGGCGTGTATAAAATTCGCCTTCGCTGTCAAAGTTCAGGCTATCAGAGGATTTGATAACTCGAGTGTTTAAACCAGCCTCAAGAACACCCGAATGGAG
NY-ESO-1-A2 SigDCL	CTTGTTCTTTTTGCATCACAACCAGGCCTCCACAACCATGGCGGCCCCTGGTAGTGCAAGGAGGCCCCTCCTGCTGCTTCTGTTGCTTTTGCTGTTAGGCTTGATGCACTGTGCCTCTGCGGCTAGCATA<br>TCTTCTTGCTTGCAGCAATTGTCACTGCTTATGTGGATAACACAAGTTTTTCTTCCGGTCTTTCTGGCGCAACCGCCATCTTCTGACTACACTATTGTTTTGCCAGTTATAGGAGCGATAGTTGTCGGCCT<br>CTGCTTGATGGGCATGGGCGTGTATAAAATTCGCCTTCGCTGTCAAAGTTCAGGCTATCAGAGGATTTGATAACTCGAGTGTTTAAACCAGCCTCAAGAACACCCGAATGGAG

Melan-A MUT-A2 SigDCL	CTTGTTCTTTTTGCATCACAACCAGGCCTCCACAACCATGGCGGCCCCTGGTAGTGCAAGGAGGCCCCTCCTGCTGCTTCTGTTGCTTTTGCTGTTAGGCTTGATGCACTGTGCCTCTGCGGCTGGCCAT<br>AGTTACACGACTGCCGAGGAACTCGCTGGCATAGGTATTCTGACAGTAATTCTTGGGGTCCTGTTGCTCATTGGCCCATCTTCTGACTACACTATTGTTTTGCCAGTTATAGGAGCGATAGTTGTCGGCC<br>TCTGCTTGATGGGCATGGGCGTGTATAAAATTCGCCTTCGCTGTCAAAGTTCAGGCTATCAGAGGATTTGATAACTCGAGTGTTTAAACCAGCCTCAAGAACACCCGAATGGAG
MAGE-A3-DP4 SigDCL	CTTGTTCTTTTTGCATCACAACCAGGCCTCCACAACCATGAGCATCCTTGGAGACCCAAAGAAACTTCTCACTCAACACTTCGTACAAGAAAACTATTTGGAATATCGACAAGTACCTGGCTGATAACTCG<br>AGTGTTTAAACCAGCCTCAAGAACACCCGAATGGAG

Table S3: Sequence of the gBlocks used to clone pLMCT plasmids encoding T-cell receptor alpha or beta chains. Abbreviations: A2, restricted to HLA-A2; DP4, restricted to HLA-DP4; TCRα, T-cell receptor alpha chain; TCRβ, T-cell receptor beta chain.

p53-A2, TCRα	ACTTGTTCTTTTTGCATCACAACCAGGCCTCCACAACCATGTTACTGGCGCTCCTCCCAGTGCTGGGGATACACTTTGTCCTGAGAGATGCCCAAGCTCAGTCAGTGACGCAGCCCGATGCTCGCGTCACTGTC<br>TCTGAAGGAGCCTCTCTGCAGCTGAGATGCAAGTATTCCTACTCTGGGACACCTTATCTGTTCTGGTATGTCCAGTACCCGCGGCAGGGGCTGCAGCTGCTCCTCAAGTACTATTCAGGAGACCCAGTGGTTCA<br>AGGAGTGAATGGCTTCGAGGCTGAGTTCAGCAAGAGTAACTCTTCCTTTCATCTGCGGAAAGCCTCTGTGCACTGGAGCGACTCTGCTGTGTACTTCTGTGTTTTGAGCGAGGATAGCAACTATCAGTTGATCT<br>GGGGCTCTGGGACCAAGCTAATTATAAAGCCAGACATCCAGAACCCAGAGCCTGCTGTGTACCAGTTAAAAGATCCTCGGTCTCAGGACAGCACCCTCTGCCTGTTCACCGACTTTGACTCCCAAATCAATGTG<br>CCGAAAACAATGGAATCTGGAACGTTCATCACTGACAAAACTGTGCTGGACATGAAAGCTATGGATTCCAAGAGCAATGGGGCCATTGCCTGGAGCAACCAGACAAGCTTCACATGCCAAGATATCTTCAAAGA<br>GACCAACGCCACCTACCCCAGTTCAGACGTTCCCTGTGATGCCACGTTGACTGAGAAAAGCTTTGAAACAGATATGAACCTAAACTTTCAAAACCTGTCAGTTATGGGACTCCGAATCCTCCTGCTGAAAGTAGC<br>CGGATTTAATCTGCTCATGACGCTGAGGCTGTGGTCCAGTTAACTCGAGTGTTTAAACCAGCCTCAAGAACACCCGAATG
p53-A2, TCRβ	ACTTGTTCTTTTTGCATCACAACCAGGCCTCCACAACCATGGCTACAAGGCTCCTCTGTTACACAGTACTTTGTCTCCTGGGTGCAAGAATTTTGAATTCAAAAGTCATTCAGACTCCAAGATATCTGGTGAAAGG<br>GCAAGGACAAAAAGCAAAGATGAGGTGTATCCCTGAAAAGGGACATCCGGTGGTCTTCTGGTACCAGCAGAACAAGAACAACGAGTTCAAGTTCTTGATTAACTTCCAGAATCAAGAAGTTCTTCAGCAAATAG<br>ACATGACTGAAAAACGATTCTCTGCTGAGTGTCCTTCAAACTCACCTTGCAGCCTAGAAATTCAGTCCTCTGAGGCAGGAGACTCAGCACTGTACCTCTGTGCCAGCAGTCTGTCAGGGGGCGGCACAGAAGTC<br>TTCTTTGGTAAAGGAACCAGACTCACAGTTGTAGAGGATCTGAGAAATGTGACTCCACCCAAGGTCTCCTTGTTTGAGCCATCAAAAGCAGAGATTGCAAACAAACAAAAGGCTACCCTCGTGTGCTTGGCCAG<br>GGGCTTCTTCCCTGACCACGTGGAGCTGAGCTGGTGGGTGAATGGCAAGGAGGTCCACAGTGGGGTCAGCACGGACCCTCAGGCCTACAAGGAGAGCAATTATAGCTACTGCCTGAGCAGCCGCCTGAGGG<br>TCTCTGCTACCTTCTGGCACAATCCTCGCAACCACTTCCGCTGCCAAGTGCAGTTCCATGGGCTTTCAGAGGAGGACAAGTGGCCAGAGGGCTCACCCAAACCTGTCACACAGAACATCAGTGCAGAGGCCTG<br>GGGCCGAGCAGACTGTGGGATTACCTCAGCATCCTATCAACAAGGGGTCTTGTCTGCCACCATCCTCTATGAGATCCTGCTAGGGAAAGCCACCCTGTATGCTGTGCTTGTCAGTACACTGGTGGTGATGGCT<br>ATGGTCAAAAGAAAGAATTCATAACTCGAGTGTTTAAACCAGCCTCAAGAACACCCGAATG
gp100-A2, TCRα	ACTTGTTCTTTTTGCATCACAACCAGGCCTCCACAACCATGGCATCCATTCGAGCTGTATTTATATTCCTGTGGCTGCAGCTGGACTTGGTGAATGGAGAGAATGTGGAGCAGCATCCTTCAACCCTGAGTGTCC<br>AGGAGGGAGACAGCGCTGTTATCAAGTGTACTTATTCAGACAGTGCCTCAAACTACTTCCCTTGGTATAAGCAAGAACTTGGAAAAGGACCTCAGCTTATTATAGACATTCGTTCAAATGTGGGCGAAAAGAAA<br>GACCAACGAATTGCTGTTACATTGAACAAGACAGCCAAACATTTCTCCCTGCACATCACAGAGACCCAACCTGAAGACTCGGCTGTCTACTTCTGTGCAGCAAGTACTTCGGGTGGTACTAGCTATGGAAAGCT<br>GACATTTGGACAAGGGACCATCTTGACTGTCCATCCAAATATCCAGAACCCTGACCCTGCCGTGTACCAGCTGAGAGACTCTAAATCCAGTGACAAGTCTGTCTGCCTATTCACCGATTTTGATTCTCAAACAAA<br>TGTGTCACAAAGTAAGGATTCTGATGTGTATATCACAGACAAAACTGTGCTAGACATGAGGTCTATGGACTTCAAGAGCAACAGTGCTGTGGCCTGGAGCAACAAATCCGACTTTGCATGTGCAAACGCCTTCA<br>ACAACAGCATTATTCCAGAAGACACCTTCTTCCCCAGCCCAGAAAGTTCCTGTGATGTCAAGCTGGTCGAGAAAAGCTTTGAAACAGATACGAACCTAAACTTTCAAAACCTGTCAGTGATTGGGTTCCGAATCC<br>TCCTCCTGAAAGTGGCCGGGTTTAATCTGCTCATGACGCTGCGGCTGTGGTCCAGCTAACTCGAGTGTTTAAACCAGCCTCAAGAACACCCGAATG
gp100-A2, TCRβ	ACTTGTTCTTTTTGCATCACAACCAGGCCTCCACAACCATGGGACCTCAGCTGCTGGGATACGTGGTGCTGTGTCTGCTTGGAGCCGGACCTCTGGAAGCCCAAGTGACACAGAACCCCAGATACCTGATCAC<br>CGTGACCGGCAAGAAACTGACCGTGACATGCAGCCAGAACATGAACCACGAGTACATGAGCTGGTACAGACAGGACCCTGGCCTGGGCCTGAGACAGATCTACTACAGCATGAACGTGGAAGTGACCGACAA<br>GGGCGACGTGCCCGAGGGCTACAAGGTGTCCAGAAAAGAGAAGCGGAACTTCCCACTGATCCTGGAAAGCCCATCTCCTAACCAGACCAGCCTGTACTTCTGCGCCAGCTCTCTGGGCAGCAGCTACGAGCA<br>GTATTTCGGCCCTGGCACCAGACTGACAGTGACCGAGGACCTGAAGAACGTGTTCCCACCTGAGGTGGCCGTGTTCGAGCCTTCTGAGGCCGAGATCAGCCACACACAGAAAGCCACACTCGTGTGTCTGGC<br>CACCGGCTTCTATCCCGATCACGTGGAACTGTCTTGGTGGGTCAACGGCAAAGAGGTGCACAGCGGCGTCAGCACAGATCCCCAGCCTCTGAAAGAACAGCCCGCTCTGAACGACAGCCGGTACTGTCTGAG<br>CAGCAGACTGAGAGTGTCCGCCACCTTCTGGCAGAACCCTCGGAACCACTTCAGATGCCAGGTGCAGTTCTACGGCCTGAGCGAGAACGATGAGTGGACCCAGGATAGAGCCAAGCCTGTGACTCAGATCGT<br>GTCTGCCGAAGCCTGGGGCAGAGCCGATTGTGGCTTTACCAGCGAGAGCTACCAGCAGGGCGTGCTGTCTGCCACAATCCTGTACGAGATCCTGCTGGGCAAAGCCACTCTGTACGCCGTGCTGGTTTCTGC<br>CCTGGTGCTGATGGCAATGGTCAAGCGGAAGGACAGCAGAGGCTAACTCGAGTGTTTAAACCAGCCTCAAGAACACCCGAATG
NY-ESO-1-A2, TCRα	ACTTGTTCTTTTTGCATCACAACCAGGCCTCCACAACCATGGAGACCCTGCTGGGCCTCCTTATACTGTGGCTTCAACTCCAATGGGTTAGCTCAAAGCAGGAAGTGACGCAAATTCCGGCAGCGTTGTCTGTTC<br>CAGAAGGTGAAAACCTTGTACTTAATTGTAGCTTCACAGACTCTGCGATTTACAATCTTCAGTGGTTTCGCCAAGACCCTGGGAAGGGTCTTACATCACTGCTCCTGATTCAATCTTCACAGCGCGAACAAACTA<br>GCGGGCGGCTTAACGCCTCACTTGATAAGAGCAGTGGCCGCTCCACTCTCTATATAGCGGCGTCTCAGCCGGGGGACTCTGCGACGTATTTGTGTGCTGTGCGACCACAAACTGGCGGGTCCTACATCCCAAC<br>ATTCGGAAGAGGCACCTCACTGATAGTACATCCAAATATACAGAATCCAGATCCCGCAGTATACCAACTTCGAGATAGTAAATCTAGTGACAAGTCCGTCTGTTTGTTTACAGATTTTGACTCCCAGACAAACGT

	CAGCCAGTCCAAGGACTCTGACGTCTATATCACAGACAAGACAGTGCTTGATATGCGGTCAATGGACTTCAAAAGCAACAGCGCCGTCGCATGGTCCAATAAGTCAGATTTCGCCTGTGCGAACGCGTTTAACA<br>ACTCCATAATCCCAGAGGATACCTTTTTCCCCAGTCCCGAGAGTTCATGTGACGTAAAGCTGGTAGAAAAAAGTTTCGAAACTGACACTAACCTCAACTTTCAAAATCTCAGTGTTATCGGCTTTCGGATTCTGTT<br>GTTGAAAGTGGCTGGATTCAATCTCCTGATGACACTTAGGCTGTGGAGTAGTTAACTCGAGTGTTTAAACCAGCCTCAAGAACACCCGAATG
NY-ESO-1-A2, TCRβ	ACTTGTTCTTTTTGCATCACAACCAGGCCTCCACAACCATGGCCACTACAATGGCGCCACGCCTCCTTTGCTGCGCTGCTTTGTCACTGCTTTGGGCAGGGCCGGTCAATGCCGGAGTAACCCAGACACCGAAA<br>TTCCAGGTGTTGAAAACCGGTCAGTCTATGACGTTGCAGTGCGCTCAAGATATGAACCATGAGTATATGTCCTGGTATAGGCAAGATCCAGGGATGGGTTTGCGACTCATCCATTATAGTGTGGGCGCCGGAAT<br>CACTGATCAGGGTGAGGTGCCGAACGGATATAACGTTAGTCGAAGTACGACGGAAGACTTTCCATTGAGGCTGCTTTCTGCGGCTCCTTCCCAGACGAGTGTGTACTTCTGCGCGTCATCTTATGTGGGGGCG<br>GCAGGGGAGCTGTTTTTCGGCGAAGGCTCACGGCTTACAGTGCTGGAAGACTTGAAAAATGTTTTTCCACCAGAAGTAGCGGTATTTGAACCGTCAGAGGCTGAAATCAGCCATACACAGAAGGCAACTCTGG<br>TTTGCTTGGCCACGGGCTTCTACCCTGATCACGTAGAGTTGTCCTGGTGGGTGAATGGAAAAGAAGTACACTCTGGTGTTTCCACCGACCCGCAACCGCTCAAGGAACAGCCAGCCCTTAACGATTCCCGGTAT<br>TGTCTGTCTTCAAGGCTGCGAGTATCCGCTACGTTCTGGCAAAATCCTCGAAATCACTTTCGGTGCCAAGTTCAATTCTATGGCCTTAGCGAGAATGACGAGTGGACTCAGGATCGAGCCAAACCCGTTACCCA<br>GATTGTCAGTGCCGAGGCTTGGGGTCGCGCTGACTGTGGTTTCACCTCTGAAAGCTACCAACAGGGTGTTCTTTCCGCTACAATACTGTATGAAATTCTCCTCGGCAAAGCGACGTTGTATGCGGTTTTGGTAT<br>CTGCCCTCGTTCTTATGGCAATGGTCAAGAGAAAAGATAGCCGAGGCTAACTCGAGTGTTTAAACCAGCCTCAAGAACACCCGAATG
MAGE-A3-DP4, TCRα	ACTTGTTCTTTTTGCATCACAACCAGGCCTCCACCACCATGGCCTGTCCTGGATTTCTGTGGGCCCTCGTCATCTCTACCTGTCTGGAATTCAGCATGGCCCAGACCGTCACACAGAGCCAGCCTGAGATGTCTG<br>TGCAGGAGGCCGAGACAGTGACCCTGAGCTGCACCTACGATACAAGCGAGAGCGACTACTACCTGTTTTGGTACAAGCAGCCTCCTTCACGGCAGATGATCCTGGTCATCCGTCAAGAAGCCTATAAGCAGCA<br>GAATGCCACCGAGAACCGTTTCAGCGTGAATTTCCAGAAGGCCGCCAAGAGCTTCAGCCTGAAGATCAGCGATAGCCAGTTGGGAGATGCCGCTATGTATTTCTGTGCCCTGAGATCCTCCGGAACCTACAAG<br>TACATCTTCGGCACCGGTACCAGACTTAAGGTGCTGGCCAATATTCAGAACCCCGAGCCTGCCGTCTACCAGCTGAAGGATCCTAGAAGCCAGGATAGCACCCTGTGCCTGTTCACCGATTTCGACAGCCAGAT<br>TAACGTCCCCAAGACCATGGAATCCGGCACCTTCATCACAGATAAGACCGTTCTGGACATGAAAGCCATGGACTCCAAGAGCAATGGAGCCATTGCTTGGTCCAATCAAACCTCCTTCACCTGCCAGGATATCT<br>TCAAAGAGACAAACGCTACCTATCCTAGCTCCGATGTGCCCTGTGATGCCACCCTGACCGAGAAGTCCTTCGAGACAGATATGAACCTGAATTTCCAGAACCTGTCTGTCATGGGCCTGAGAATCCTGCTGCTG<br>AAGGTCGCCGGATTCAACCTGCTGATGACACTTAGACTGTGGTCCAGCTGACTCGAGTGTTTAAACCAGCCTCAAGAACACCCGAATG
MAGE-A3-DP4, TCRβ	AATACAAGCTACTTGTTCTTTTTGCATCACAACCAGGCCTCCACCACCATGGGAACCAGACTGCTGTTCTGGGTCGCCTTCTGTCTGCTGGGAGCCGATCATACAGGTGCTGGTGTCTCCCAGTCCCCCTCCAA<br>CAAGGTCACCGAGAAGGGCAAAGATGTGGAACTGCGATGTGACCCCATCAGCGGACACACAGCTCTGTACTGGTACAGACAGTCTCTCGGCCAGGGCCTCGAGTTCCTGATCTATTTCCAGGGAAACAGCGC<br>CCCTGACAAGTCAGGACTGCCTAGCGATAGATTCTCAGCTGAACGTACAGGAGGCTCCGTTTCCACACTGACCATCCAGAGAACCCAGCAAGAGGACTCTGCTGTGTACCTGTGTGCTTCTATCAGAACCGGC<br>CCTTTCTTCTCCGGCAATACAATCTACTTTGGCGAAGGCTCCTGGCTGACCGTGGTGGAAGATCTGAGAAATGTGACCCCACCTAAGGTTTCCCTGTTTGAACCTAGCAAGGCCGAGATCGCTAACAAGCAGAA<br>GGCTACCCTGGTCTGCCTGGCTAGAGGATTCTTTCCCGATCATGTTGAGCTGTCTTGGTGGGTCAACGGAAAGGAGGTCCACAGCGGAGTCTCTACAGATCCCCAGGCTTACAAAGAGTCCAACTACAGCTAC<br>TGCCTGAGCAGCAGACTGAGAGTGTCCGCCACCTTCTGGCACAACCCCAGAAACCATTTCAGATGCCAGGTGCAGTTCCACGGCCTGAGCGAAGAGGATAAGTGGCCTGAGGGATCTCCCAAGCCTGTGACA<br>CAAAATATCTCTGCAGAAGCTTGGGGAAGAGCTGATTGTGGAATCACCAGCGCCAGCTACCATCAGGGCGTTCTGTCCGCCACAATCCTGTATGAGATCCTTCTGGGCAAAGCTACTCTCTACGCTGTGCTGGT<br>GTCTGGCCTGGTGCTGATGGCCATGGTCAAGCGTAAGAACTCCTGACTCGAGTGTTTAAACCAGCCTCAAGAACACCCGAATG

Supplementary Figures

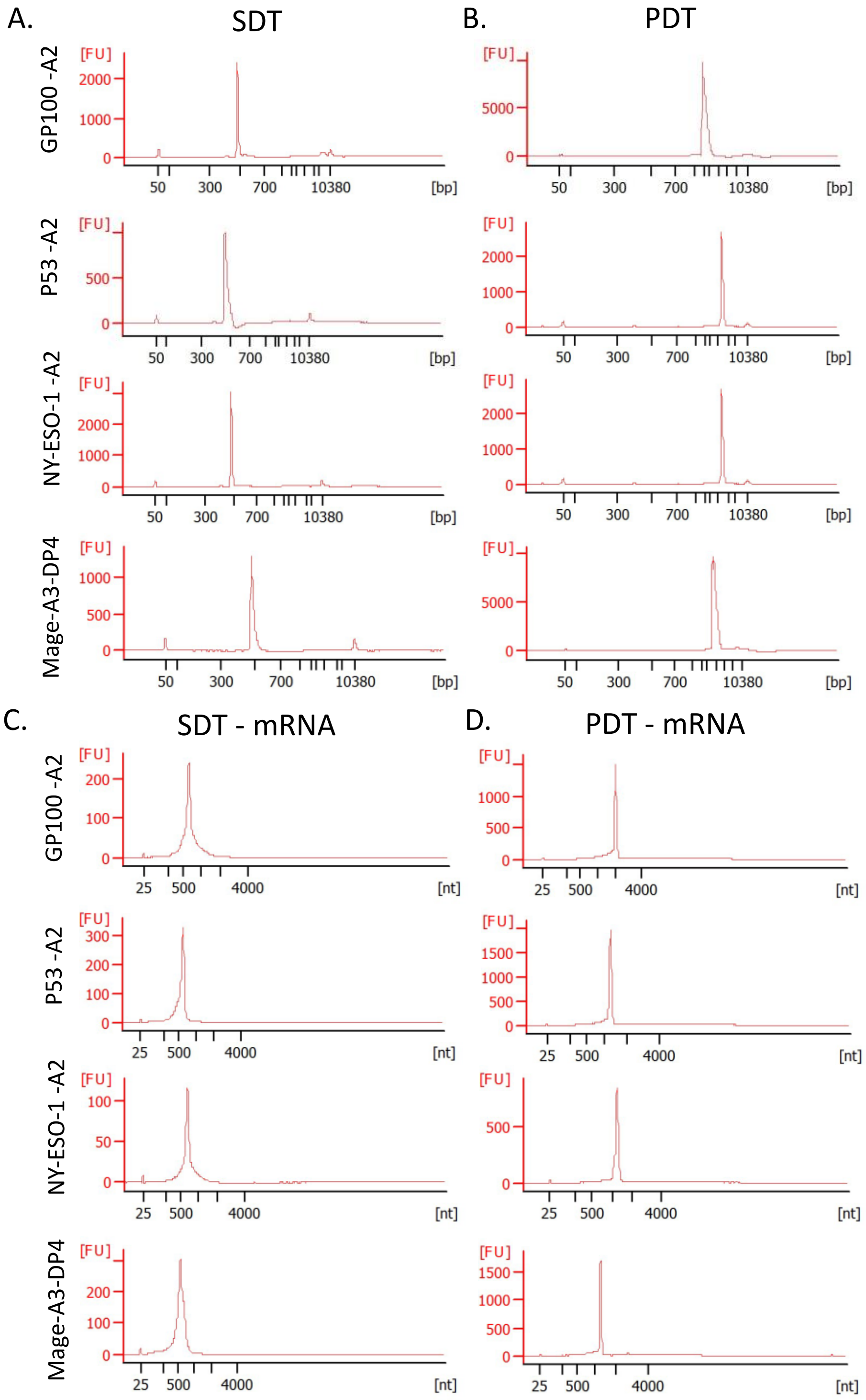

Figure S1: Representative capillary gel electropherograms. (A) SDT, (B) PDT, (C) SDT-mRNA and (D) PDT-mRNA encoding the HLA-A2-restricted epitopes of gp100 (YLEPGPVTA), NY-ESO-1 (SLLMWITQC) and p53 (LLGRNSFEV) and the HLA-DP4-restricted epitope of MAGE-A3 (TQHFVQENYLEY).

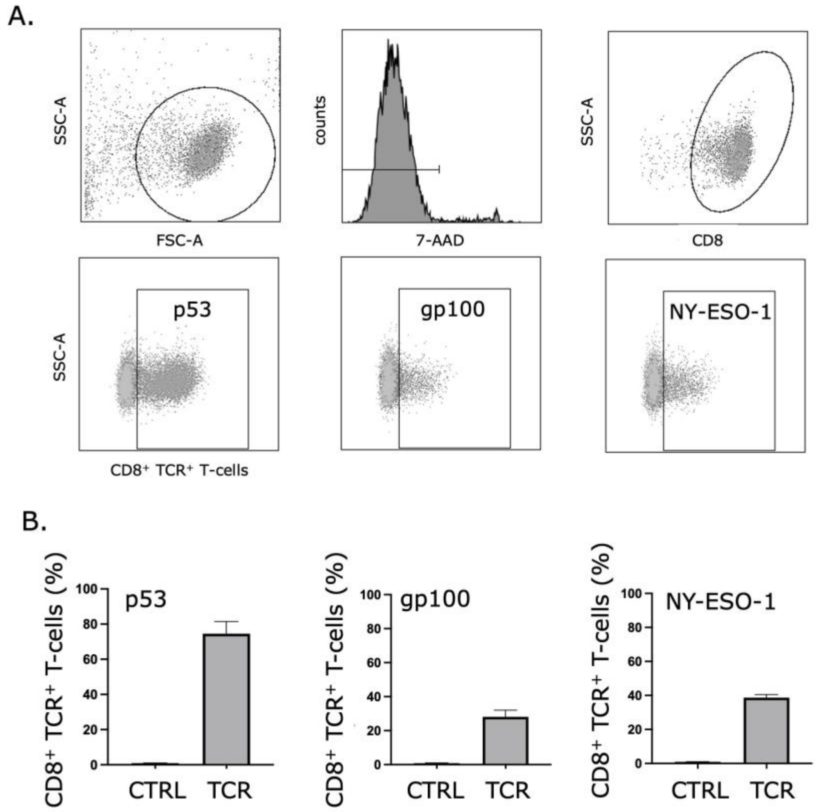

Figure S2: Expression of T-cell receptors on CD8⁺ T-cells after electroporation with mRNA encoding the T-cell receptor alpha and beta chain. (A) Representative flow cytometry graphs showing the gating strategy. We gated on viable cells based on forward (FSC-A) and side (SSC-A) scatter characteristics as well as absence of 7-AAD. We subsequently gated on cells that were bound by anti-CD8 antibodies and within this $CD8^+$ T-cell population we gated on cells that bound to dextramers specific for the introduced TCR. Three independent experiments were performed. (B) The graphs show the percentage of $CD8^+$ TCR^+ T-cells as detected in flow cytometry. The results are shown as mean ± standard error of the mean and summarize three independent experiments. Statistical analysis was not performed.

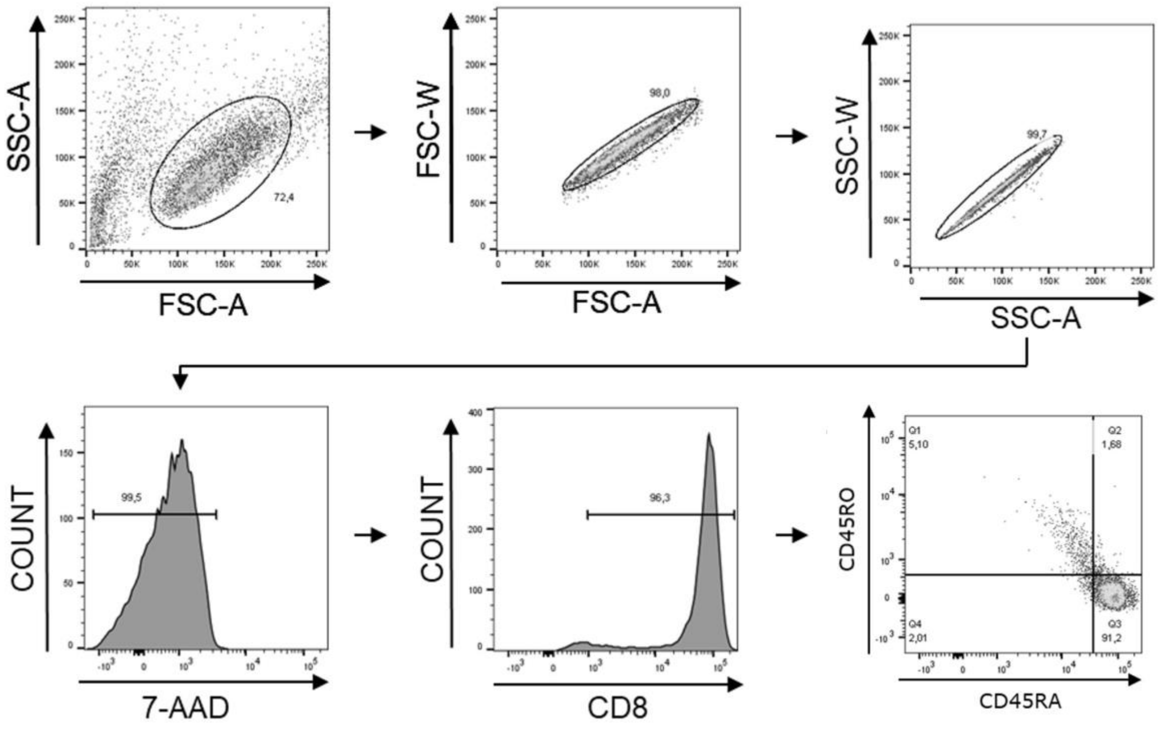

Figure S3: Selection of naïve (CD45RA⁺) CD8⁺ T-cells for neoantigen immunogenicity screening. Representative flow cytometry graphs showing the gating strategy. We gated on single viable cells based using forward (FSC-A/W) and side (SSC-A/W) scatter characteristics as well as absence of 7-AAD. We subsequently gated on cells that were bound by anti-CD8 antibodies and within this CD8⁺ T-cell population we gated on cells that are CD45RA⁺. Three independent experiments were performed.
